# Selective capture of carbon dioxide from hydrocarbons using a metal-organic framework

**DOI:** 10.1038/s41467-020-20489-2

**Published:** 2021-01-08

**Authors:** Omid T. Qazvini, Ravichandar Babarao, Shane G. Telfer

**Affiliations:** 1 MacDiarmid Institute for Advanced Materials and Nanotechnology, School of Fundamental Sciences, Massey University, Palmerston North, New Zealand; 2 Department of Chemical Engineering and Analytical Science, The University of Manchester, Oxford Road, Manchester, M13 9PL UK; 3 School of Science, RMIT University, Melbourne, VIC 3001 Australia; 4 Commonwealth Scientific and Industrial Research Organisation (CSIRO) Manufacturing, Clayton, VIC 3169 Australia

**Keywords:** Coordination chemistry, Environmental chemistry, Metal-organic frameworks

## Abstract

Efficient and sustainable methods for carbon dioxide capture are highly sought after. Mature technologies involve chemical reactions that absorb CO_2,_ but they have many drawbacks. Energy-efficient alternatives may be realised by porous physisorbents with void spaces that are complementary in size and electrostatic potential to molecular CO_2_. Here, we present a robust, recyclable and inexpensive adsorbent termed MUF-16. This metal-organic framework captures CO_2_ with a high affinity in its one-dimensional channels, as determined by adsorption isotherms, X-ray crystallography and density-functional theory calculations. Its low affinity for other competing gases delivers high selectivity for the adsorption of CO_2_ over methane, acetylene, ethylene, ethane, propylene and propane. For equimolar mixtures of CO_2_/CH_4_ and CO_2_/C_2_H_2_, the selectivity is 6690 and 510, respectively. Breakthrough gas separations under dynamic conditions benefit from short time lags in the elution of the weakly-adsorbed component to deliver high-purity hydrocarbon products, including pure methane and acetylene.

## Abstract (web summary)

Metal-organic framework adsorbents are promising materials for gas separation and purification. Herein, the authors present a metal-organic framework that selectively captures CO_2_ over small hydrocarbons; this separation is relevant for the purification of natural gas and industrial feedstocks.

## Introduction

Chemical separation processes consume vast quantities of energy^1^. Economical and practical pathways to alleviating this burden are required. This is especially relevant to the capture of CO_2_, which is a common impurity in crude gas streams. CO_2_ removal is integral to upgrading natural gas and biogas, for example, and to the purification of valuable hydrocarbons prior to polymerisation or chemical derivatization^2^. These processes are separations that rely on discrimination between CO_2_ and other gases. One established technology is to trap the CO_2_ by a chemical reaction with an absorbent. This typically involves chemisorption to an amine in aqueous solution^3,4^. Chemisorption incurs multiple drawbacks, however, including a high energy penalty during regeneration, amine losses due to degradation and evaporation, and the corrosion of hardware and pipelines^5^. Other conventional separation methods involve solvent extraction or cryogenic distillation, which are burdened with a high energy penalty and large amount of solvent waste.

The physisorption of CO_2_ in nanoporous materials is an attractive alternative^6,7^. Physisorption is governed by weak, noncovalent bonding interactions in pores that are structured on the molecular scale^8^. Ideally, they lower the energy requirements for regeneration since driving off the trapped CO_2_ simply involves breaking interactions that are inherently weak. Effective physisorbents combine rapid guest diffusion, recyclability and long-term stability with selectivity for CO_2_ over competing gases at relevant concentrations^9^. Thus, they may offer a sustainable solution to CO_2_ capture. In this context, metal-organic frameworks (MOFs) have risen to prominence^10–14^. MOF materials are built up from metal ions and organic ligands, and their pore shape, size and chemical environment can be systematically designed^15,16^. In turn, this allows interactions between framework hosts and molecular guests to be tailored. In the search of effective MOF physisorbents, simply searching for materials with ever-higher levels of CO_2_ uptake per se may not deliver adsorbents that are adept at gas separations since the adsorption of non-CO_2_ components may also increase. Instead, significant advances will emerge by suppressing the uptake of these competing gases^17,18^, developing scalable synthetic protocols, mitigating the impact of common impurities such as water vapour and oxygen, and developing low energy pathways to adsorbent recycling.

The removal of CO_2_ from hydrocarbons is an important process^2^. While natural gas and biogas are primarily composed of methane (at high pressure and low pressure, respectively), contamination by CO_2_ can prevent optimal heat release from gas combustion, and cause pipeline corrosion and dry ice formation^19^. MOFs, however, offer a means of reducing the CO_2_ concentration in the presence of dominant quantities of methane^10,20,21^. Acetylene (C_2_H_2_) is an essential feedstock for the industrial production of commodity materials^22,23^. When acetylene is generated, however, it typically coexists with CO_2_ impurities^24^. The separation of C_2_H_2_ and CO_2_ is challenging due to their similar physical properties (Supplementary Table 4). MOF physisorbents offer a potential solution but most show an affinity toward C_2_H_2_ rather than CO_2_^11^. The selective adsorption of the CO_2_ component has seldom been reported despite its operational simplicity in process design and the promise of energy efficiency. Conversely, gas purification using hydrocarbon-selective MOFs requires additional stages if the eluent is contaminated by adsorbed CO_2_ during the desorption step^25^. Despite recent advances in MOF chemistry, challenges remain in producing framework adsorbents that combine good separation capabilities with wider performance characteristics such as scalability, recyclability and easy low-energy regeneration. MOF adsorbents that may be applied to methane purification and that preferentially adsorb CO_2_ from other hydrocarbons are in particular demand.

In this work, we present a MOF, termed MUF-16 (MUF = Massey University Framework) that exhibits inverse selectivity: the adsorption of carbon dioxide in preference to hydrocarbon guests. The carbon dioxide is efficiently sequestered by hydrogen bonding and a range of other favourable noncovalent interactions. This underpins high selectivities for the separation a range of gas mixtures that are relevant to natural gas and industrial feedstocks. Being economical to produce on scale, stable and recyclable, MUF-16 has many of the qualities of an attractive adsorbent.

## Results

### Synthesis and characterisation

**Fig. 1:**
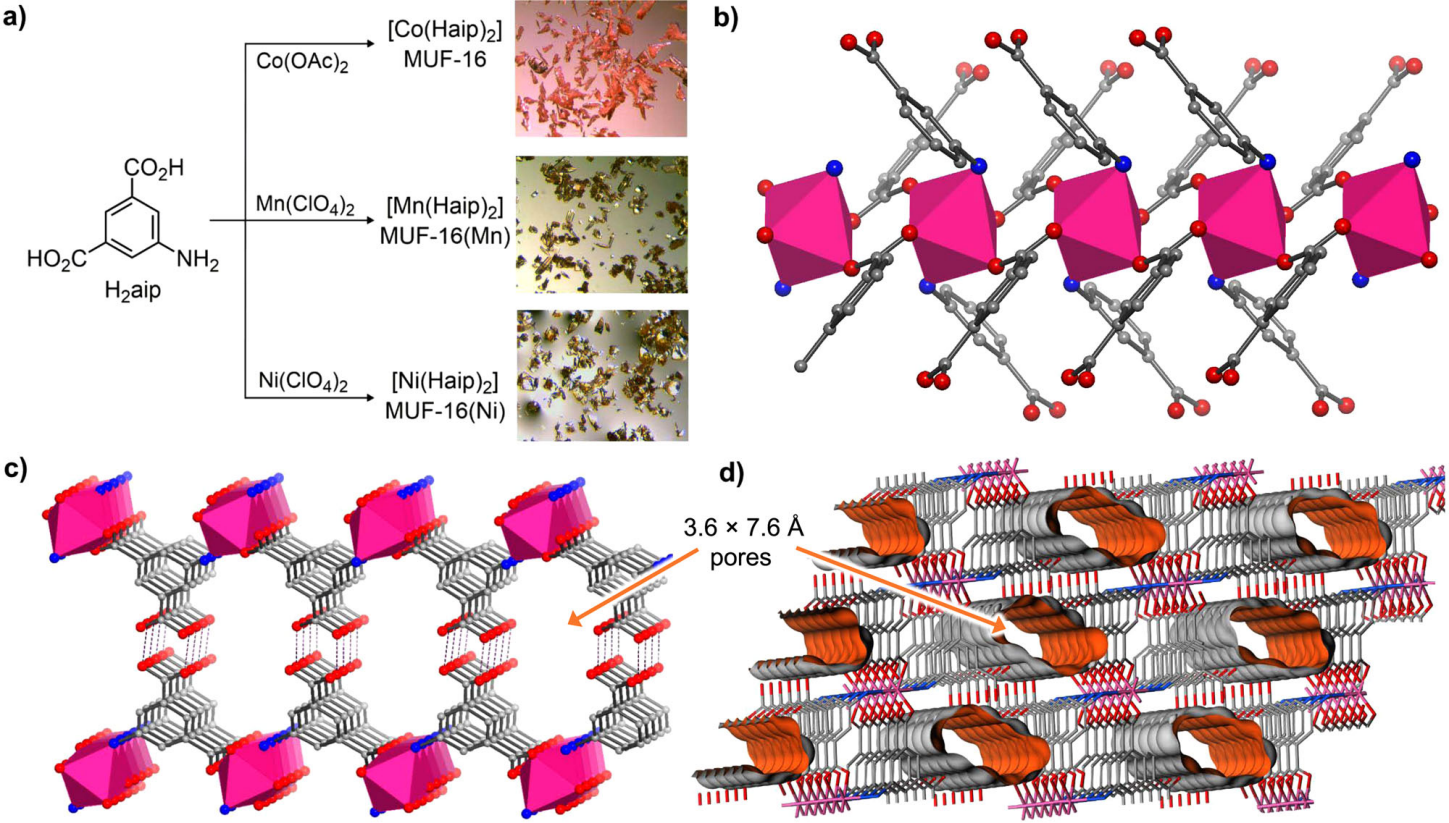
Synthesis and structure of MUF-16 materials. **a** Synthetic routes to the MUF-16 family and optical micrographs of the reaction products. **b** Infinite secondary building units (iSBUs) in MUF-16 comprise one-dimensional cobalt(II) chains connected by *μ*_2_-bridging carboxylate groups of the Haip ligands (H_2_aip = 5-aminoisophthalic acid). The cobalt(II) ions are depicted as filled octahedra. **c** The iSBUs are linked into planar two-dimensional sheets by the Haip ligands and further connected into a three-dimensional framework by hydrogen bonding (depicted as dashed lines) between adjacent sheets. **d** MUF-16 features one-dimensional channels with approximate dimensions of 3.6 × 7.6 Å that propagate through the framework. The Connolly surface of the framework is shown in orange and defined with a probe of diameter 1.0 Å. Colour code: Co = magenta; O = red; C = grey, N = blue.

Inspired by the superb properties of MOFs derived from straightforward and readily-available linkers^26,27^, our interest was captured by the MUF-16 series of materials. These frameworks are prepared by combining 5-aminoisophthalic acid (H_2_aip), an inexpensive, commercially-available linker, with cobalt(II), nickel(II), or manganese(II) salts in methanol (Fig. 1a). This delivers compounds with the general formula [M(Haip)_2_]^28,29^, referred to as MUF-16 (M = Co), MUF-16(Ni) and MUF-16(Mn), respectively. These easily-handled crystalline materials are high yielding on gram scales and tolerant to oxygen and water vapour. Their crystal structures were determined by single crystal X-ray diffraction (Supplementary Table 1). The three frameworks are isostructural, belonging to the *I*2/a space group. Individually, the metal ions adopt an octahedral geometry with four carboxylate and two amino donors arranged *trans* to one another. These ions are aligned into one-dimensional chains along a crystallographic axis supported on each side by μ_2_-bridging carboxylate groups (Fig. 1b). Adjacent chains are connected into two-dimensional sheets by Haip ligands that extend across the plane by coordinating to adjacent one-dimensional chains with both their amino and carboxylate donors (Fig. 1b). Only one of the two carboxyl groups of each Haip ligand coordinates to the metal. The other remains protonated and engages in hydrogen-bonding with a partner from an adjacent layer (Fig. 1c). These interactions link the layers into three-dimensional frameworks. The frameworks support one-dimensional channels of approximately 3.6 × 7.6 Å (accounting for the van der Waals surfaces of the atoms, Fig. 1d). In their as-synthesised form the pores contain occluded water, which can be easily removed by heating at 130 °C in vacuo.

Thermogravimetric analysis demonstrated the thermal stability of the MUF-16 materials beyond 330 °C (Supplementary Fig. S2). Their purity was established by both elemental analysis and powder X-ray diffraction (Supplementary Fig. S5). The frameworks are chemically robust, being unaffected by soaking in water or exposure to humid air for prolonged periods, as confirmed by powder X-ray diffraction and gas adsorption analysis (vide infra and Supplementary Figs. S6–S8, S13a).

As suggested by pore evident in their SCXRD structures, the MUF-16 frameworks are accessible to a range of incoming gases. Nitrogen adsorption isotherms measured at 77 K gave BET surface areas of 214, 205 and 204 m^2^/g for MUF-16, MUF-16(Mn), and MUF-16(Ni), respectively (Supplementary Figs. S19–S21). Total pore volumes of 0.11 cm^3^/g were established for all three frameworks (Supplementary Table 3). These values are comparable with the geometric surface areas and pore volumes calculated from the crystallographic coordinates. The pore size distribution of MUF-16 also was calculated, which is consistent with the pore dimensions observed by SCXRD (Supplementary Fig. S12).

### Gas adsorption measurements

**Fig. 2:**
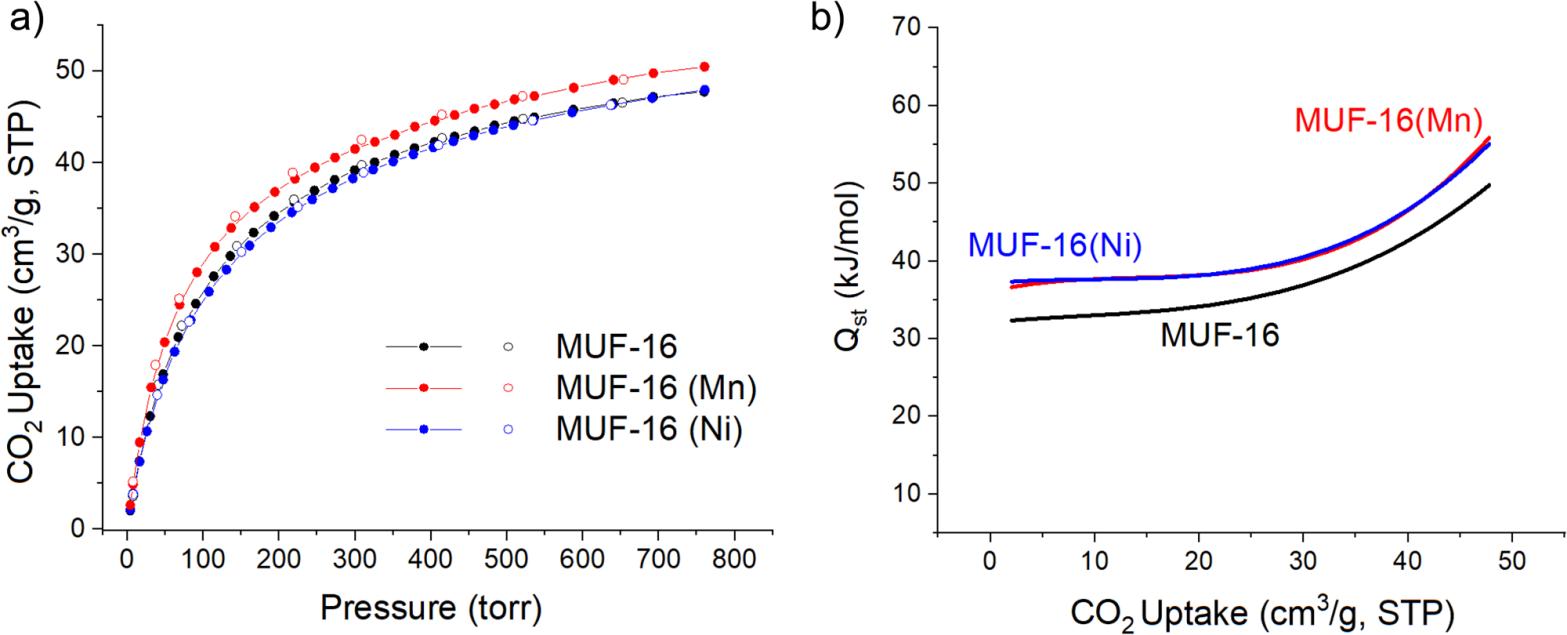
CO_2_ adsorption on MUF-16 materials. **a** Volumetric adsorption (filled circles) and desorption (open circles) isotherms of CO_2_ at 293 K and for MUF-16 (black), MUF-16(Mn) (red), and MUF-16(Ni) (blue). **b** Heats of adsorption (*Q*_*st*_) calculated for CO_2_ binding to MUF-16 (black), MUF-16(Mn) (red), and MUF-16(Ni) (blue) as a function of CO_2_ uptake. A high affinity for CO_2_ coupled to a moderate heat of adsorption promise an adsorbent that takes up significant quantities of gas yet is easily recycled. Source data are provided as a Source Data file.

CO_2_ isotherms were collected at 293 K and up to 1 bar (Fig. 2a and see Supplementary Fig. S11 for other temperatures). Both MUF-16 and MUF-16(Ni) take up 2.13 mmol/g (48 cm^3^/g) at 1 bar, and MUF-16(Mn) adsorbs 2.25 mmol/g (50.5 cm^3^/g). This equates to approximately 0.9 molecules of CO_2_ per metal site (Supplementary Table 5). CO_2_ uptake is only marginally higher at 273 K (Supplementary Fig. S11). The isosteric heat of adsorption (*Q*_*st*_) at zero-coverage was calculated to be 32 kJ/mol for MUF-16 and 37 kJ/mol for its Ni and Mn analogues (Fig. 2b). The *Q*_*st*_ increases at higher loadings, which can be attributed to attractive intermolecular interactions when the CO_2_ loading levels are high, which enhance the framework-CO_2_ affinity. These interactions were experimentally verified by SCXRD (vide infra). The moderate *Q*_*st*_ values, even at high CO_2_ loading^30^, are well below values observed for MOFs with open metal sites^31^. It follows that the energy required to regenerate the frameworks by CO_2_ desorption is likely to be low.

**Fig. 3:**
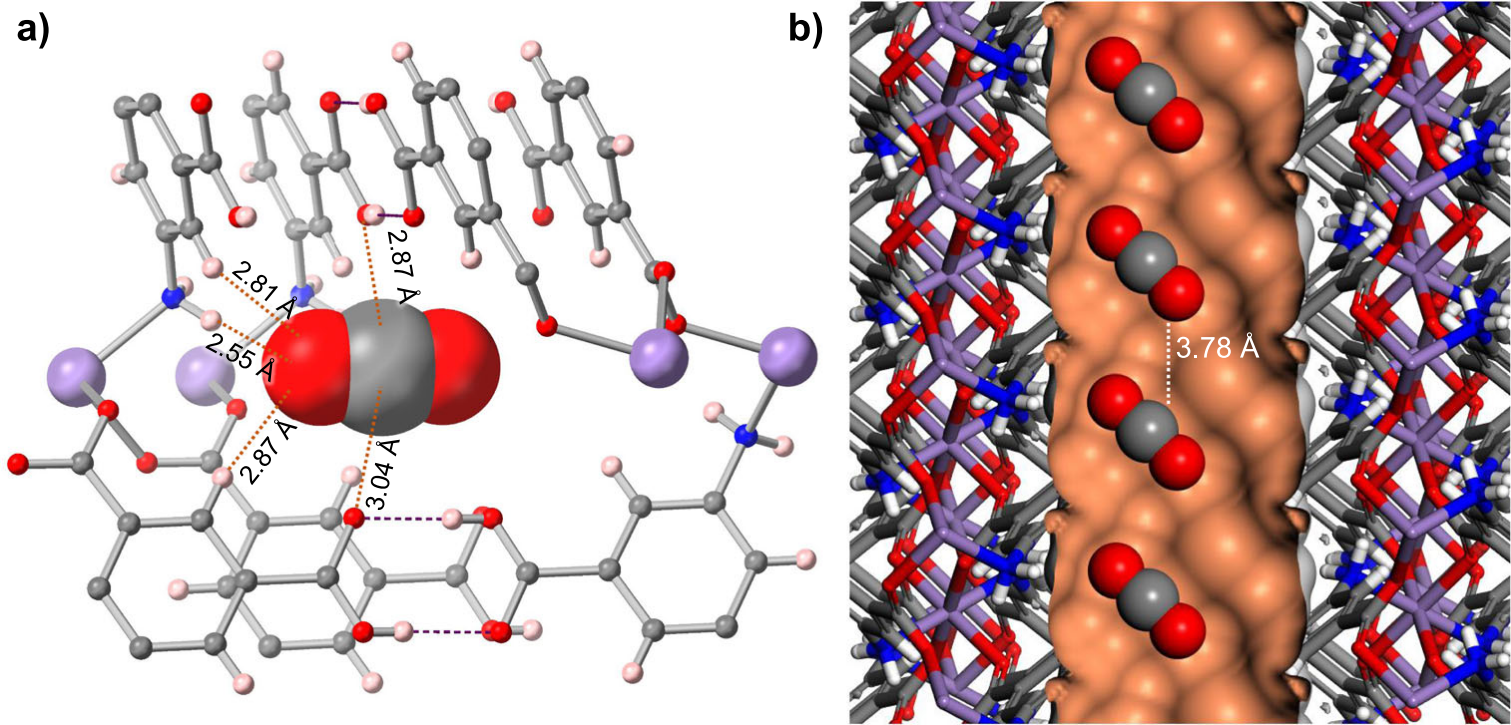
CO_2_ capture by MUF-16. **a** The adsorption sites of CO_2_ molecules in the pores of MUF-16(Mn), as determined by single-crystal X-ray diffraction. The CO_2_ is depicted in space-filling mode. Key intermolecular distances between MUF-16(Mn) and the adsorbed CO_2_ are shown with dashed orange lines. A second, symmetry-equivalent CO_2_ adsorption site exists. **b** Adsorbed CO_2_ molecules in MUF-16(Mn) highlighting the arrangement of adsorbed CO_2_ in the framework channels and potential attractive noncovalent interactions between adjacent guests. The CO_2_ molecules are shown in representative orientations in one of two symmetry-related crystallographic orientations. Colour code: manganese = lilac; nitrogen = blue; oxygen = red; carbon = grey; hydrogen = pale pink or white; pore Connolly surface = orange.

Single-crystal X-ray diffraction was used to identify the CO_2_ binding sites in these frameworks^32,33^. MUF-16(Mn) was selected for this study since its darker colour streamlined crystal handling (the pale colour of the Co(II) and Ni(II) analogues make them difficult to see when loaded in a glass capillary). The results obtained for MUF-16(Mn) are directly applicable to MUF-16 and MUF-16(Ni) due to their identical structures and CO_2_ adsorption profiles (Fig. 2a and Supplementary Fig. S5). After transferring a MUF-16(Mn) single crystal into a capillary, it was activated in vacuo and the capillary flame-sealed. This allowed the guest-free structure of MUF-16(Mn) to be determined crystallographically (Supplementary Table 2). We then filled CO_2_ into the capillary to a pressure of 1.1 bar to determine the structure of the CO_2_-loaded framework. We noted only minor changes to the framework itself upon evacuation and filling with CO_2_. A clear picture of the affinity of MUF-16 for CO_2_ arises from the CO_2_-loaded SCXRD structure. First, the dimensions of the framework pores are well matched to the size of the CO_2_ molecules. This allows the guests to be enveloped by multiple non-covalent contacts (Fig. 3a). Second, these contacts are favourable since the electric quadrupole of the CO_2_ is complementary to the polarisation of the MUF-16 pore surface. For example, one of the electronegative oxygen atoms of each CO_2_ molecule engages in N-H···O and C-H···O hydrogen bonds with framework amino and phenyl groups at distances of 2.55, 2.81, and 2.87 Å. The electropositive carbon atom of each CO_2_ molecule engages in close-range contacts with the oxygen atoms of two non-coordinated carboxyl groups (2.87 and 3.04 Å). Two sites, which are related by crystallographic symmetry and share a common location for one of the oxygen atoms, are available to the CO_2_ guests. They are occupied with a 50/50 ratio and refinement of the CO_2_ occupancies gave 0.77 CO_2_ molecules per Mn centre, which agrees with the adsorption isotherm (Supplementary Table 5) allowing for uncertainties in the exact CO_2_ pressure in the X-ray capillaries. The CO_2_ guest molecules are aligned along the channels and tilted with respect to the pore axis (Fig. 3b). Attractive C···O intermolecular interactions between adjacent molecules are evident at a distance of 3.78 Å. This array of CO_2_ guests probably underlies the observed increase in *Q*_*st*_ as a function of gas loading observed in the adsorption isotherms. A computational DFT model agrees with the SCXRD structure (Supplementary Fig. S60).

The strong adsorption of nitrous oxide, N_2_O, by MUF-16 corroborates this model of CO_2_ binding. The size and electrostatic distribution of N_2_O closely match those of CO_2_ (Supplementary Fig. S9). In parallel with CO_2_, N_2_O possesses atoms with partial negative charges at its termini that can bind to positively-charged regions of the pore surface, and vice-versa for its central nitrogen atom. MUF-16 adsorbs 1.91 mmol/g (43 cm^3^/g) of N_2_O at 1 bar and 293 K, which is only slightly less than the uptake of CO_2_.

**Fig. 4:**
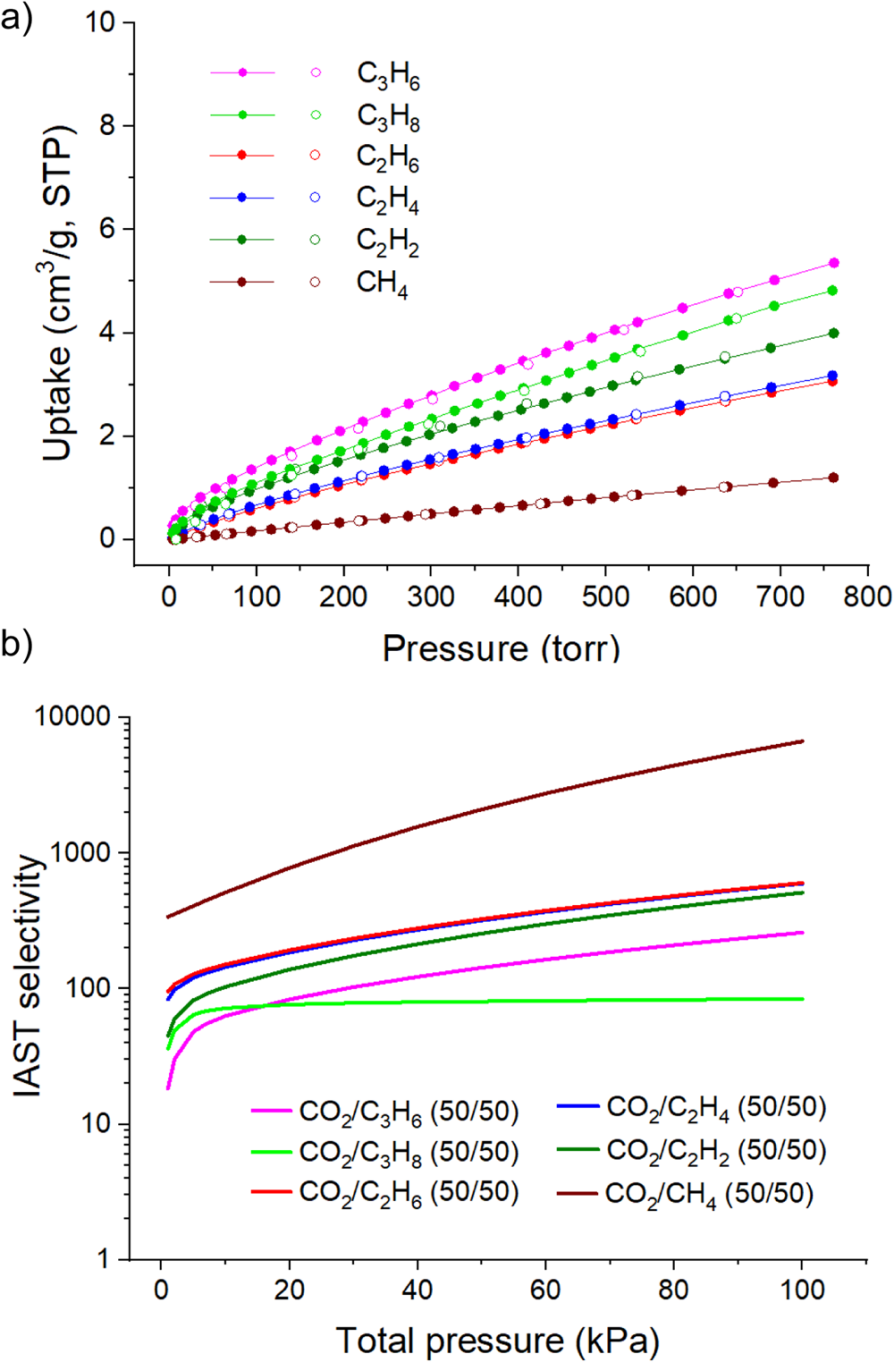
Gas uptake and calculated separation by MUF-16. **a** Experimental CH_4_, C_2_H_2_, C_2_H_4_, C_2_H_6_, C_3_H_6_ and C_3_H_8_ adsorption (solid spheres) and desorption (open spheres) isotherms of MUF-16 measured at 293 K. **b** Predicted IAST selectivities, displayed with a log scale, of MUF-16 for various gas mixtures at 293 K. Source data are provided as a Source Data file.

The high uptake of CO_2_ by MUF-16 contrasts with its low affinity for hydrocarbons. Adsorption isotherms of CH_4_, C_2_H_2_, C_2_H_4,_ C_2_H_6_, C_3_H_6_ and C_3_H_8_ were measured on MUF-16 at 293 K (Fig. 4a and Table 1). MUF-16 takes up just 1.20 cm^3^/g of CH_4_ at 1 bar and 293 K and 3.99 cm^3^/g of C_2_H_2_. The highest adsorption amount was 5.35 cm^3^/g observed for C_3_H_6_. Since only modest quantities of these gases are adsorbed, care was taken to ensure the accuracy of these measurements by using large sample quantities. The *Q*_*st*_ values for the hydrocarbon gases are much lower than for CO_2_ (Supplementary Table 6). The water vapour adsorption isotherm of MUF-16 was measured at 298 K, showing the steady uptake of water until saturation is reached at around two molecules per Co centre (Supplementary Fig. S13b). The isotherm is fully reversible indicating that the adsorbed water is easily removed without perturbation of the framework.

**Table 1:** Summary of gas adsorption data and IAST-calculated selectivities for the MUF-16 family at 1 bar and 293 K.

	MUF-16	MUF-16(Mn)	MUF-16(Ni)
Q_st_			
CO_2_^*a*^	32.3	36.6	37.3
Uptake^*b*^			
CO_2_	47.78	50.5	47.97
CH_4_	1.20	3.10	2.77
C_2_H_2_	3.99	9.69	7.53
C_2_H_4_	3.17	8.31	5.42
C_2_H_6_	3.06	8.81	5.67
C_3_H_6_	5.35	—	—
C_3_H_8_	4.82	—	—
IAST selectivity			
CO_2_/CH_4_^*c*^	6690	470	1220
CO_2_/C_2_H_2_^*c*^	510	31	46
CO_2_/C_2_H_4_^*c*^	600	150	130
CO_2_/C_2_H_6_^*c*^	600	55	110
CO_2_/C_3_H_6_^*c*^	260	—	—
CO_2_/C_3_H_8_^*c*^	84	—	—

^*a*^In kJ/mol at zero loading.

^*b*^In cm^3^/g.

^*c*^50/50 ratio at 1 bar and 293 K as calculated by IAST.

Uptake ratios provide a useful indication of the preference of an adsorbent for certain gases over others. For MUF-16, the CO_2_/CH_4_ uptake ratio is 39.8 (293 K and 1 bar). This is comparable to [Cd_2_L(H_2_O)] (42.9)^34^ and exceeded by only one other reported material (SIFSIX-14-Cu-i, 85) (Supplementary Table 10)^35^. Typical physisorbents show a preference for unsaturated hydrocarbons over CO_2_, especially when bonding between the guest’s π electrons and open metal sites can occur^25,36–50^. However, MUF-16 exhibits a uniform preference for CO_2_ over all C2 and C3 hydrocarbons at 293 K and 1 bar (Table 1). Here, the uptake ratios fall between 12 (acetylene), 15.6 (ethane) and 8.9 (propene). While the limited uptake of CH_4_ is a well-established function of its small size and low polarizability, the low affinity of MUF-16 for larger and more polar/polarizable hydrocarbon guests is notable. Inverted selectivity of this kind, that is, a preference for CO_2_ over small hydrocarbons, is a sought after yet seldom reported phenomenon^25,51–57^. With an uptake ratio of 12, MUF-16 surpasses previously reported materials that preferentially adsorb CO_2_ over C_2_H_2_, including SIFSIX-3-Ni (1.2 at 298 K and 0.1 bar)^25^, CD-MOF-2 (1.3 at 298 K and 1 bar)^51^, K_2_[Cr_3_O(OOCH)_6_(4‐ethylpyridine)_3_]_2_[α‐SiW_12_O_40_] (4.5 at 278 K and 1 bar)^55^, [Mn(bdc)(dpe)] (6.4 at 273 K and 1 bar)^52^ and [Tm_2_(OH-bdc)_2_(μ_3_- OH)_2_(H_2_O)_2_]^58^ (2.8 at 298 K and 1 bar) (Supplementary Table 11). The diminished affinity of MUF-16 for C_2_H_2_ results from the reversed quadrupole moment of this guest vis-à-vis CO_2_ (Supplementary Fig. S10). Since C_2_H_2_ is polarised oppositely to CO_2_ it is electrostatically repelled by the functional groups that line binding pockets in MUF-16. The upshot is inverse selectivity for CO_2_ over acetylene.

### Separations using MUF-16

**Fig. 5:**
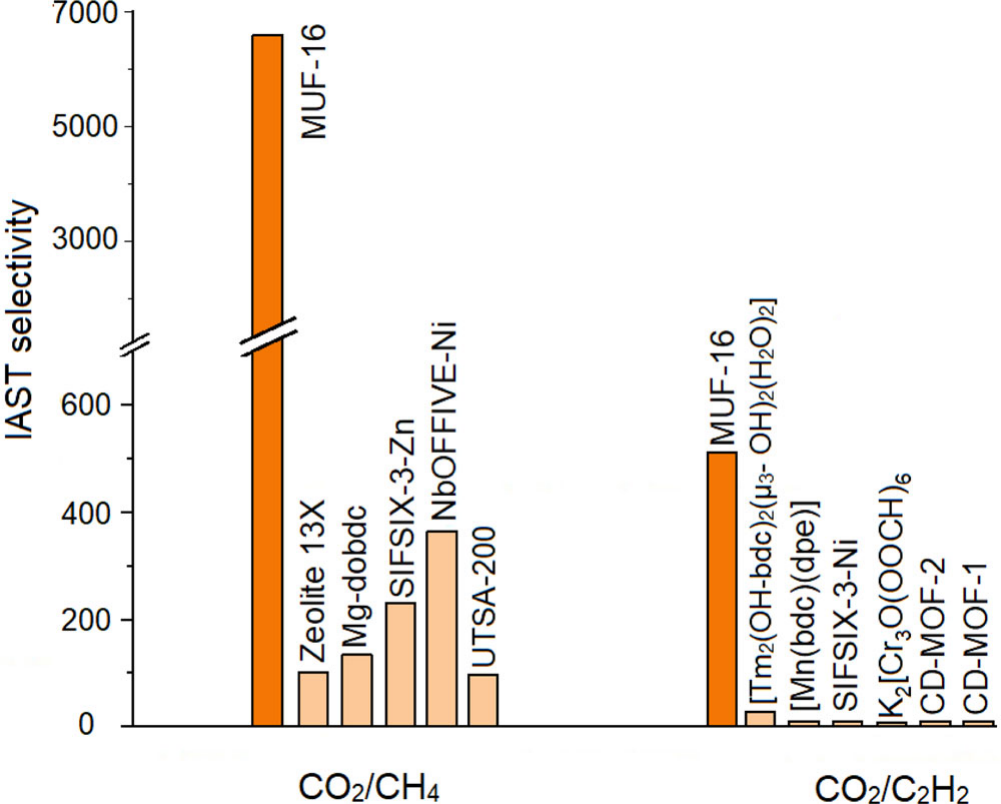
Separation performance of MUF-16 compared to top-performing materials. IAST selectivity of MUF-16 in comparison to a selection of physisorbents for CO_2_/CH_4_ (50/50) and CO_2_/C_2_H_2_ (50/50) mixtures at ambient temperature and 1 bar (see Supplementary Table 11 for details). For clarity, the y axis is broken in two parts with different scales.

Building on the preferential affinity indicated by the uptake ratios, we quantified the selectivity of MUF-16 by Ideal Adsorbed Solution Theory (IAST) calculations^59^. At 293 K and 1 bar, the IAST selectivity of MUF-16 for CO_2_ over CH_4_ (50/50 mixture) is 6690 (Fig. 4b). MUF-16 is thus the best physisorbent known for this separation that does not operate by molecular sieving (Fig. 5 and Supplementary Table 10). For equimolar mixtures of CO_2_ and C_2_H_2_, C_2_H_4,_ C_2_H_6_, C_3_H_6_ or C_3_H_8_ the selectivity of MUF-16 is also high (Table 1). With a selectivity of 510, MUF-16 is elevated well beyond other materials for the capture of CO_2_ from CO_2_/C_2_H_2_ (50/50) mixtures (Fig. 5 and Supplementary Table 11). As recognised in the literature for related systems^17,18,60^, these high selectivities emerge by suppressing the uptake of the hydrocarbon gases while maintaining proficient CO_2_ capture.

While the pore characteristics of MUF-16 clearly favour the uptake of CO_2_ over other gases, its affinity could potentially rely on molecular sieving if the larger adsorbates are excluded from the framework on the basis of their size. This was ruled out by measuring hydrocarbon adsorption isotherms at 195 K, which showed that MUF-16 can adsorb CH_4_, C_2_H_2_ and C_2_H_6_ (Supplementary Fig. S15). Guest molecules of this size can freely enter the pore network of MUF-16 at this low temperature. However, since uptake is low at ambient temperatures interactions of these gases with the framework must be weak. Further, the kinetics of adsorption of several guest molecules were measured (Supplementary Fig. S16). All gases display a similar kinetic profile and reach their equilibrium uptake in well under one minute. Therefore, thermodynamic—rather than kinetic—effects have the most decisive impact on the differential affinity of these gases for MUF-16. We also considered whether a structural change of the framework might underly the gas selectivity, as observed for related systems^52^. However, XRD measurements show that the framework structure is largely conserved around room temperature in vacuo, in air and under CO_2_ (Supplementary Fig. S6). The CO_2_ adsorption isotherms at elevated temperatures show no sign of flexibility or gate opening (Supplementary Fig. S11), nor does the CH_4_ isotherm at high pressure (Figure S15). In the specific case of C_2_H_4_ at 195 K, there is evidence of modest gate opening, which will be fully evaluated in future work (Figure S15).

**Fig. 6:**
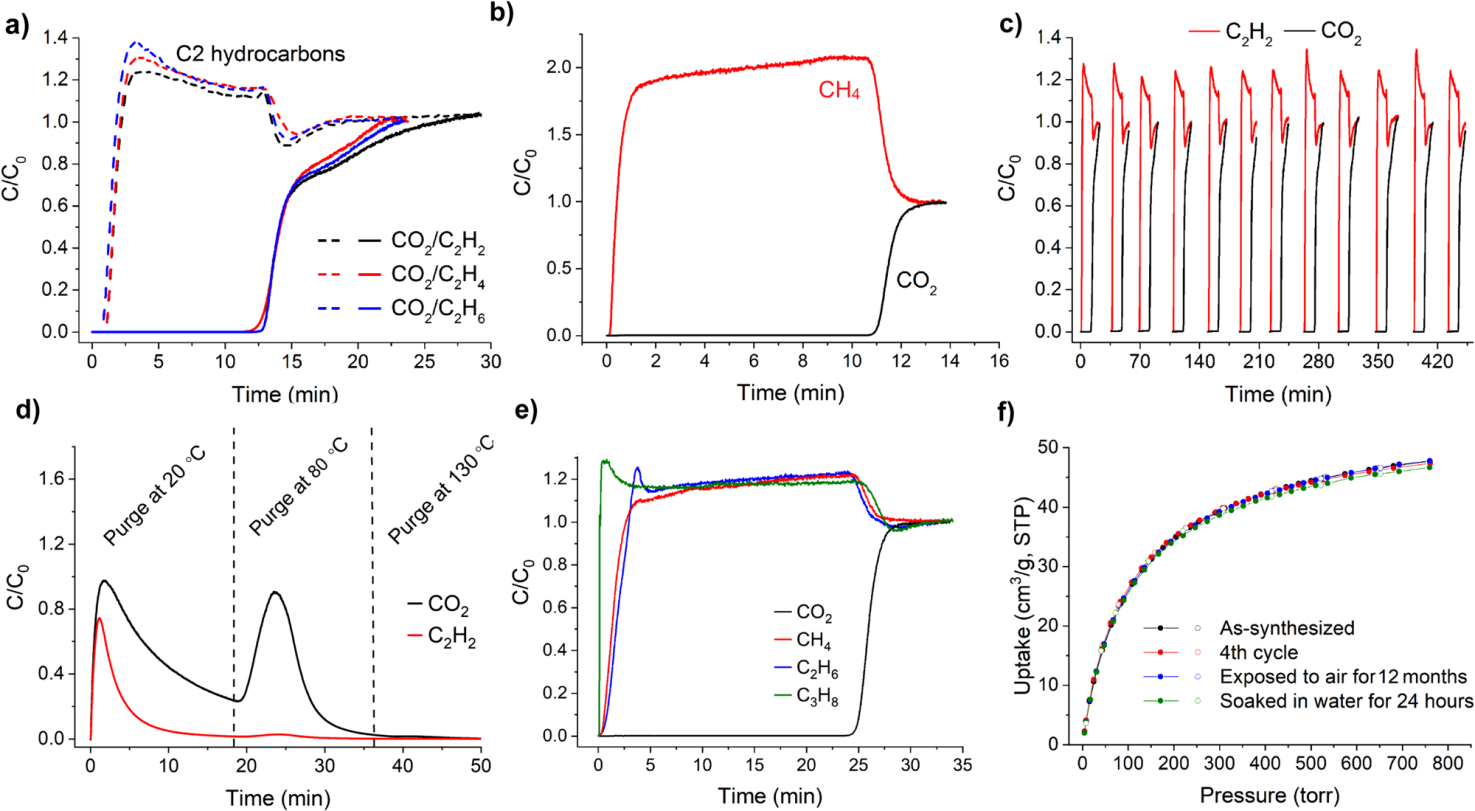
Gas separation by MUF-16. **a** Experimental breakthrough curves for 50/50 mixtures of CO_2_ and the three C2 hydrocarbons (measured independently) at 293 K and 1.1 bar in an adsorption column packed with MUF-16. **b** Experimental breakthrough curves for 50/50 mixtures of CO_2_ and CH_4_ at 293 K and 1.1 bar in an adsorption column packed with MUF-16. **c** Twelve separation cycles for a CO_2_/C_2_H_2_ mixture (50/50 mixture). Each separation process was carried out at 293 K and 1.1 bar. MUF-16 was regenerated between cycles by placing it under vacuum at ambient temperature for 20–25 min. **d** Experimental desorption profile of MUF-16 following the separation of CO_2_ and C_2_H_2_ upon heating under a helium flow of 5 ml_N_/min at 1.1 bar. No adsorbates were removed upon further heating at 130 °C indicating that they had been fully expelled at lower temperatures. **e** Experimental breakthrough curves for a 15/80/4/1 CO_2_/CH_4_/C_2_H_6_/C_3_H_8_ mixture at 1.1 bar and 293 K in an adsorption column packed with MUF-16. **f** CO_2_ adsorption isotherms (293 K) of as-synthesized MUF-16 after four consecutive adsorption-desorption cycles, after exposing it to air with ~80% humidity for 12 months, and after immersion in water for 48 hours. Source data are provided as a Source Data file.

Invigorated by these results, we then investigated the feasibility of CO_2_/hydrocarbon separations under dynamic conditions. Experimental breakthrough curves were measured for various gas mixtures at 293 K and 1.1 bar: CO_2_/C_2_H_6_ (50/50), CO_2_/C_2_H_4_ (50/50), CO_2_/C_2_H_2_ (50/50 and 5/95) and CO_2_/CH_4_ (50/50 and 15/85) (Fig. 6a, b; Supplementary Figs. S44 and S51). Figure 6a, b shows the dimensionless concentration of CO_2_ and the hydrocarbons (measured independently) exiting an adsorbent bed packed with MUF-16 (0.9 gram) as a function of time.

Complete separation was realised by MUF-16, whereby the hydrocarbons broke through from the column at an early stage because of their low affinity for the framework. Conversely, the signal of CO_2_ was not detected for at least 10 minutes due to its adsorption by MUF-16. The dynamic adsorption capacity for CO_2_ fell in the range 1.2–1.5 mmol/g which is nearly identical to the equilibrium capacity at the relevant partial pressures of CO_2_ (Supplementary Table 7). Significant volumes of pure hydrocarbons can be obtained in this way. Productivity calculations showed 1 kg of MUF-16 produces 27 L of the hydrocarbons from an equimolar mixture with CO_2_ at 293 K and 1 bar. The ability of MUF-16 to selectively adsorb CO_2_ is an important advantage of this MOF as pure hydrocarbons can be produced directly in a single adsorption stage. In literature reports to date, the capture of CO_2_ over C2 hydrocarbons has so far largely been restricted to cryogenic temperatures and/or static conditions^52–55,57,61^. With respect to CO_2_/C_2_H_2_ mixtures at ambient temperatures, we are aware of only a few reported materials, CD-MOF-1^51^, CD-MOF-2^51^ SIFSIX-3-Ni^25^, and [Tm_2_(OH-bdc)_2_(μ_3_- OH)_2_(H_2_O)_2_]^58^ for which this inverse trapping of CO_2_ has been verified by experimental breakthrough measurements. Since these MOFs adsorb C_2_H_2_ (in addition to CO_2_) strongly at moderate pressures, their uptake ratios are modest. They are limited to very low partial pressures of CO_2_ and suffer from low productivity.

Subsequent tests revealed that MUF-16 maintains its CO_2_ uptake and the complete removal of CO_2_ over at least 12 separation cycles (Fig. 6c). MUF-16 was regenerated between cycles by placing it under vacuum or by purging with an inert gas (Fig. 6d). Virtually all of the adsorbed acetylene and around half of the CO_2_ can be removed from the bed by purging at room temperature. The remainder can be fully desorbed at 80 °C.

To investigate separations involving trace CO_2_, we simulated breakthrough curves of feed gases with low CO_2_ partial pressures. First, a mass transfer coefficient was empirically determined based on measured breakthrough results to produce a match between simulated and experimental breakthrough curves^26,62^. With this realistic mass transfer coefficient in hand, we predicted breakthrough curves using feeds containing 0.1% CO_2_ in C_2_H_2_ (Supplementary Fig. S57). These calculations revealed that MUF-16 can eliminate trace quantities of CO_2_, as often required in industrial processes.

We then turned our attention to the separation of more complex gas mixtures. MUF-16 captures the CO_2_ from CO_2_/CH_4_/C_2_H_6_/C_3_H_8_ (15/80/4/1) feed mixtures at 1.1 bar. Here, we observed CH_4_, C_2_H_6_ and C_3_H_8_ to break through quickly with steep elution profiles (Fig. 6e). Crucially, the larger C_2_H_6_ and C_3_H_8_ components do not diminish the CO_2_ capture capabilities of MUF-16. This is an important observation for the removal of CO_2_ from natural gas, where mixed-gas separations involving these hydrocarbons are often required yet the pool of competent materials is limited^19,63^. To further probe the applicability of MUF-16 to natural gas sweeting, we conducted breakthrough measurements at a higher pressure of 9 bar. CO_2_ was cleanly removed from the gas stream (Supplementary Figs. S45 and S46). Breakthrough simulations at pressures relevant to natural gas processing (50 bar) lead to the prediction that MUF-16 can capture CO_2_ from natural gas (Supplementary Fig. S50). Water vapour is a component of crude natural gas streams and it can affect gas adsorption by physisorbents^64,65^. To test the moisture resistance of MUF-16, we measured its CO_2_ adsorption properties after exposure to air and immersion in water (Fig. 6f). The framework retains its CO_2_ adsorption capacity following these mistreatments. More detailed analysis, including the impact of water vapour on gas separation and the resistance of MUF-16 to other common natural gas impurities such as H_2_S, is an important next step.

In summary, the pores in MUF-16 are complementary to CO_2_ in size and electrostatic potential. This allows H-bonding and other noncovalent interactions to trap the guest CO_2_. Other guests, specifically methane and the C_2_ hydrocarbons, do not bind efficiently. This arises from the reversed polarity of these guests with respect to CO_2_ and results in a strong preference for CO_2_ over methane and inverted selectivity for CO_2_ over C_2_ and C_3_ hydrocarbon guests. MUF-16 shows exceptional performance for CO_2_/CH_4_ and CO_2_/C_2_H_2_ separations across a range of CO_2_/hydrocarbon compositions and pressures. These observations are relevant to the practical challenges of purifying natural gas and industrial feedstocks. MUF-16 has the potential to be produced economically on large scales and its chemical stability and recyclability meet the demands of a long-lived physisorbent. Given these characteristics, MUF-16 is a promising physisorbent for the capture of CO_2_.

## Supplementary information

Supplementary Information

Peer Review File

## Data Availability

Source data are provided with this paper. Crystallographic data and files of MUF-16 as synthesized, under vacuum and loaded with CO_2_ have been deposited (CCDC 1948901 - 1948905). Additional graphics, TG curves, PXRD diffractograms, multiple cycle adsorption isotherms, dual site Langmuir isotherm model fitting, isosteric heat of adsorption calculations, BET surface area calculations, IAST calculations of adsorption selectivities, breakthrough curves simulations and models used and column breakthrough test setup with procedures and measurements, and the DFT results are available as Supplementary Information. Further data that support the findings of this study are available from the corresponding author upon reasonable request. Source data are provided with this paper.

## Source data

Source Data

## References

[1] Sholl DS, Lively RP (2016). Seven chemical separations: to change the world: purifying mixtures without using heat would lower global energy use, emissions and pollution–and open up new routes to resources. Nature.

[2] Ravanchi MT, Sahebdelfar S (2014). Carbon dioxide capture and utilization in petrochemical industry: potentials and challenges. Appl. Petrochemical Res..

[3] Rochelle GT (2009). Amine scrubbing for CO_2_ capture. Science.

[4] Yu C-H, Huang C-H, Tan C-S (2012). A review of CO_2_ capture by absorption and adsorption. Aerosol Air Qual. Res..

[5] Kohl, A. & Nielsen, R. *Gas Purification*. 5th ed. (Gulf Publishing Company, Houston, 1997).

[6] Oschatz M, Antonietti M (2018). A search for selectivity to enable CO_2_ capture with porous adsorbents. Energ. Environ. Sci..

[7] Sreenivasulu B, Sreedhar I, Suresh P, Raghavan KV (2015). Development trends in porous adsorbents for carbon capture. Environ. Sci. Technol..

[8] Yang, R. T. *Gas separation by adsorption processes*. (Butterworth-Heinemann, 2013).

[9] Lu, A.-H. & Dai, S. *Porous materials for carbon dioxide capture*. (Springer, 2014).

[10] Lin R-B, Xiang S, Xing H, Zhou W, Chen B (2019). Exploration of porous metal–organic frameworks for gas separation and purification. Coord. Chem. Rev..

[11] Li H (2019). Porous metal-organic frameworks for gas storage and separation: status and challenges. EnergyChem.

[12] Ding M, Flaig RW, Jiang H-L, Yaghi OM (2019). Carbon capture and conversion using metal–organic frameworks and MOF-based materials. Chem. Soc. Rev..

[13] Mukherjee, S., Kumar, A. & Zaworotko, M. J. 2 - Metal-organic framework based carbon capture and purification technologies for clean environment. In *Metal-Organic Frameworks (MOFs) for Environmental Applications* (Ed. Ghosh, S. K.) 5–61 (Elsevier, 2019).

[14] Qazvini OT, Telfer SG (2020). A robust metal–organic framework for post-combustion carbon dioxide capture. J. Mater. Chem. A.

[15] Qazvini OT, Macreadie LK, Telfer SG (2020). Effect of ligand functionalization on the separation of small hydrocarbons and CO_2_ by a series of MUF-15 analogues. Chem. Mater..

[16] Patil KM, Telfer SG, Moratti SC, Qazvini OT, Hanton LR (2017). Non-interpenetrated Cu-based MOF constructed from a rediscovered tetrahedral ligand. CrystEngComm.

[17] Balashankar VS, Rajagopalan AK, Pauw RD, Avila AM, Rajendran A (2019). Analysis of a batch adsorber analogue for rapid screening of adsorbents for postcombustion CO_2_ capture. Ind. Eng. Chem. Res..

[18] Rajagopalan AK, Avila AM, Rajendran A (2016). Do adsorbent screening metrics predict process performance? A process optimisation based study for post-combustion capture of CO_2_. Int. J. Greenh. Gas. Con..

[19] Rufford TE (2012). The removal of CO_2_ and N_2_ from natural gas: a review of conventional and emerging process technologies. J. Petrol. Sci. Eng..

[20] Madden DG (2019). Highly selective CO_2_ removal for one-step liquefied natural gas processing by physisorbents. Chem. Commun..

[21] Belmabkhout Y (2018). Natural gas upgrading using a fluorinated MOF with tuned H_2_S and CO_2_ adsorption selectivity. Nat. Energy.

[22] Matar, S. & Hatch, L. F. *Chemistry of Petrochemical Processes*. (Gulf Professional Publishing, 2001).

[23] Hort, E. V. & Taylor, P. Acetylene‐Derived Chemicals. *Kirk‐Othmer Encyclopedia of Chemical Technology* (2000).

[24] Pässler P (2008). Acetylene. Ullmann’s Encycl. Ind. Chem..

[25] Chen K-J (2016). Benchmark C_2_H_2_/CO_2_ and CO_2_/C_2_H_2_ separation by two closely related hybrid ultramicroporous materials. Chem.

[26] Qazvini OT (2019). Ethane-trapping metal–organic framework with a high capacity for ethylene purification. J. Am. Chem. Soc..

[27] Qazvini OT, Babarao R, Telfer SG (2019). Multipurpose metal–organic framework for the adsorption of acetylene: ethylene purification and carbon dioxide removal. Chem. Mater..

[28] Tang E (2006). Two cobalt(II) 5-aminoisophthalate complexes and their stable supramolecular microporous frameworks. Inorg. Chem..

[29] Tian C-B (2015). Four new MnII inorganic–organic hybrid frameworks with diverse inorganic magnetic chain’s sequences: syntheses, structures, magnetic, NLO, and dielectric properties. Inorg. Chem..

[30] Zhai Q-G, Bu X, Zhao X, Li D-S, Feng P (2017). Pore space partition in metal–organic frameworks. Acc. Chem. Res..

[31] Sumida K (2012). Carbon dioxide capture in metal–organic frameworks. Chem. Rev..

[32] Wong-Ng, W. In situ diffraction studies of selected metal–organic framework materials for guest capture. In *Materials and Processes for CO*_*2*_*Capture, Conversion, and Sequestration* (Eds. Li, L., Wong-Ng, W., Huang, K. & Cook, L. P.) (2018).

[33] Easun TL, Moreau F, Yan Y, Yang S, Schröder M (2017). Structural and dynamic studies of substrate binding in porous metal–organic frameworks. Chem. Soc. Rev..

[34] Hou L (2011). A rod packing microporous metal–organic framework: unprecedented ukv topology, high sorption selectivity and affinity for CO_2_. Chem. Commun..

[35] Jiang M (2018). Controlling pore shape and size of interpenetrated anion-pillared ultramicroporous materials enables molecular sieving of CO_2_ combined with ultrahigh uptake capacity. ACS Appl. Mater. Interfaces.

[36] Moreau F (2017). Unravelling exceptional acetylene and carbon dioxide adsorption within a tetra-amide functionalized metal-organic framework. Nat. Commun..

[37] Xiang S, Zhou W, Gallegos JM, Liu Y, Chen B (2009). Exceptionally high acetylene uptake in a microporous metal−organic framework with open metal sites. J. Am. Chem. Soc..

[38] Li P (2015). A rod-packing microporous hydrogen-bonded organic framework for highly selective separation of C_2_H_2_/CO_2_ at room temperature. Angew. Chem., Int. Ed..

[39] Lee J (2018). Separation of acetylene from carbon dioxide and ethylene by a water-stable microporous metal–organic framework with aligned imidazolium groups inside the channels. Angew. Chem. Int. Ed..

[40] Luo F (2016). UTSA-74: A MOF-74 isomer with two accessible binding sites per metal center for highly selective gas separation. J. Am. Chem. Soc..

[41] Zhang J-P, Chen X-M (2009). Optimized acetylene/carbon dioxide sorption in a dynamic porous crystal. J. Am. Chem. Soc..

[42] Zhang L (2018). Efficient separation of C_2_H_2_ from C_2_H_2_/CO_2_ mixtures in an acid–base resistant metal–organic framework. Chem. Commun..

[43] Peng Y-L (2018). Robust ultramicroporous metal–organic frameworks with benchmark affinity for acetylene. Angew. Chem., Int. Ed..

[44] Scott HS (2017). Highly selective separation of C_2_H_2_ from CO_2_ by a new dichromate-based hybrid ultramicroporous material. ACS Appl. Mater. Interfaces.

[45] Matsuda R (2005). Highly controlled acetylene accommodation in a metal–organic microporous material. Nature.

[46] Duan X (2016). A novel metal-organic framework for high storage and separation of acetylene at room temperature. J. Solid State Chem..

[47] Duan X (2014). A new metal–organic framework with potential for adsorptive separation of methane from carbon dioxide, acetylene, ethylene, and ethane established by simulated breakthrough experiments. J. Mat. Chem. A.

[48] Gao J (2020). Mixed metal–organic framework with multiple binding sites for efficient C_2_H_2_/CO_2_ separation. Angew. Chem. Int. Ed..

[49] Ye Y (2019). Pore space partition within a metal–organic framework for highly efficient C_2_H_2_/CO_2_ separation. J. Am. Chem. Soc..

[50] Fan W (2020). Optimizing multivariate metal–organic frameworks for efficient C_2_H_2_/CO_2_ separation. J. Am. Chem. Soc..

[51] Li L (2019). Inverse adsorption separation of CO_2_/C_2_H_2_ mixture in cyclodextrin-based metal–organic frameworks. ACS Appl. Mater. Interfaces.

[52] Foo ML (2016). An adsorbate discriminatory gate effect in a flexible porous coordination polymer for selective adsorption of CO_2_ over C_2_H_2_. J. Am. Chem. Soc..

[53] Duan J (2013). A family of rare Earth porous coordination polymers with different flexibility for CO_2_/C_2_H_4_ and CO_2_/C_2_H_6_ separation. Inorg. Chem..

[54] Yang W (2012). Selective CO_2_ uptake and inverse CO_2_/C_2_H_2_ selectivity in a dynamic bifunctional metal–organic framework. Chem. Sci..

[55] Eguchi R, Uchida S, Mizuno N (2012). Inverse and high CO_2_/C_2_H_2_ sorption selectivity in flexible organic–inorganic ionic crystals. Angew. Chem., Int. Ed..

[56] Horike S (2012). Dense coordination network capable of selective CO_2_ capture from C1 and C2 hydrocarbons. J. Am. Chem. Soc..

[57] Noro S-i (2009). Selective gas adsorption in one-dimensional, flexible CuII coordination polymers with polar units. Chem. Mater..

[58] Ma D (2020). Inverse and highly selective separation of CO_2_/C_2_H_2_ on a thulium–organic framework. J. Mater. Chem. A.

[59] Myers A, Prausnitz JM (1965). Thermodynamics of mixed‐gas adsorption. AIChE J..

[60] Subraveti SG (2019). Cycle design and optimization of pressure swing adsorption cycles for pre-combustion CO_2_ capture. Appl. Energy.

[61] Yanai N (2011). Gas detection by structural variations of fluorescent guest molecules in a flexible porous coordination polymer. Nat. Mater..

[62] Qazvini OT, Fatemi S (2015). Modeling and simulation pressure–temperature swing adsorption process to remove mercaptan from humid natural gas; a commercial case study. Sep. Purif. Technol..

[63] Krishna R (2020). Metrics for evaluation and screening of metal–organic frameworks for applications in mixture separations. ACS Omega.

[64] Mukherjee S (2019). Trace CO_2_ capture by an ultramicroporous physisorbent with low water affinity. Sci. Adv..

[65] Masala A (2017). CO_2_ capture in dry and wet conditions in UTSA-16 metal–organic framework. ACS Appl. Mater. Interfaces.

