# Supplementary material for: Selective capture of carbon dioxide from hydrocarbons using a metal-organic framework
Source: Nat Commun. 2021 Jan 8;12:197. doi: 10.1038/s41467-020-20489-2 (PMC7794324; doi:10.1038/s41467-020-20489-2)
Supplement: Supplementary file 1 — Supplementary Information [file 41467_2020_20489_MOESM1_ESM.pdf]

Supplementary Information for

Selective Capture of Carbon Dioxide from Hydrocarbons Using a Metal-Organic Framework

Omid T. Qazvini[1,2], Ravichandar Babarao[3,4], and Shane G. Telfer[*,1]

[1]MacDiarmid Institute for Advanced Materials and Nanotechnology, School of Fundamental Sciences, Massey University, Palmerston North, New Zealand.
[2]Department of Chemical Engineering and Analytical Science, The University of Manchester, Oxford Road, Manchester M13 9PL, UK.
[3]School of Science, RMIT University, Melbourne, Victoria 3001, Australia.
[4]Commonwealth Scientific and Industrial Research Organisation (CSIRO) Manufacturing, Clayton, Victoria 3169, Australia.

Contents

1. Supplementary Notes: General procedure and information....2
2. Supplementary Methods: Synthesis details....2
2.1 MUF-16 ([Co(Haip)$_2$])....2
2.2 MUF-16(Mn) and MUF-16(Ni) ([Mn(Haip)$_2$] and [Ni(Haip)$_2$])....2
2.3 Elemental analyses of the MUF-16 frameworks....3
3. Supplementary Methods: Thermogravimetric Analysis (TGA)....4
4. Supplementary Methods: Single crystal X-ray diffraction....5
4.1 As-synthesized MUF-16, MUF-16(Ni) and MUF-16(Mn)....5
4.2 Single crystal X-ray crystallography under vacuum and loaded with CO_2....7
4.3 Refinement details for guest-free and CO_2-loaded MUF-16(Mn)....8
5. Supplementary Methods: Powder X-ray diffraction patterns....10
6. Supplementary Notes: Textural properties and gas adsorption measurements....13
7. Supplementary Notes: Calculation of BET surface areas....20
8. Supplementary Notes: Heat of adsorption....22
9. Supplementary Notes: IAST calculations....24
10. Supplementary Methods: Breakthrough separation experiments and simulations....35
10.1. CO_2/CH_4 and $CO_2/CH_4+C_2H_6+C_3H_8$ breakthrough separations....37
10.1.1. Simulations of CO_2/CH_4 breakthrough curves....39
10.2 CO_2/C2 hydrocarbon separations....41
10.2.1. Simulations of CO_2/C_2H_2 breakthrough curves....44
11. Supplementary Tables: Reported separation metrics....46
12. Supplementary Methods: DFT calculations....50
13. Supplementary References....51

1. Supplementary Notes: General procedure and information

All starting compounds and solvents were used as received from commercial sources without further purification unless otherwise noted. Elemental analyses were performed by the Campbell Microanalytical Laboratory at the University of Otago, New Zealand.

2. Supplementary Methods: Synthesis details

2.1 MUF-16 ([Co(Haip)$_2$])

Small-scale synthesis:

A mixture of $Co(OAc)_2 \cdot 4H_2O$ (0.625 g, 2.5 mmol), 5-aminoisophthalic acid (1.8 g, 10 mmol), methanol (80 mL) and water (5 ml) were sonicated for 20 min in a sealed 1000 mL Schott bottle, which was then heated in a pre-heated oven at 85 °C for 2 hours under autogenous pressure. After cooling the oven to room temperature, the resulting pink crystals were isolated by decanting off the mother liquor, washed with methanol several times and dried under vacuum at 130 °C for 20 h. Yield: 0.98 g (94% based on cobalt) of guest-free MUF-16.

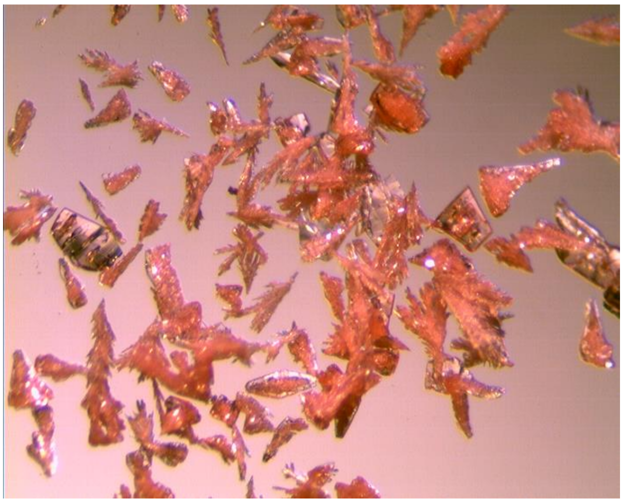

Supplementary Figure 1. Crystal of MUF-16.

2.2 MUF-16(Mn) and MUF-16(Ni) ([Mn(Haip)$_2$] and [Ni(Haip)$_2$])

A mixture of $M(ClO_4)_2 \cdot 6H_2O$ (where M = Mn or Ni) (1.25 mmol), 5-aminoisophthalic acid (2.50 mmol, 0.45 g), and NH_4NO_3 (2.50 mmol, 0.20 g) with a mixed-solvent of CH_3CN (20 mL) and CH_3OH (15 mL) were sonicated for 20 min and sealed in a 100 mL Teflon-lined stainless-steel reaction vessel and heated at 160 °C for two days under autogenous pressure. After cooling the oven to room temperature, the resulting brownish-coloured crystals were isolated by decanting off the mother liquor, washed with

methanol several times (until the filtrate ran colourless) then dried under vacuum at 130 °C for 20 h. Yields: 0.21 g (40% based on Mn) of guest free MUF-16(Mn), and 0.28 g (53% based on Ni) of guest-free MUF-16(Ni).

2.3 Elemental analyses of the MUF-16 frameworks.

	C: calcd./found	H: calcd./found	N: calcd./found
MUF-16·H_2O	43.95/43.49	3.23/3.23	6.41/6.40
MUF-16(Mn)·H_2O	44.36/44.05	3.26/3.42	6.47/6.64
MUF-16(Ni)·H_2O	43.98/44.18	3.23/3.57	6.41/6.90

3. Supplementary Methods: Thermogravimetric Analysis (TGA)

Freshly prepared MOF samples were washed with MeOH, and then activated at 130 °C under vacuum for 10 hours. Samples were exposed to air for one hour and then transferred to an aluminium sample pan. Measurements were then commenced under an N_2 flow with a heating rate of 5 °C /min.

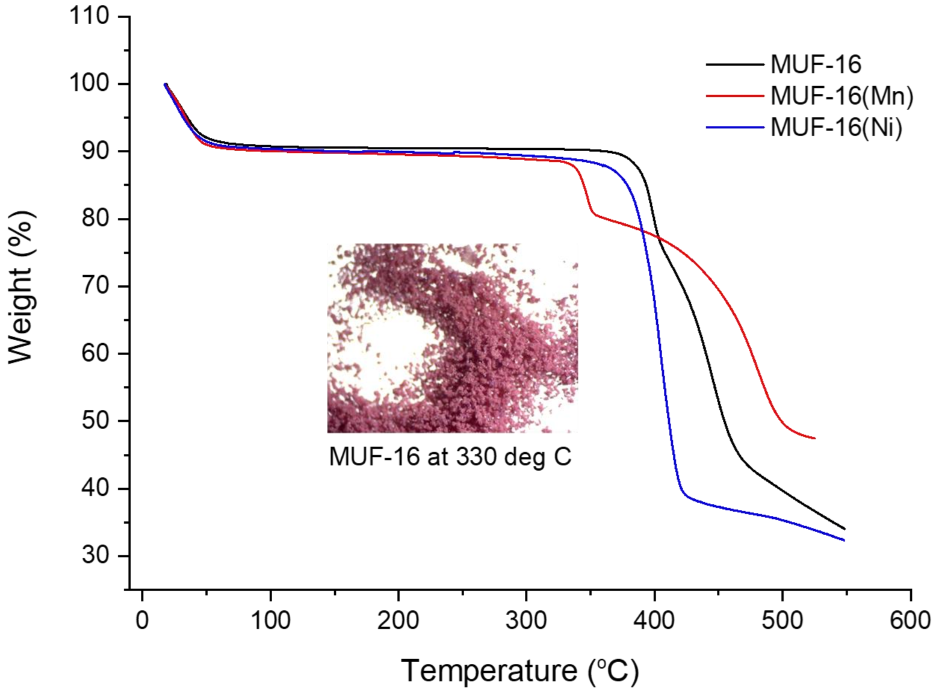

Supplementary Figure 2. TGA curves of MUF-16, MUF-16(Mn), and MUF-16(Ni) under N_2. The inset shows an optical micrograph of MUF-16 crystals after heating to 330 °C. The PXRD pattern of this material is shown below. Source data are provided as a Source Data file.

4. Supplementary Methods: Single crystal X-ray diffraction

A Rigaku Spider diffractometer equipped with a MicroMax MM007 rotating anode generator (Cu_{α} radiation, 1.54180 Å), high-flux Osmic multilayer mirror optics, and a curved image plate detector was used to collect SCXRD data.

4.1 As-synthesized MUF-16, MUF-16(Ni) and MUF-16(Mn)

General

MOF crystals were analysed after removing them from methanol. Room temperature data collections produced better refinement statistics than low temperature data collections. All atoms were found in the electron density difference map. All atoms were refined anisotropically, except hydrogen atoms and certain of the water molecules in the pores (as specified below). The structures of solvated MUF-16[1] and MUF-16(Mn)[2] have been reported previously.

MUF-16

O15 of an occluded H_2O molecule was refined isotropically. It does not act as a H-bond donor. Despite numerous data collections, the wR_2 value remained high due to an inherent lack of precise ordering in the material. A small (1.95 eA^{-3}) electron density peak remained near the Co site due to Fourier series truncation ripples.

MUF-16(Ni)

The crystals diffracted to a resolution of just 1.0 Å thus the calculated $\sin(\theta_{max})$/wavelength is 0.4999. This limited the number of data and produced a relatively low data: parameter ratio (7.3) and low precision on the C-C bonds. Despite numerous data collections, the wR_2 value remained high due to an inherent lack of precise ordering in the material. A small (1.55 eA^{-3}) electron density peak remained near the Ni site due to Fourier series truncation ripples. Occluded water molecule O16 does not act as a H-bond donor. A SHEL command (SHEL 8 1) was used to limit the data used in the refinement to values that were sensibly measured.

A solvent mask was calculated and 124 electrons were found in a volume of 308 $Å^3$ in one void per unit cell. This is consistent with the presence of three disordered water molecules per asymmetric unit, which account for 120 electrons per unit cell.

Supplementary Table 1. Crystal data and structure refinement details for MUF-16, MUF-16(Mn) and MUF-16(Ni).

	**MUF-16**	**MUF-16(Mn)**	**MUF-16(Ni)**
Formula	$Co(Haip)_2{\cdot}2H_2O$	$Mn(Haip)_2{\cdot}3H_2O$	$Ni(Haip)_2{\cdot}3H_2O$
CCDC deposition no.	1948901	1948902	1948903
Empirical formula	$C_{16}H_{16}CoN_2O_{10}$	$C_{16}H_{18}MnN_2O_{11}$	$C_{16}H_{18}N_2NiO_{11}$
Formula weight	455.24	471.28	473.3
Temperature / K	292	292	293.0
Crystal system	monoclinic	monoclinic	monoclinic
Space group	*I*2/a	*I*2/*a*	*I*2/a
a / Å	15.3514(15)	25.2367(14)	15.4963(11)
b / Å	4.4232(4)	4.57990(10)	4.5780(2)
c / Å	25.614(4)	15.4895(11)	25.230(2)
α / °	90	90	90
β / °	94.294(10)	96.046(8)	96.177(8)
γ / °	90	90	90
Volume / $Å^3$	1734.4(4)	1780.34(17)	1779.5(2)
Z	4	4	4
ρ_{calc} / g cm^{-3}	1.743	1.758	1.564
μ / mm^{-1}	8.357	6.682	2.020
F(000)	932.0	972.0	856.0
Resolution range for data/ Å	0.81	0.81	1.0
Reflections collected	7472	14132	6610
Independent reflections	1594 [R_{int} = 0.0918, R_{sigma} = 0.0917]	1668 [R_{int} = 0.1054, R_{sigma} = 0.1158]	925 [R_{int} = 0.0917, R_{sigma} = 0.0852]
Data/restraints/parameters	1594/2/136	1668/1/149	925/0/126
Goodness-of-fit on F2	1.301	1.152	1.649
Final R indices [I>2σ(I)]	R_1 = 0.1185, wR_2 = 0.3035	R_1 = 0.0740, wR_2 = 0.1821	R_1 = 0.1517, wR_2 = 0.3672
Final R indices [all data]	R_1 = 0.1576, wR_2 = 0.3785	R_1 = 0.1350, wR_2 = 0.2421	R_1 = 0.2061, wR_2 = 0.4467
Largest diff. peak/hole / e $Å^{-3}$	0.93/-1.26	0.57/-0.51	0.77/-0.83

4.2 Single crystal X-ray crystallography under vacuum and loaded with CO_2

Capillary SCXRD was performed for a single crystal of MUF-16(Mn) both under vacuum and loaded with CO_2 at around 1.1 bar and 20 °C based on the following steps:
First a single crystal was chosen with an appropriate size (~ 0.1 × 0.1 × 0.2 mm) and soaked in ethanol. A small capillary tube with around 0.2 mm in diameter and 50 mm in length (which is open at both ends) was made by burning and shaping the neck of a glass pipette (referred to as the 'home-made capillary'). The home-made capillary was then used to trap the crystal inside it. Normally, the crystal flowed through the capillary carried by the ethanol stream.

The home-made capillary was then transferred into a standard 0.3 mm capillary. A long, thin device was then used to push the home-made capillary to the very bottom of the 0.3 mm capillary.

Around 6 or 7 crystals of cobalt chloride hydrate were then transferred to the 0.3 mm capillary and placed on the top of the home-made capillary. The cobalt chloride was used a visual indicator of the level of water vapour in the capillary based on its pink → blue colour change upon dehydration.

The top of the 0.3 mm capillary was then covered by glass wool to avoid the elutriation of cobalt chloride crystals during activation.

The capillary assembly was then connected to an adsorption apparatus (Quantachrome-Autosorb-iQ2) using appropriate Swagelok fittings (Supplementary Figure 3) and was kept under vacuum and a temperature of 140 °C for around 5 hours so that the vacuum level reached 0.0008 torr. At this point the cobalt chloride crystals were blue in colour (indicating an anhydrous environment).

The capillary was flame sealed to trap the crystal under vacuum. Alternatively, the capillary was filled with CO_2 to a pressure of 1.2 bar and then flame sealed.

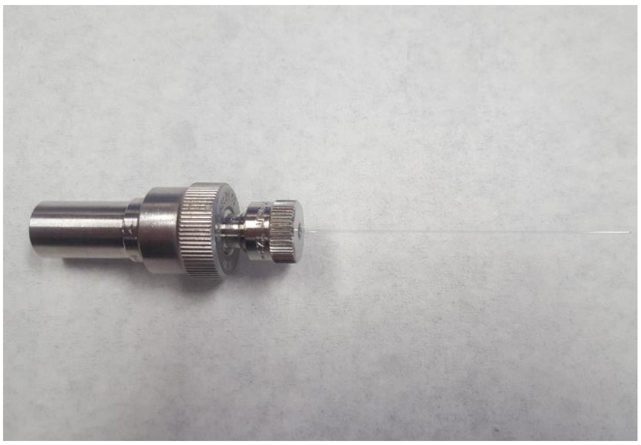

Supplementary Figure 3. Swagelok fittings for connecting a capillary to Quantachrome-Autosorb-iQ2.

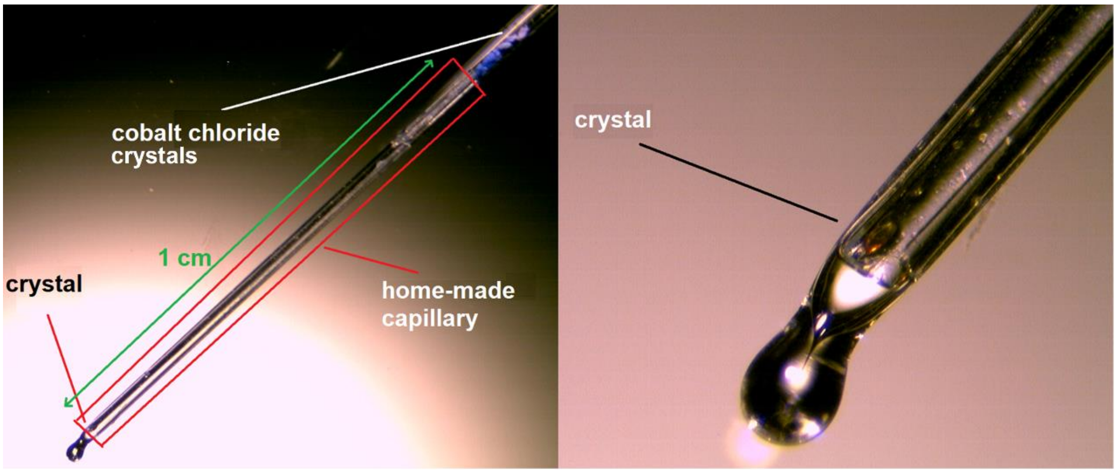

Supplementary Figure 4. Schematic and dimensions of capillaries used for SCXRD.

4.3 Refinement details for guest-free and CO_2-loaded MUF-16(Mn)

General

Certain reflections were omitted from the refinement process since they were mismeasured due to the presence of the glass capillary. All non-hydrogen atoms were found in the Fourier difference map.

MUF-16(Mn) in vacuo

The crystals diffracted to a resolution of just 0.90 Å thus the calculated sin(θ_{max})/wavelength is 0.555.

MUF-16(Mn) under CO_2

The crystals diffracted to a resolution of just 1.08 Å thus the calculated sin(θ_{max})/wavelength is 0.463. This limited the number of data and produced a relatively low data: parameter ratio (5.3) and low precision on the C-C bonds. A SHEL command (SHEL 8 1.08) was used to define the data range for refinement.

A strong electron density peak was observed in the middle of the pore and two weaker areas of electron density towards the pore surface. The central dense area was assigned to be an oxygen (O15) with a while the other two areas were ascribed to oxygen (O16) and carbon (C17) atoms. The C=O bond lengths were restrained to 1.16 Å and the O=C=O angle to 180°. The C and O atoms were refined isotropically. This describes two disordered CO_2 molecules that each occupy one of two sites. The two molecules share the O15 atom. Overall, this allows for up to CO_2 molecule per Mn centre. Refinement of the occupancy of the CO_2 guest converged on 0.77 molecules per Mn.

Supplementary Table 2. SCXRD data and refinement details of guest-free and CO_2-loaded MUF-16(Mn).

	**MUF-16(Mn)** ***in vacuo***	**MUF-16(Mn) under CO_2 (1.2 bar)**
Formula	Mn(Haip)$_2$	Mn(Haip)$_2$·0.77CO_2
CCDC deposition no.	1948905	1948904
Empirical formula	$C_{16}H_{12}MnN_2O_8$	$C_{16.77}H_{12}MnN_2O_{9.55}$
Formula weight	415.22	449.25
Temperature/K	292	292
Crystal system	monoclinic	monoclinic
Space group	*I*2/a	*I*2/a
a/Å	15.4872(11)	15.5719(10)
b/Å	4.51930(10)	4.52010(10)
c/Å	25.4913(13)	25.438(2)
α/°	90	90
β/°	97.080(16)	97.108(8)
γ/°	90	90
Volume/Å^3	1770.56(17)	1776.7(2)
Z	4	4
ρ_{calc} /g cm^{-3}	1.558	1.717
μ/mm^{-1}	6.512	6.611
F(000)	844.0	912.0
Data range for refinement/ Å	0.90	1.08
Reflections collected/ind.	7515/1214 [R_{int} = 0.1632, R_σ = 0.1964]	8177/713 [R_{int} = 0.1104, R_σ = 0.0804]
Data/restraints/parameters	1214/0/129	713/90/136
Goodness-of-fit on F^2	0.862	1.121
Final R indexes [I>=2σ (I)]	R_1 = 0.0510, wR_2 = 0.0954	R_1 = 0.0891, wR_2 = 0.2299
Final R indexes [all data]	R_1 = 0.1341, wR_2 = 0.1112	R_1 = 0.1299, wR_2 = 0.2944
Largest diff. peak/hole / e Å^{-3}	0.35/-0.48	0.65/-0.62

5. Supplementary Methods: Powder X-ray diffraction patterns

The data were obtained from freshly prepared MOF samples that had been washed several times with MeOH. MOF crystals were analysed right after removing them from MeOH. The two-dimensional images of the Debye rings were integrated to give 2θ vs I diffractograms. Predicted powder patterns were generated from single crystal structures using Mercury.

For aging experiments on the frameworks, after washing as-synthesized samples several times with MeOH, they were activated and were aged in air at 70-85% relative humidity or water at 20 °C.

To measure PXRD patterns under gas loading or *in vacuo*, a MOF sample was first loaded into a thin-walled glass capillary in a similar way to the SCXRD experiments. It was then activated under vacuum with mild heating before being flame-sealed directly or after back-filling with the selected gas.

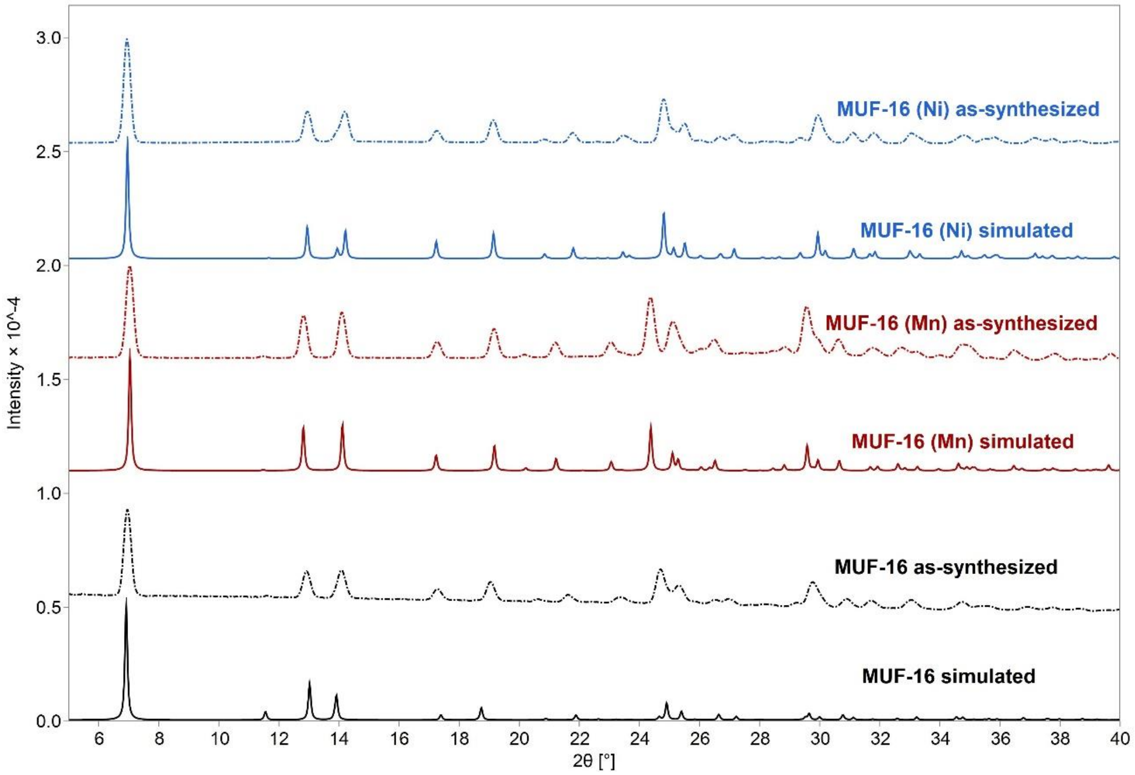

Supplementary Figure 5. PXRD patterns of MUF-16, MUF-16(Mn) and MUF-(Ni) with comparisons between measurements on as-synthesized bulk samples and diffractograms predicted from SCXRD structures.

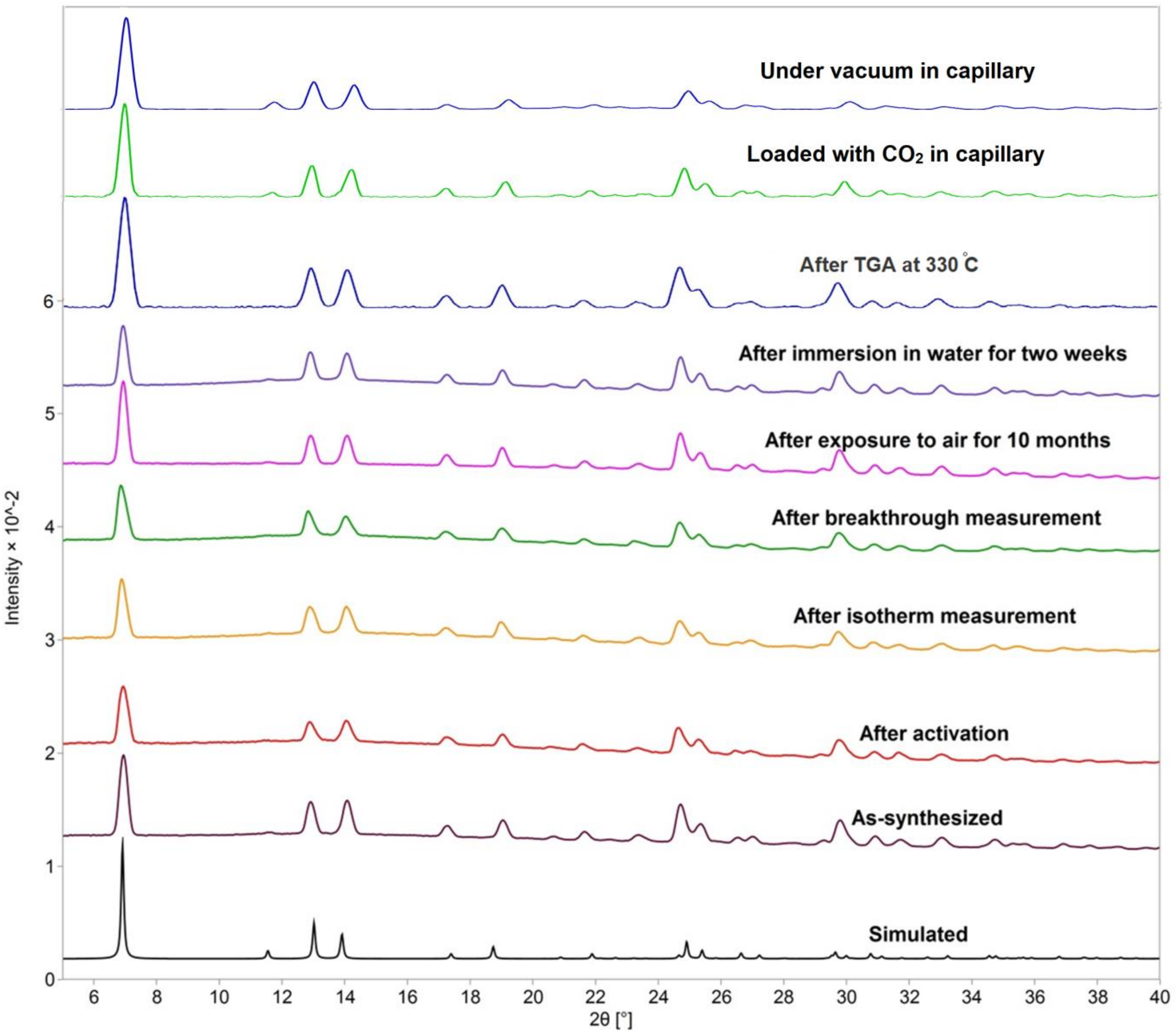

Supplementary Figure 6. PXRD patterns of MUF-16 showing that its structure remains unchanged after activation at 130 °C under vacuum, after isotherm measurements, after breakthrough experiments, after exposure to an air with relative humidity of >80% for at least 12 months, after immersion in water for two weeks, after heating to 330 °C (under a N_2 flow in a TGA), loaded with CO_2 in capillary, and under vacuum in capillary.

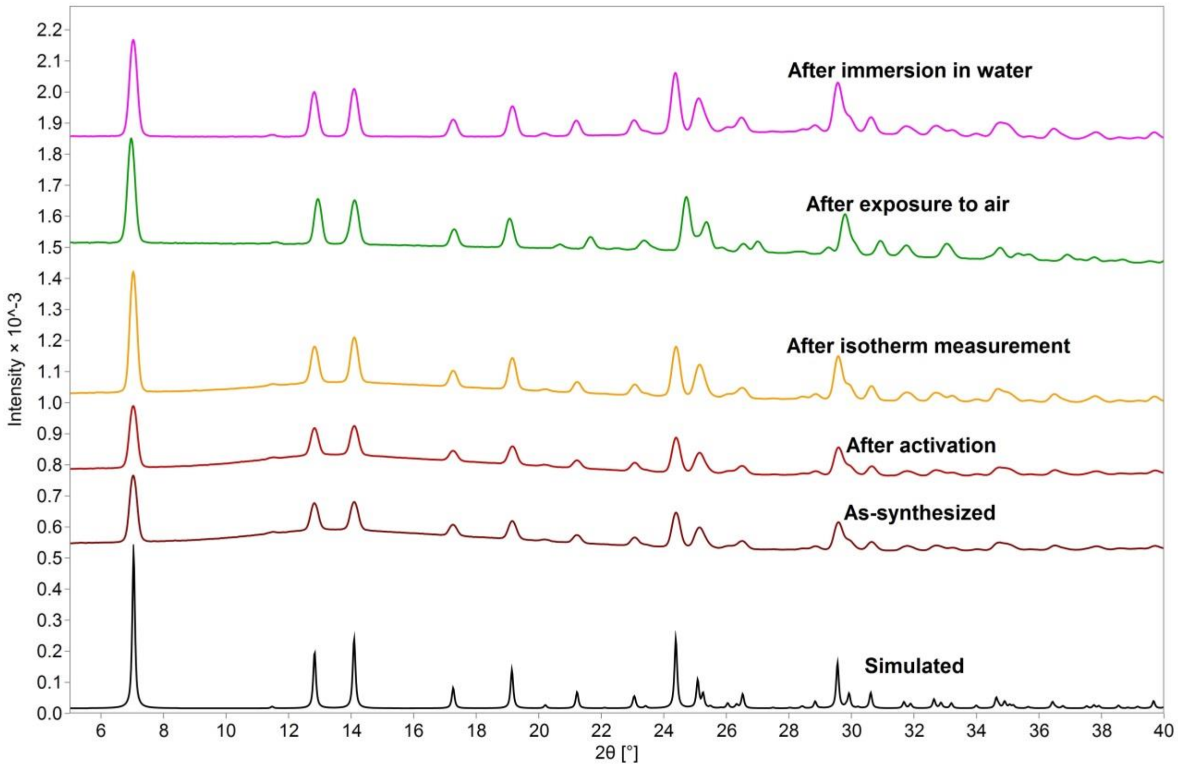

Supplementary Figure 7. PXRD patterns of MUF-16(Mn) showing that its structure remains unchanged after activation at 130 °C under vacuum, after isotherm measurements, after exposure to an air with relative humidity of >80% for at least 12 months and after immersion in water for 2 weeks.

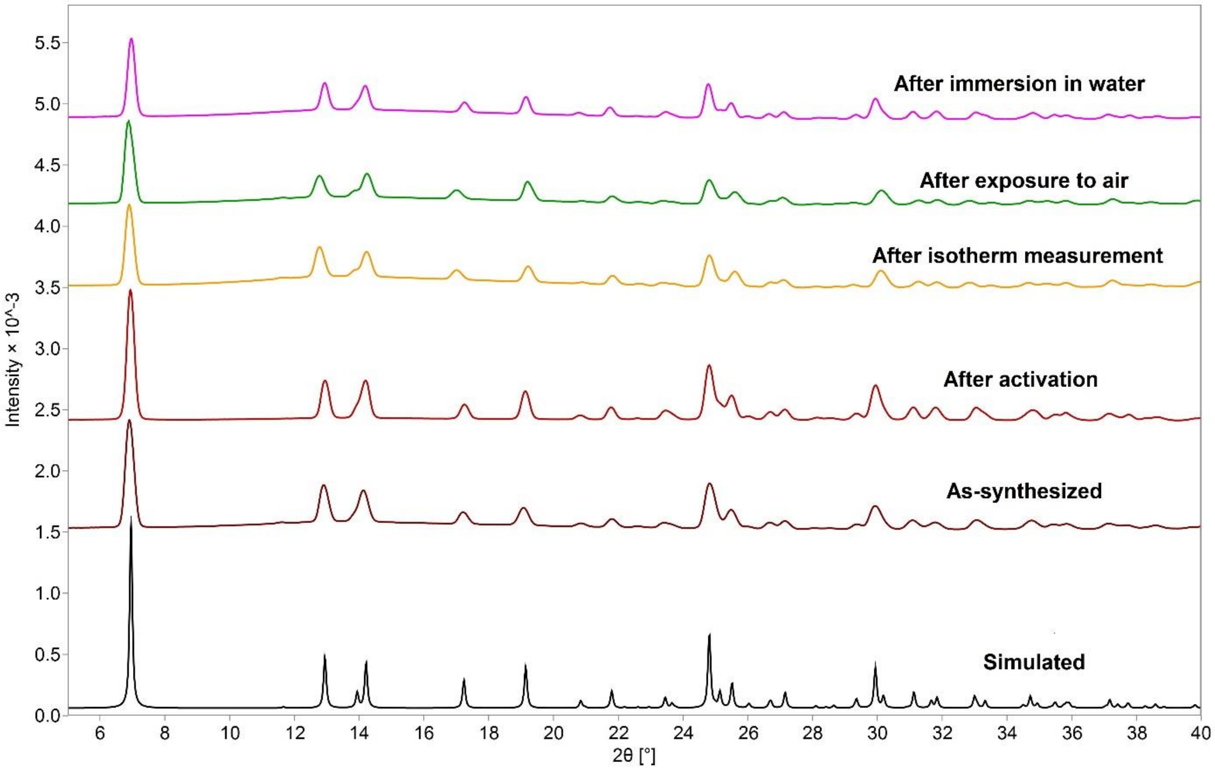

Supplementary Figure 8. PXRD patterns of MUF-16(Ni) showing that its structure remains unchanged after activation at 130 °C under vacuum, after isotherm measurements, after exposure to an air with relative humidity of >80% for at least 12 months and after immersion in water for 2 weeks.

6. Supplementary Notes: Textural properties and gas adsorption measurements

Single crystal structures of MUF-16, MUF-16(Mn) and MUF-16(Ni) were used directly for all the calculations and simulations without modification except removal of occluded solvent, where relevant. Low pressure adsorption isotherms were measured on a Quantachrome iQ2 instrument at Massey University, while high pressure measurements used a iSorbHP instrument at Quantachtome HQ in Florida, USA. The Zeo++[3] code and RASPA2[4] were used to calculate their pore volumes and surface areas with the use of H_2 and He probes, respectively.

Supplementary Table 3. Some calculated and experimentally determined properties of the MUF-16 family.

	MUF-16	MUF-16(Mn)	MUF-16(Ni)
Geometric surface area (m^2/g, Zeo++)	313	315	313
BET surface area (m^2/g, from experimental N_2 isotherm/77 K)	214	205	204
Calculated void fraction (%, RASPA2)	17.3	17.0	16.7
Calculated pore volume (cm^3/g, RASPA2)	0.10	0.11	0.11
Pore volume (cm^3/g, from experimental N_2 isotherm/77 K)	0.11	0.12	0.11

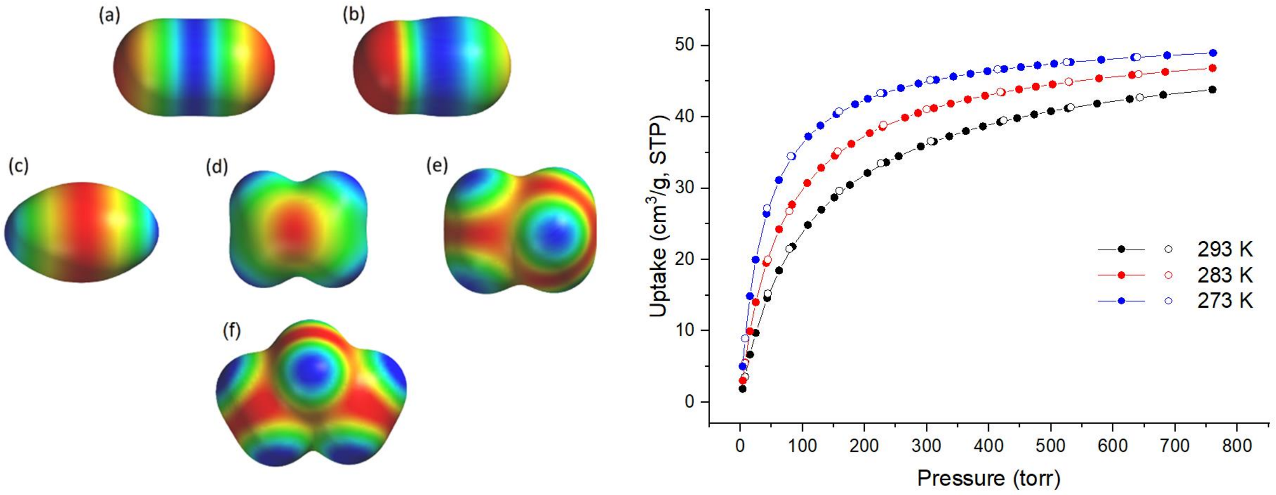

Supplementary Figure 9. Left: Electrostatic potential maps of (a) CO_2, (b) (N_2O) (c) C_2H_2, (d) C_2H_4, (e) C_2H_6 and (f) C_3H_8 Blue/green = positive; red/orange = negative; Right: Volumetric adsorption isotherms of N_2O measured at different temperatures for MUF-16.

Supplementary Table 4. Physicochemical characteristics of different gasses relevant to their separation.[5-8]

	Boiling point (K)	Molecular dimensions (Å)	Polarizability (Å^3)	Dipole moment ×10^{18}/esu cm^2	Quadrupole moment ×10^{26}/esu cm^2
CO_2	216.5	3.18×3.33×5.36	2.91	0	-4.3
CH_4	111.66	3.82×3.94×4.10	2.59	0	0
C_2H_2	188.4	3.32×3.34×5.7	3.33-3.93	0	+7.5
C_2H_4	169.4	3.28×4.18×4.84	4.25	0	+1.5
C_2H_6	184.5	3.81×4.82×4.08	4.43-4.47	0	+0.65
C_3H_8	231.0	6.80×4.20×3.80	6.29-6.37	0.084	-
C_3H_6	225.4	-	6.26	0.366	-

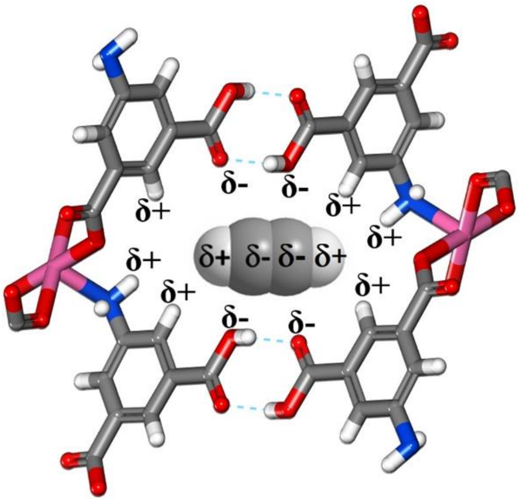

Supplementary Figure 10. Schematic showing that the electrostatic potential distribution on the pore surface of MUF-16 would lead to repulsive interactions with guest C_2H_2 molecules *if* they were to occupy the sites crystallographically observed for the binding of CO_2.

For adsorption measurements, as-synthesized samples were washed with anhydrous methanol several times and 50-1000 mg was transferred into a pre-dried and weighed sample tube. Large sample quantities (~1 g) were used to measure isotherms of the weakly-adsorbing gases to ensure that the recorded uptake measurements were reliable. To activate the sample, it was heated at rate of 10°C/min to a temperature of 130 °C under a dynamic vacuum with a turbomolecular pump for 20 hours.

Supplementary Table 5. Uptake capacity of MUF-16 for CO_2 at 293 K and 1 bar.

	**Uptake (wt%)**	**Molecules of CO_2 per unit cell**	**Molecules of CO_2 per metal**	**Fraction of void volume occupied by CO_2***
MUF-16	9.38	3.57	0.89	0.67
MUF-16(Ni)	9.41	3.58	0.89	0.68
MUF-16(Mn)	9.90	3.74	0.93	0.70

*The fraction of the total free volume of MUF-16 that is occupied by adsorbate molecules. This was calculated from the accessible void fraction given by RASPA2 software (Supplementary Table 3), the molecular volume of the CO_2 adsorbates (56.75 Å^3/molecule) and the total number of adsorbate molecules.

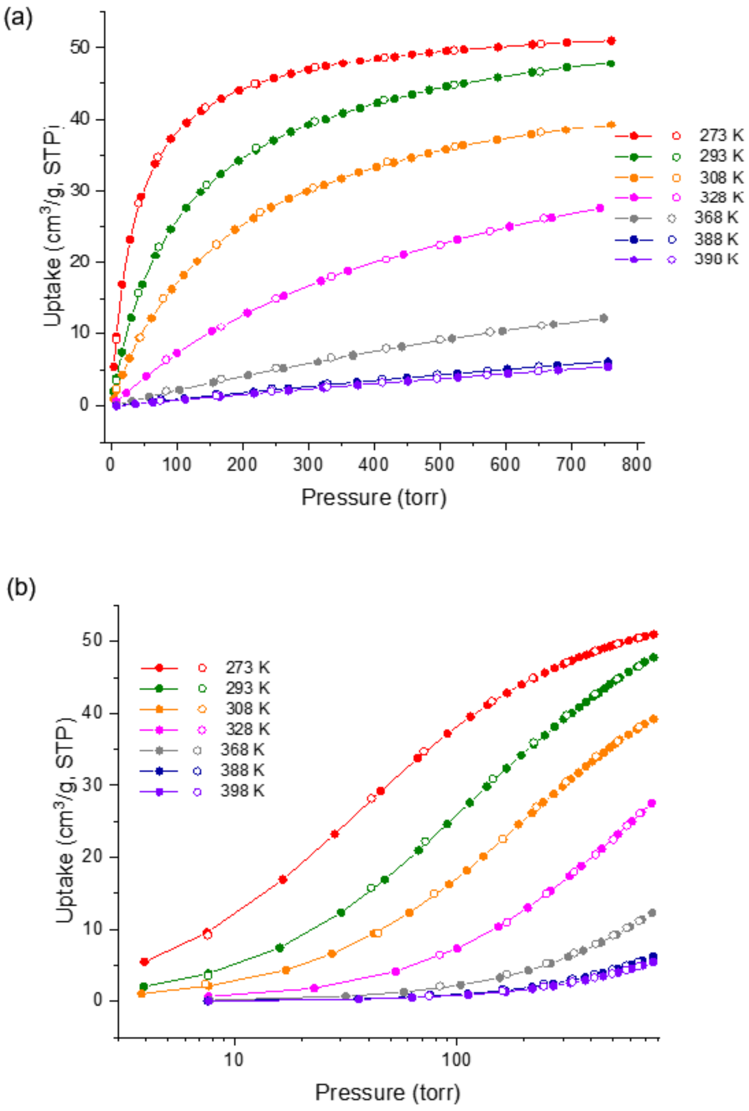

Supplementary Figure 11. Volumetric adsorption (filled circles) and desorption (open circles) isotherms of CO_2 at different temperatures for MUF-16. Isotherms with a linear x axis (a) and on a log scale (b) are shown. Source data are provided as a Source Data file.

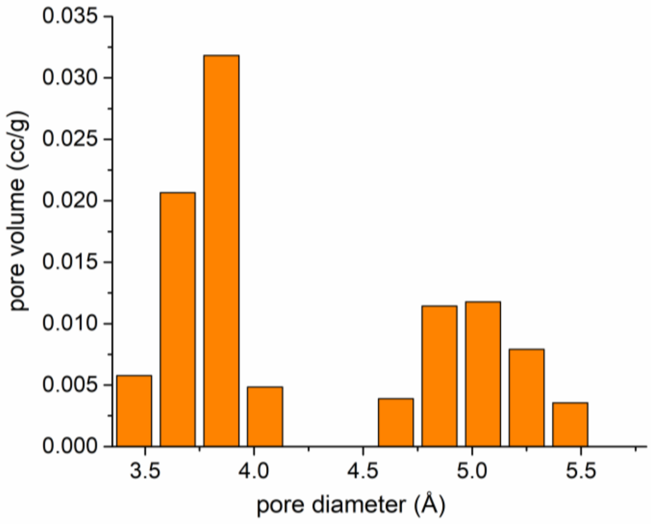

Supplementary Figure 12. Pore size distribution of MUF-16 calculated from its CO_2 adsorption isotherm at 273 K using a NLDFT method. Source data are provided as a Source Data file.

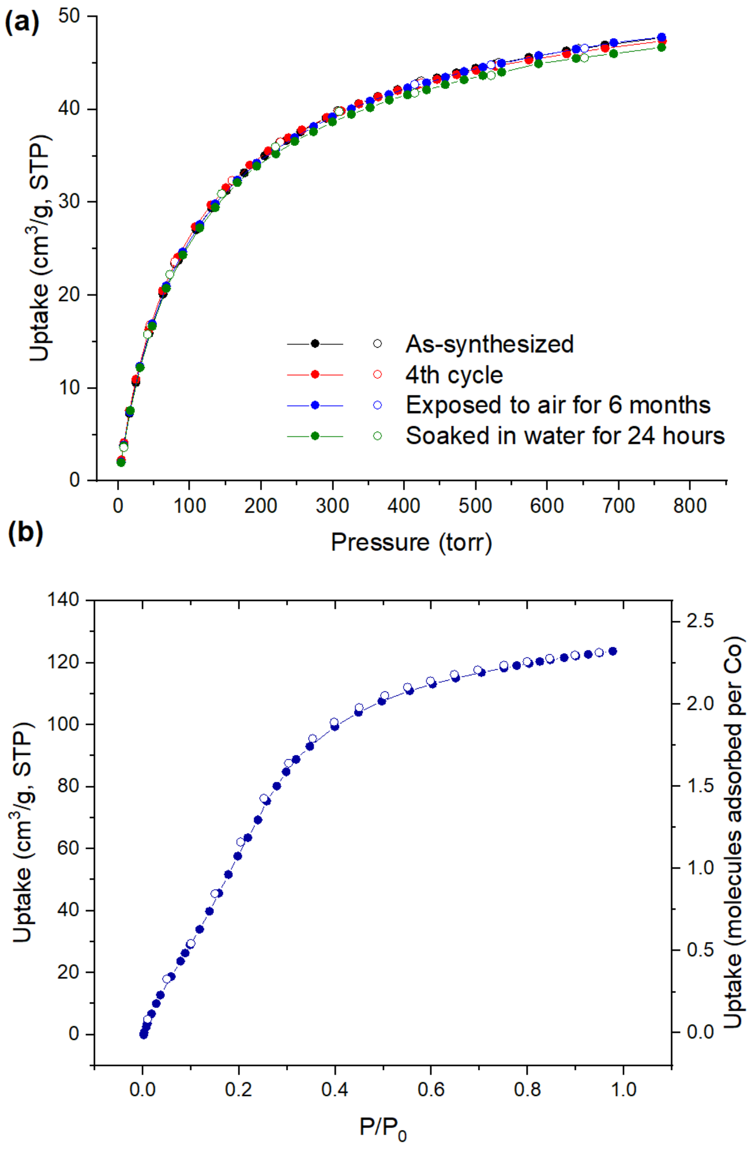

Supplementary Figure 13. (a) CO_2 adsorption isotherms (293 K) of as-synthesized MUF-16 after four consecutive adsorption-desorption cycles, after exposing it to air with ~80% humidity for 6 months, and after immersion in water for 48 hours. (b) Volumetric adsorption (filled circles) and desorption (open circles) isotherms of water vapour measured at 298 K for MUF-16. Source data are provided as a Source Data file.

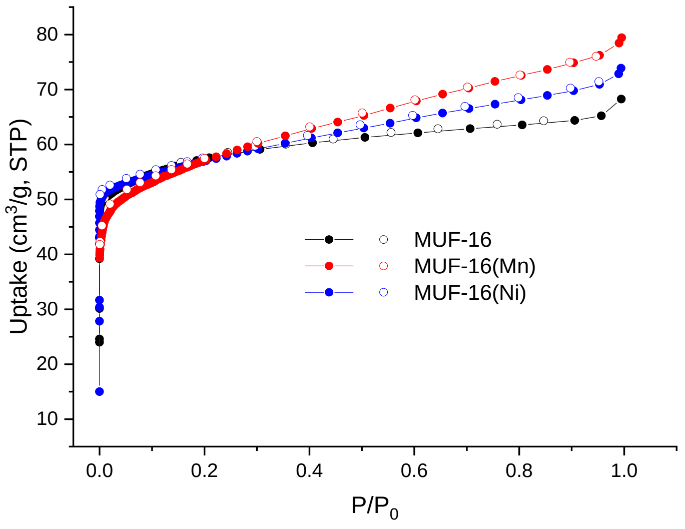

Supplementary Figure 14. Volumetric adsorption (filled circles) and desorption (open circles) isotherms of N_2 for MUF-16 (black), MUF-16(Mn) (red) and MUF-16(Ni) (blue) measured at 77 K. Source data are provided as a Source Data file.

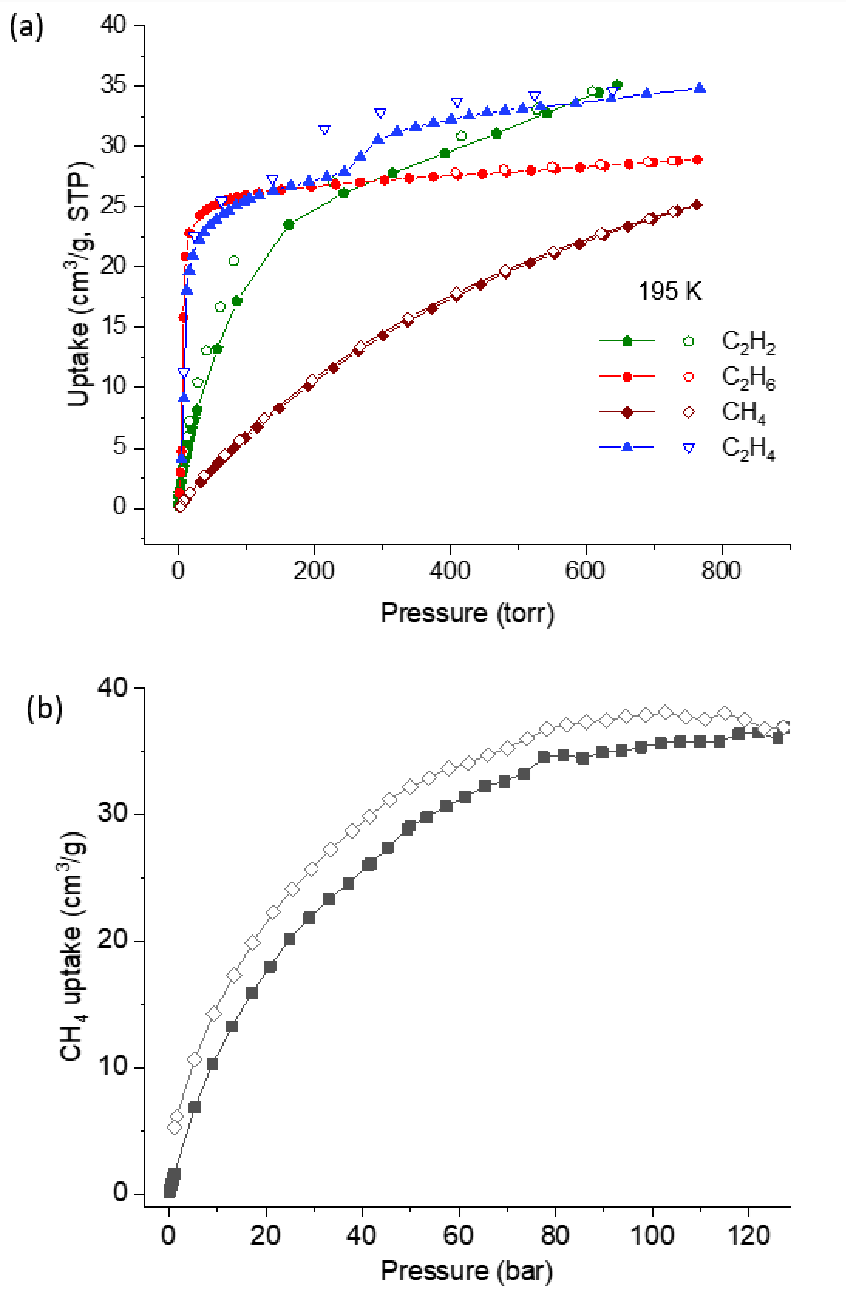

Supplementary Figure 15. (a) Volumetric adsorption (filled shapes) and desorption (open shapes) isotherms of C_2H_2, C_2H_4, C_2H_6 and CH_4 measured at 195 K for MUF-16. (b) High pressure CH_4 uptake by MUF-16 at 293 K showing adsorption (filled shapes) and desorption (open shapes) points. Source data are provided as a Source Data file.

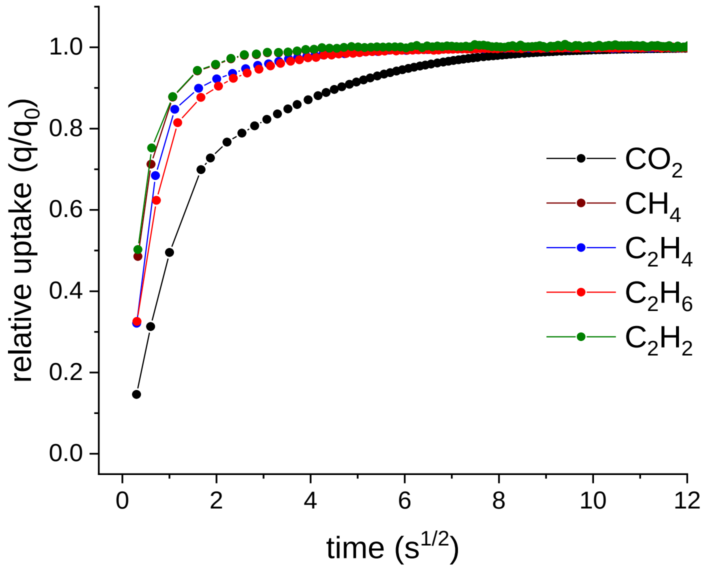

Supplementary Figure 16. Kinetic profiles of different gas uptake by MUF-16 at 293 K upon exposing an evacuated sample to a dose of gas equal to its measured total adsorption of that gas at 1 bar. q is the amount of uptake at time t and q_0 is the final uptake amount. Source data are provided as a Source Data file.

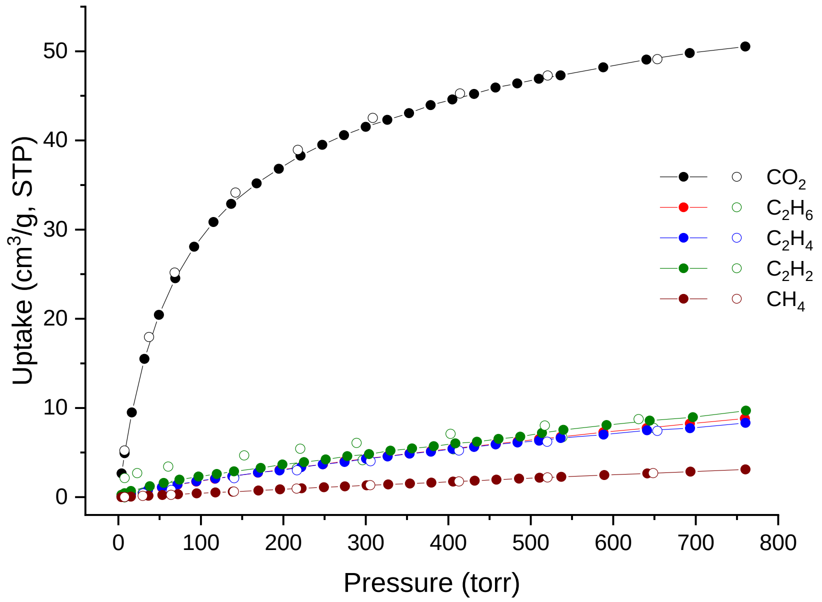

Supplementary Figure 17. Volumetric adsorption (filled circles) and desorption (open circles) isotherms of different gases by MUF-16(Mn) at 293 K.

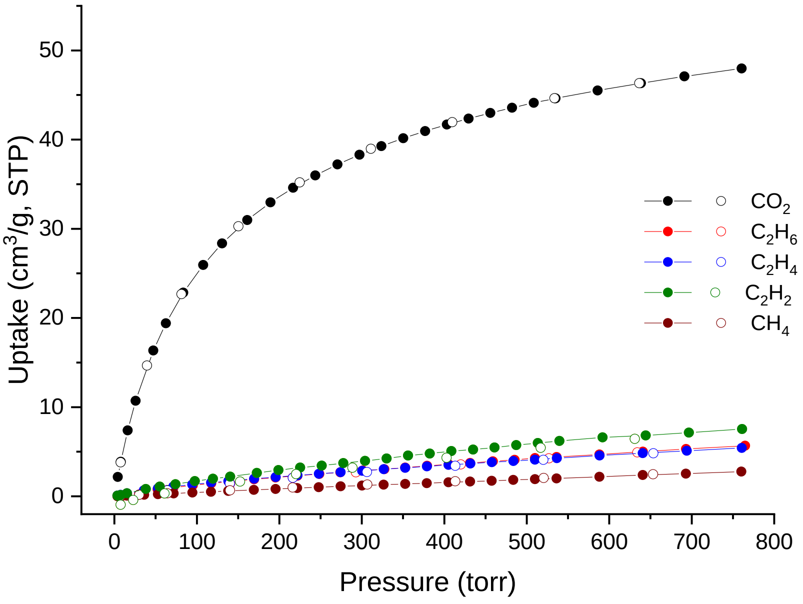

Supplementary Figure 18. Volumetric adsorption (filled circles) and desorption (open circles) isotherms of different gases by MUF-16(Ni) at 293 K.

7. Supplementary Notes: Calculation of BET surface areas

BET surface areas were calculated from N_2 adsorption isotherms at 77 K according to the following procedures[9]:

1) The isotherm region where $v(1 - P/P_0)$ increases versus P/P_0, where v is the amount of N_2 adsorbed, was identified.

2) Within this isotherm region, sequential data points that led to a positive intercept in the plot of $\frac{P/P_0}{v(1-P/P_0)}$ against P/P_0, were found. This plot yields a slope a, and a positive intercept b. The amount of gas molecules adsorbed in the initial monolayer is $v_m = \frac{1}{a+b}$.

3) The BET surface area was calculated according to:

$$A_{BET} = v_m(cm^3 g^{-1}) * \frac{1\ (mol)}{22400\ (cm^3)} * \sigma_0(\text{Å}^2) * N_A(mol^{-1}) * 10^{-20}(\frac{m^2}{\text{Å}^2}) \qquad \text{(Supplementary Equation 1)}$$

Where N_A is Avogadro's constant, and σ_0 is the cross-sectional area of a N_2 molecule, which is 16.2 Å^2.

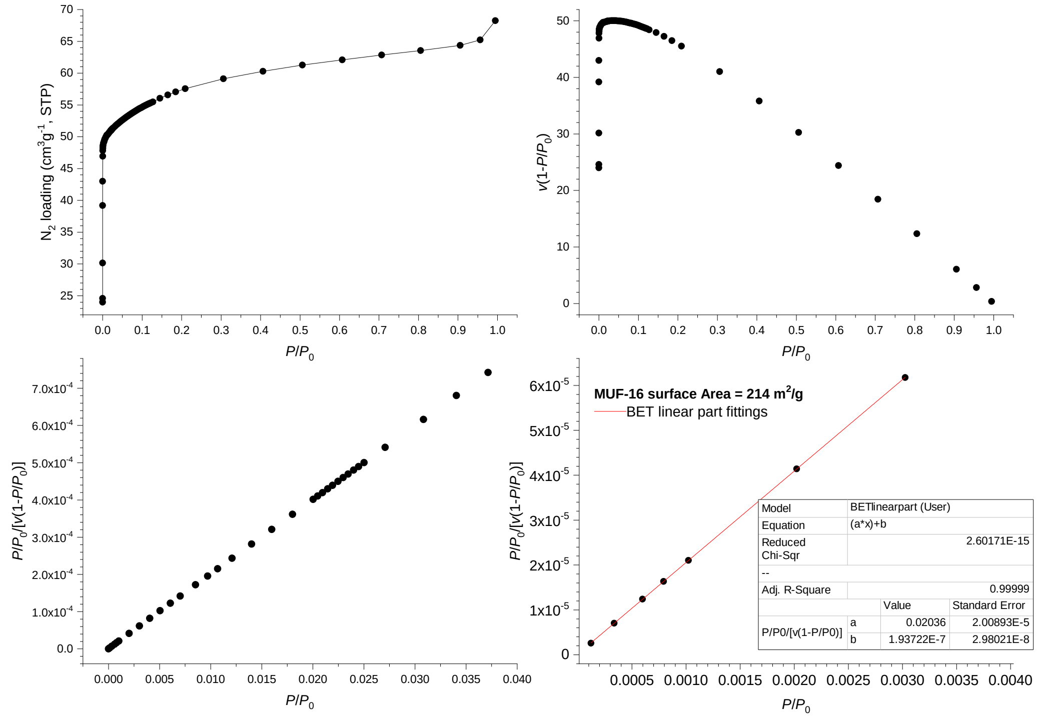

Supplementary Figure 19. N_2 adsorption isotherm at 77 K and BET surface area plots for MUF-16. Source data are provided as a Source Data file.

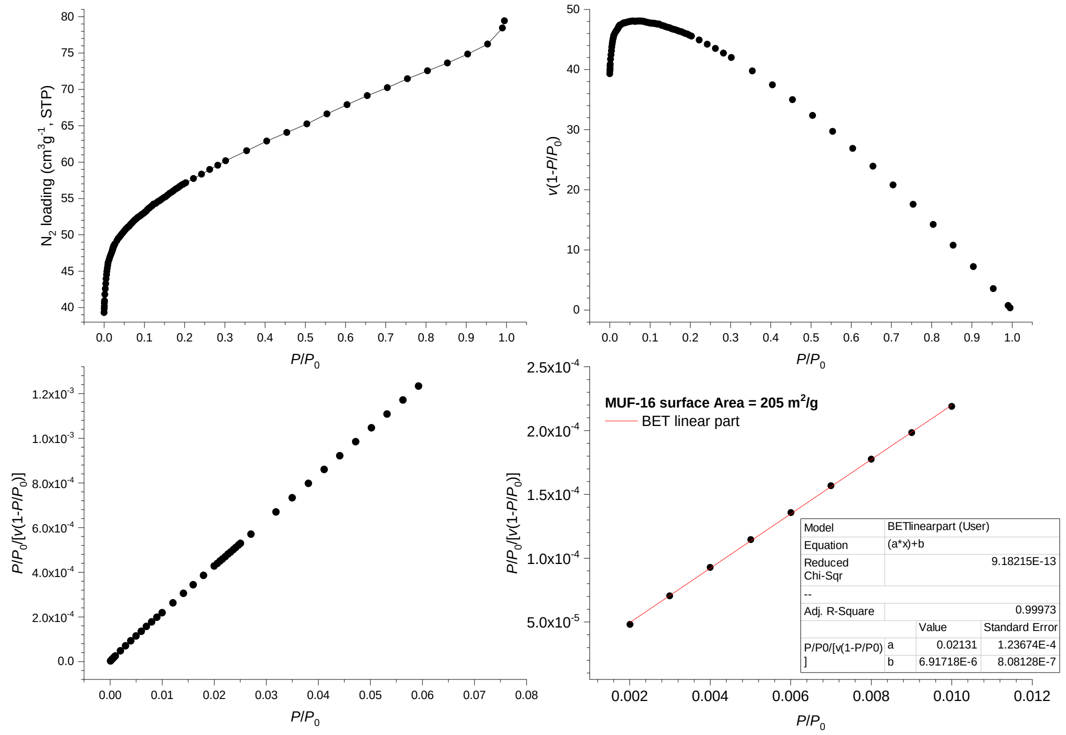

Supplementary Figure 20. N_2 adsorption isotherm at 77 K and BET surface area plots for MUF-16(Mn). Source data are provided as a Source Data file.

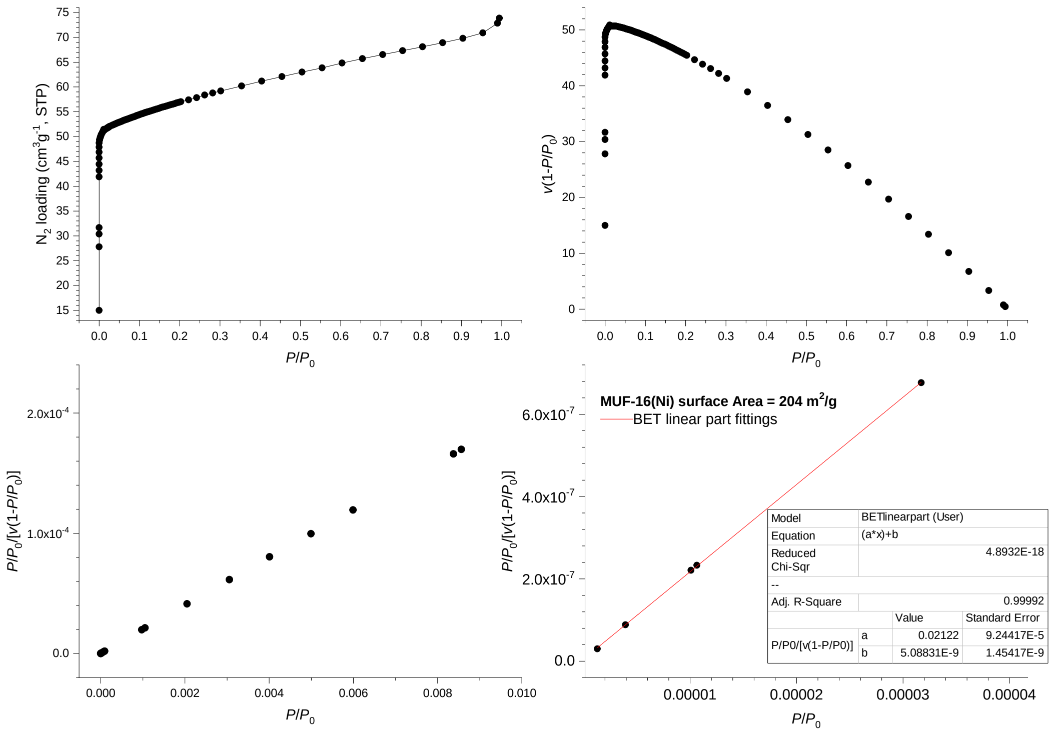

Supplementary Figure 21. N_2 adsorption isotherm at 77 K and BET surface area plots for MUF-16(Ni). Source data are provided as a Source Data file.

8. Supplementary Notes: Heat of adsorption

Isosteric heat of adsorption (Q_{st})[10] values were calculated from isotherms measured at 293K, 298K and 303 K for CO_2. The isotherms were first fit to this viral equation:

$$\ln P = \ln N + \frac{1}{T}\sum_{i=0}^{m} a_i N^i + \sum_{i=0}^{n} b_i N^i \qquad \text{(Supplementary Equation 2)}$$

Where N is the amount of gas adsorbed at the pressure P, a and b are virial coefficients, m and n are the number of coefficients require to adequately describe the isotherm. To calculate Q_{st}, the fitting parameters from the above equation were input in to this equation:

$$Q_{st} = -R\sum_{i=0}^{m} a_i N^i \qquad \text{(Supplementary Equation 3)}$$

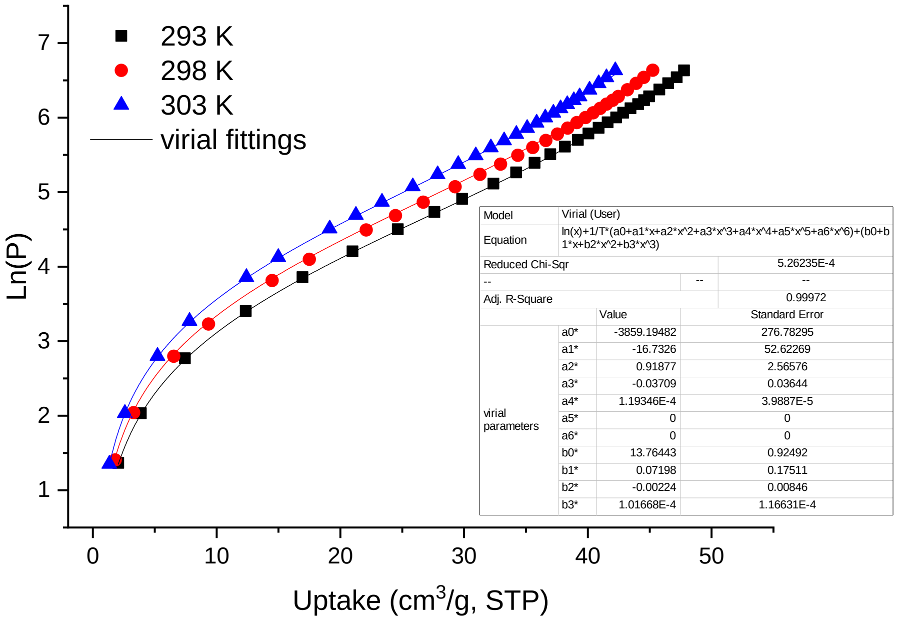

Supplementary Figure 22. Virial equation fits for CO_2 adsorption isotherms of MUF-16.

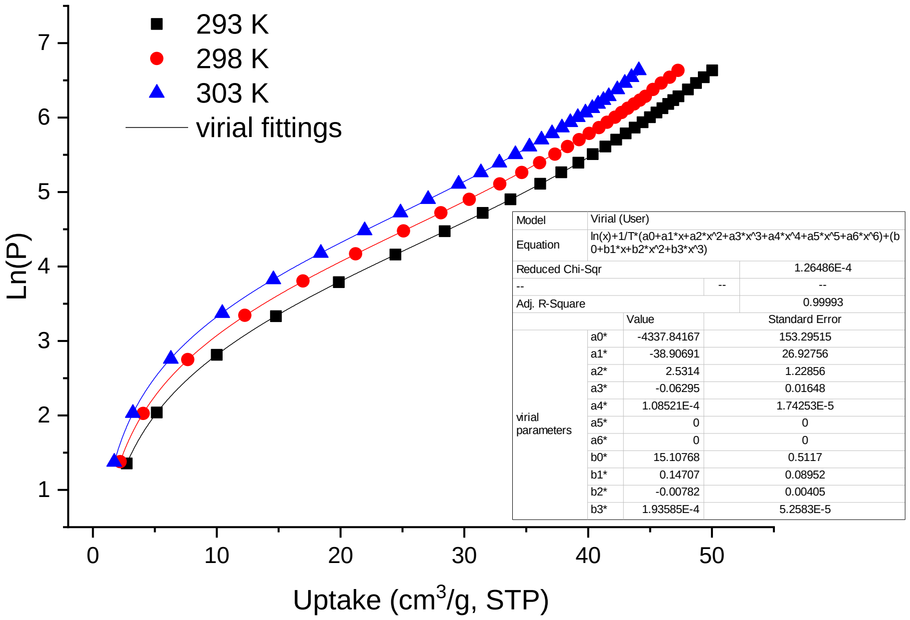

Supplementary Figure 23. Virial equation fits for CO_2 adsorption isotherms of MUF-16(Mn).

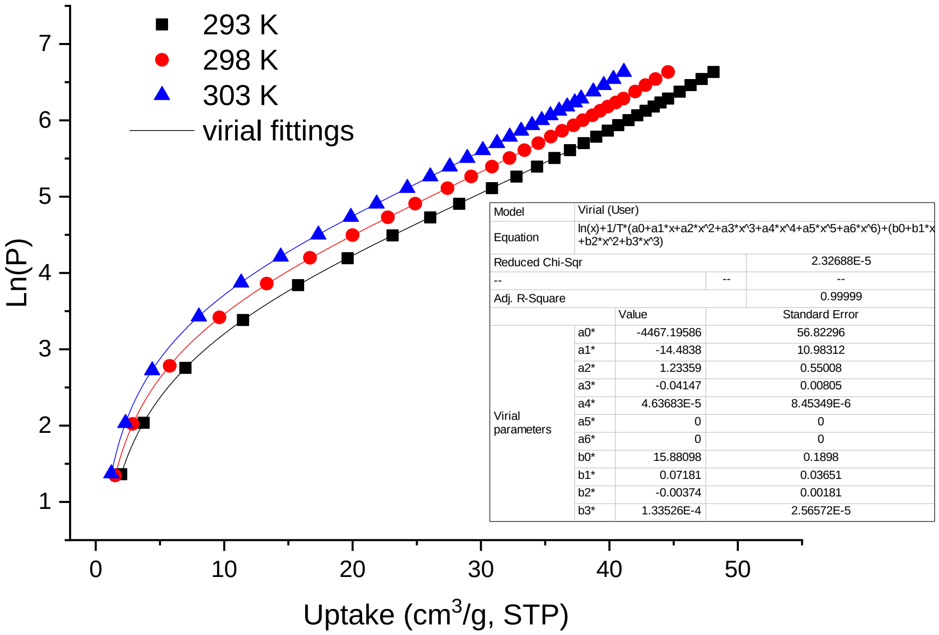

Supplementary Figure 24. Virial equation fits for CO_2 adsorption isotherms of MUF-16(Ni).

Supplementary Table 6. Q_{st} values at low coverage for MUF-16 with various gases.

**gas**	**CO_2**	**C_2H_6**	**C_2H_4**	**C_2H_2**	**CH_4**
**Q_{st} (kJ/mol)**	32.3	24.8	24.9	25.8	18.6

9. Supplementary Notes: IAST calculations

Mixed gas adsorption isotherms and gas selectivities for different mixtures of CO_2/C_2H_2, CO_2/C_2H_4, CO_2/C_2H_6, CO_2/N_2, CO_2/CH_4 and CO_2/H_2 at 293 K were calculated based on the ideal adsorbed solution theory (IAST) proposed by Myers and Prausnitz[11]. In order to predict the sorption performance of MUF-16 toward the separation of binary mixed gases, the single-component adsorption isotherms were first fit to a Dual Site Langmuir or a Dual Site Langmuir Freundlich model, as below:

$$q = \frac{q_1 b_1 P}{1 + b_1 P} + \frac{q_2 b_2 P}{1 + b_2 P} \quad (4)$$

$$q = \frac{q_1 b_1 P^{1/t_1}}{1 + b_1 P^{1/t_1}} + \frac{q_2 b_2 P^{1/t_2}}{1 + b_2 P^{1/t_2}} \quad (5)$$

Where q is the uptake of a gas; P is the equilibrium pressure and q_1, b_1, t_1, q_2, b_2 and t_2 are constants. These parameters were subsequently used for the IAST calculations.

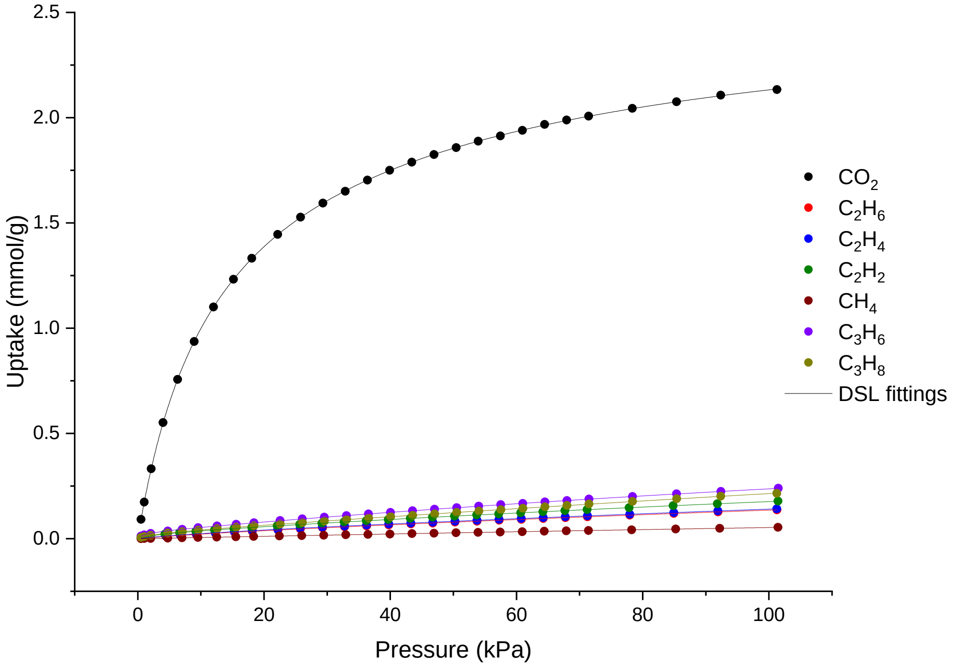

Model	DSL (User)						
Equation	q1*b1*x/(1+b1*x)+q2*b2*x/(1+b2*x)						
Plot	CO2	C2H6	C2H4	C2H2	CH4	C3H6	C3H8
q1	1.92197 ± 0.03622	0.01903 ± 7.58427E-4	0.90455 ± 0.02194	0.84858 ± 0.02172	-1.37048E-4 ± 2.62012E-5	0.92327 ± 0.02031	0.02582 ± 8.46918E-4
b1	0.09147 ± 0.00142	0.06919 ± 0.00295	0.00155 ± 4.83418E-5	0.00219 ± 7.32671E-5	2.40335 ± 2.41269	0.00289 ± 8.52827E-5	0.38985 ± 0.03262
q2	0.71591 ± 0.02029	0.91643 ± 0.02976	0.02077 ± 4.66874E-4	0.02486 ± 4.712E-4	0.63958 ± 0.01292	0.03023 ± 4.69954E-4	2.11273 ± 0.21651
b2	0.01264 ± 0.0014	0.00149 ± 6.32966E-5	0.09736 ± 0.00278	0.29065 ± 0.01171	9.04454E-4 ± 2.00076E-5	0.92871 ± 0.04824	9.83565E-4 ± 1.13365E-4
Reduced Chi-Sqr	2.547E-6	4.27057E-9	4.09689E-9	3.87189E-8	1.11837E-9	1.63183E-7	2.34956E-7
R-Square (COD)	0.99999	1	1	0.99999	1	0.99997	0.99994
Adj. R-Square	0.99999	1	1	0.99998	1	0.99996	0.99994

Supplementary Figure 25. Dual-site Langmuir fits of various adsorption isotherms for MUF-16 at 293 K.

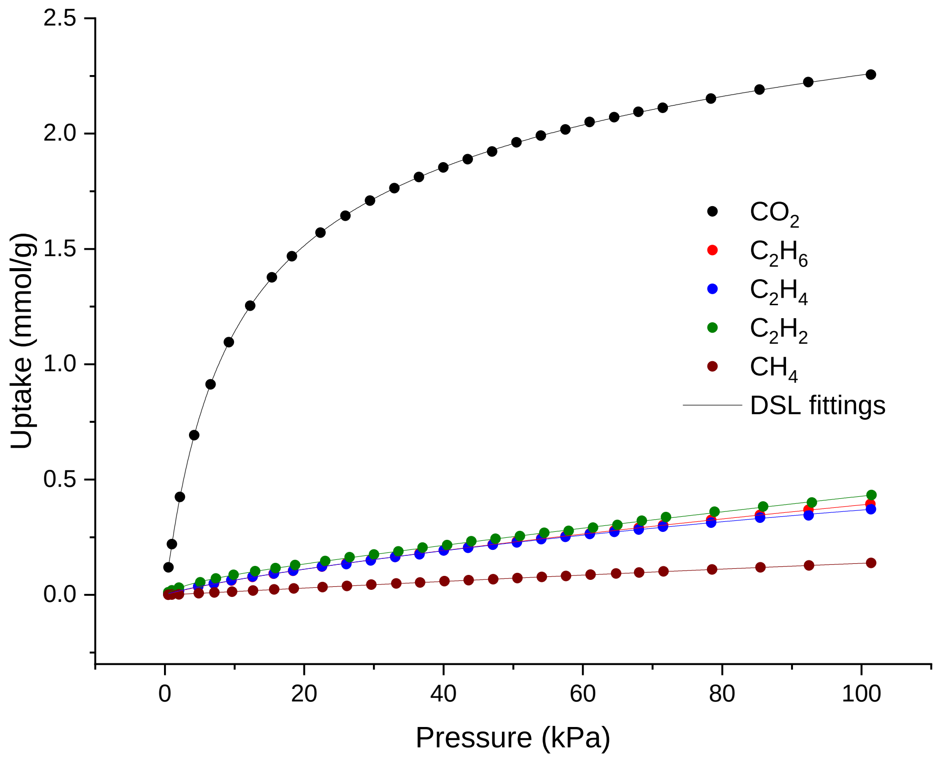

Model	DSL (User)				
Equation	q1*b1*x/(1+b1*x)+q2*b2*x/(1+b2*x)				
Plot	CO2	C2H6	C2H4	C2H2	CH4
q1	1.94289 ± 0.0227	0.04806 ± 0.00391	1.11314 ± 0.0652	3.94913 ± 1.20449	1.20612 ± 0.06748
b1	0.12229 ± 0.00169	0.09141 ± 0.009	0.00448 ± 4.81593E-	0.00102 ± 3.53725E-	0.00129 ± 8.15601E-5
q2	1.14216 ± 0.04571	2.04938 ± 0.09902	0.02464 ± 0.00777	0.06637 ± 0.00631	-8.32165E-4 ± 2.43966E
b2	0.0067 ± 8.46612E-	0.00204 ± 1.38007E-	0.20141 ± 0.10616	0.24669 ± 0.04921	10.03567 ± 38.12063
Reduced Chi-Sq	6.81414E-6	2.15657E-7	3.7049E-6	6.09794E-6	1.33437E-7
R-Square (COD)	0.99998	0.99999	0.99973	0.99963	0.99993
Adj. R-Square	0.99998	0.99998	0.99969	0.99958	0.99992

Supplementary Figure 26. Dual-site Langmuir fits of various adsorption isotherms for MUF-16(Mn) at 293 K.

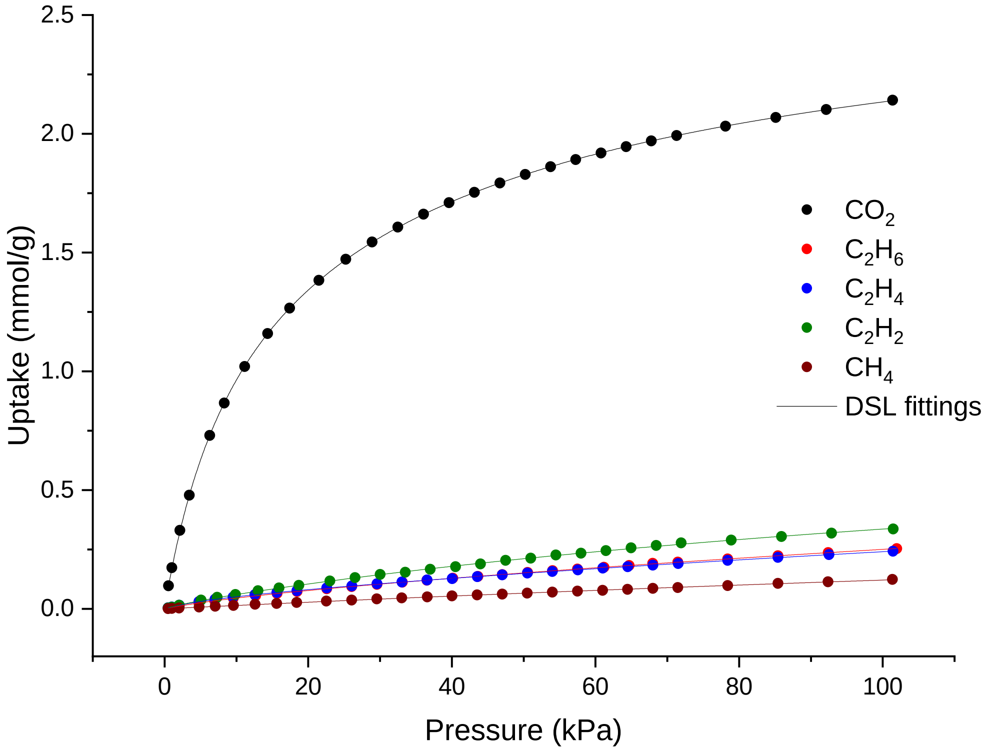

Model	DSL (User)				
Equation	q1*b1*x/(1+b1*x)+q2*b2*x/(1+b2*x)				
Plot	CO2	C2H6	C2H4	C2H2	CH4
q1	1.57274 ± 0.02824	0.05244 ± 0.00708	1.27493 ± 0.09994	0.88496 ± 0.04798	0.74301 ± 0.05154
b1	0.10503 ± 0.00153	0.07063 ± 0.01023	0.00176 ± 1.81686E-4	0.0056 ± 6.32939E-4	0.00195 ± 1.53031E-4
q2	1.12788 ± 0.01983	1.38112 ± 0.20289	0.05362 ± 0.00249	0.01883 ± 0.00847	0 ± 0
b2	0.01624 ± 7.28604E-4	0.00174 ± 3.48805E-4	0.1148 ± 0.00734	0.23099 ± 0.18081	0 ± 0
Reduced Chi-Sqr	1.49036E-6	3.73129E-7	1.65794E-7	5.0543E-6	7.98591E-7
R-Square (COD)	1	0.99994	0.99997	0.99956	0.99946
Adj. R-Square	1	0.99993	0.99997	0.99951	0.99939

Supplementary Figure 27. Dual-site Langmuir fits of various adsorption isotherms for MUF-16(Ni) at 293 K.

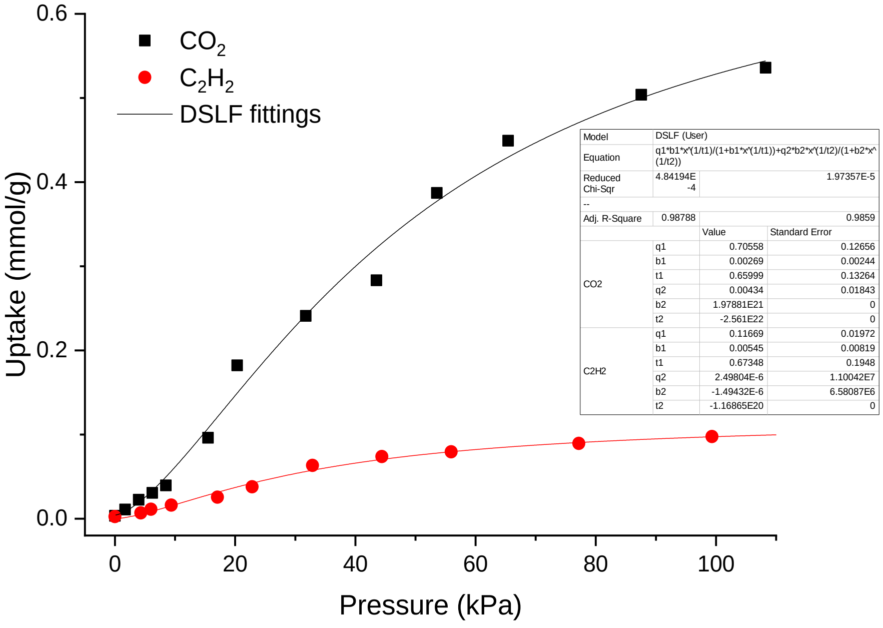

Supplementary Figure 28. Dual-site Langmuir Freundlich fits for $K_2[Cr_3O(OOCH)_6(4\text{-}ethylpyridine)_3]_2[\alpha\text{-}SiW_{12}O_{40}]$ at 278 K. Isotherm data were extracted from [6] using a digitizer software.

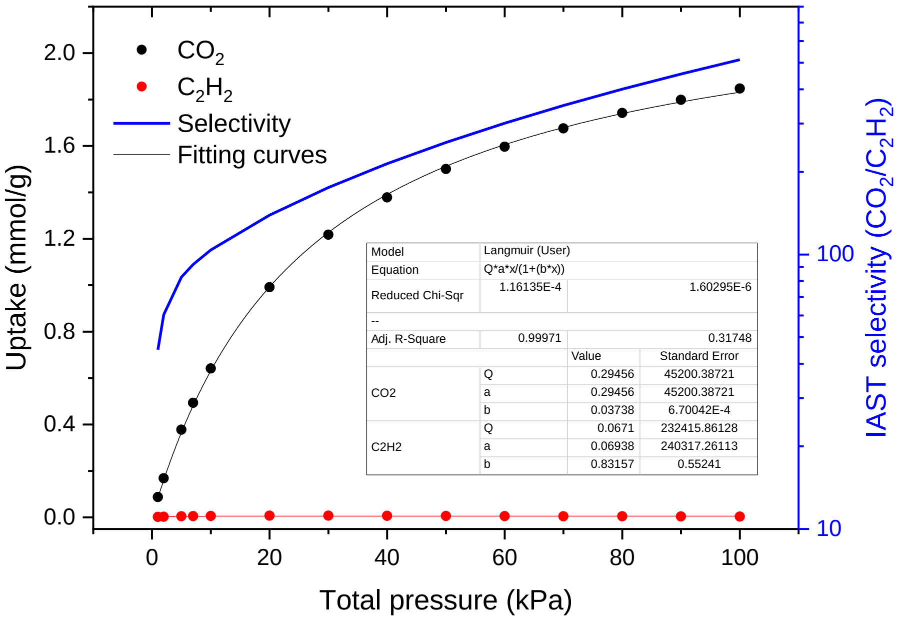

Supplementary Figure 29. Mixed-gas isotherms and selectivity of MUF-16 predicted by IAST for a mixture of 50/50 CO_2/C_2H_2 at 293 K.

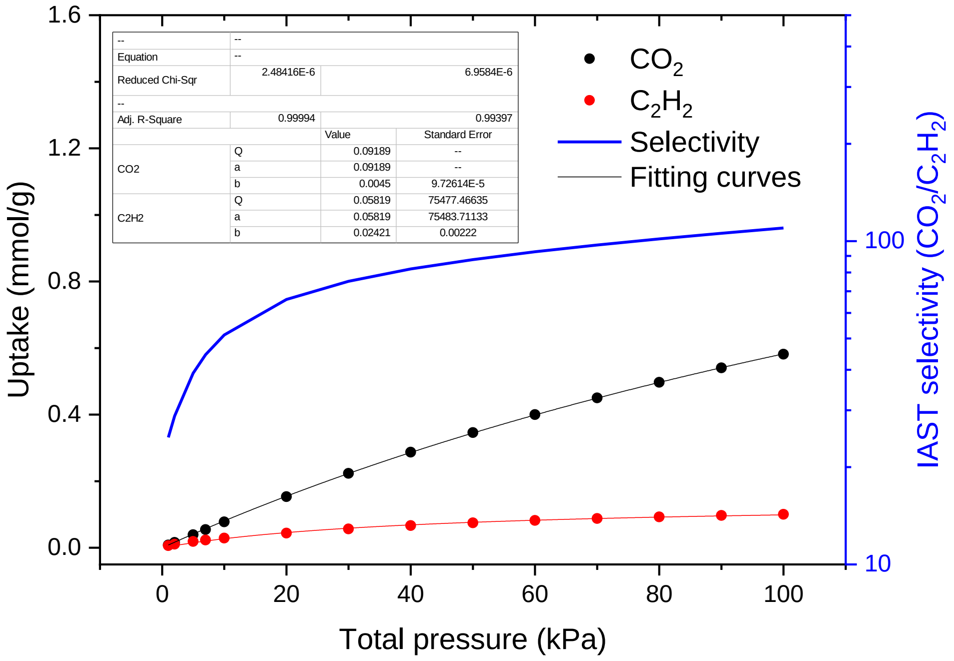

Supplementary Figure 30. Mixed-gas isotherms and selectivity of MUF-16 predicted by IAST for a mixture of 5/95 CO_2/C_2H_2 at 293 K.

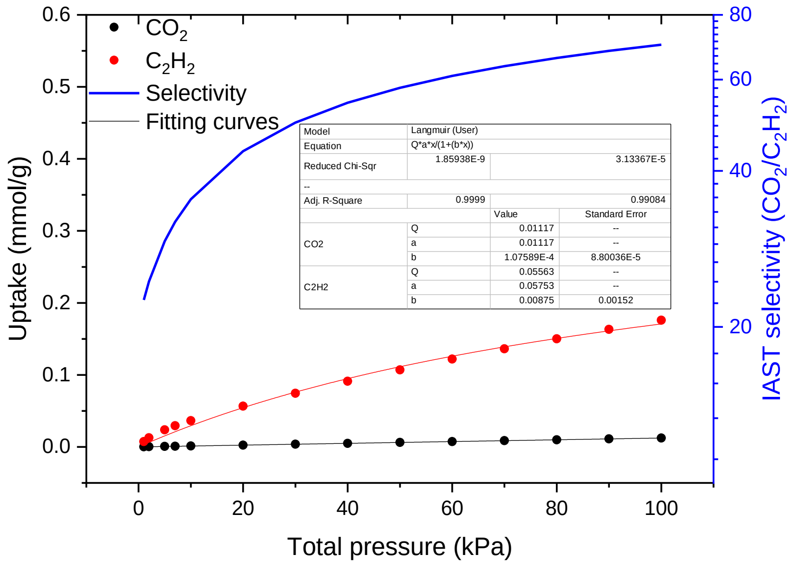

Supplementary Figure 31. Mixed-gas isotherms and selectivity of MUF-16 predicted by IAST for a mixture of 0.1/99.9 CO_2/C_2H_2 at 293 K.

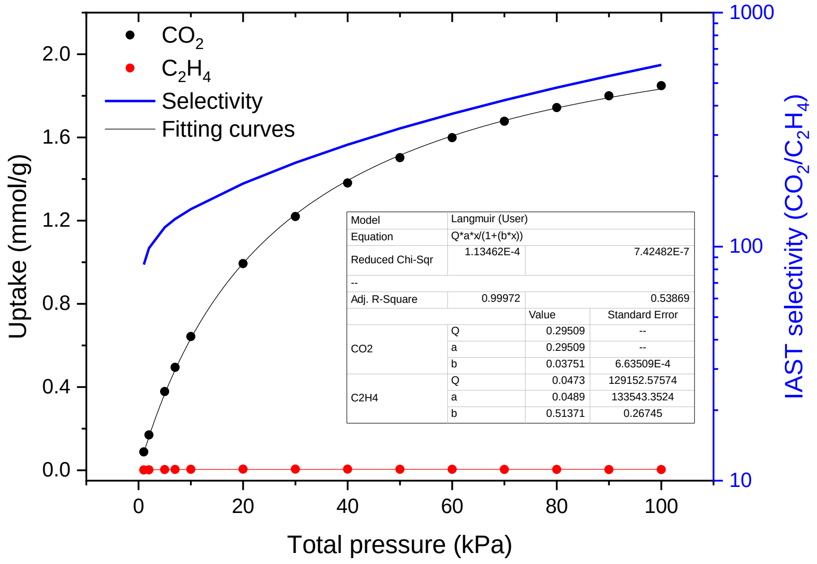

Supplementary Figure 32. Mixed-gas isotherms and selectivity of MUF-16 predicted by IAST for a mixture of 50/50 CO_2/C_2H_4 at 293 K.

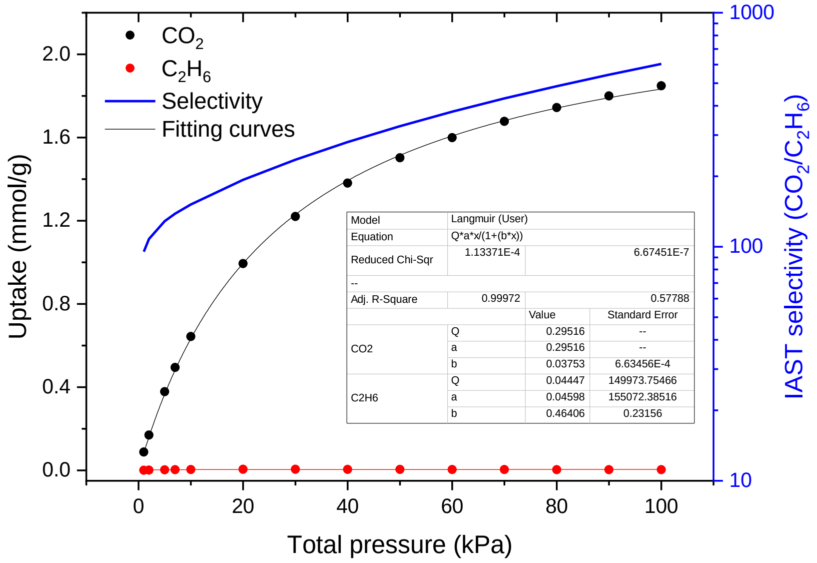

Supplementary Figure 33. Mixed-gas isotherms and selectivity of MUF-16 predicted by IAST for a mixture of 50/50 CO_2/C_2H_6 at 293 K.

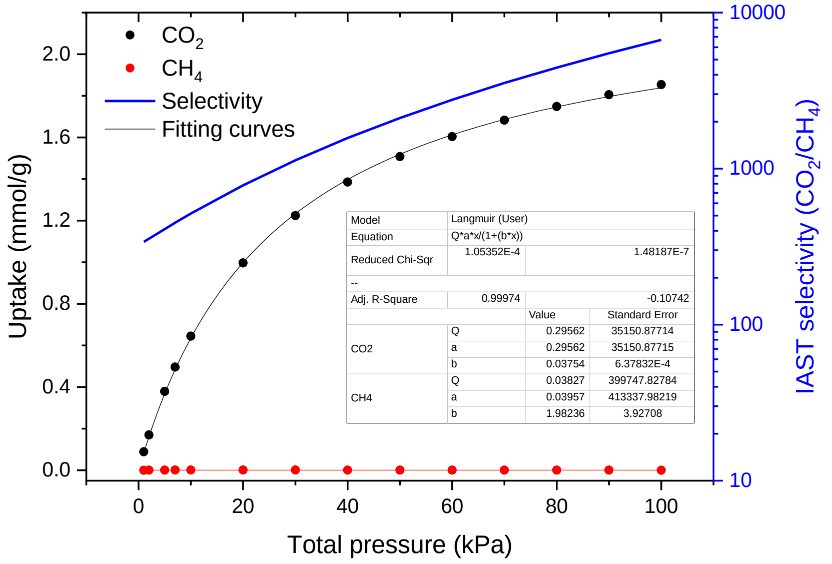

Supplementary Figure 34. Mixed-gas isotherms and selectivity of MUF-16 predicted by IAST for a mixture of 50/50 CO_2/CH_4 at 293 K.

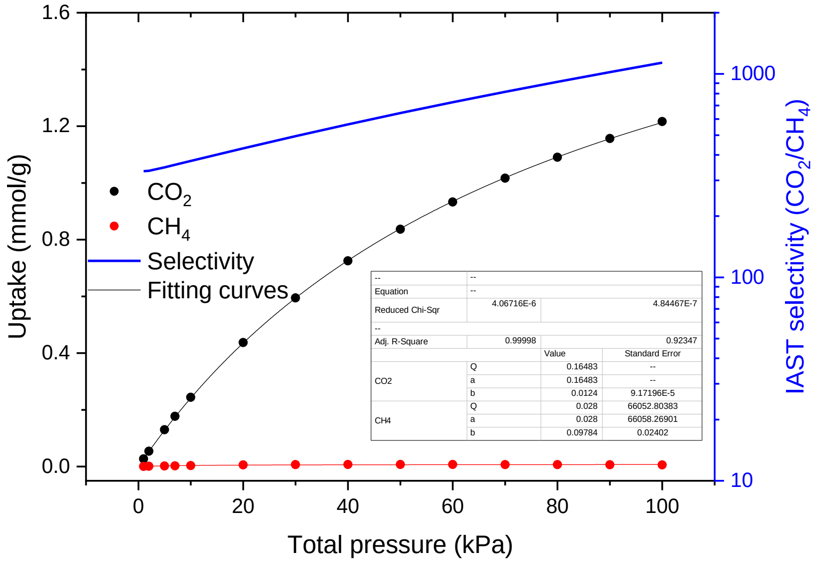

Supplementary Figure 35. Mixed-gas isotherms and selectivity of MUF-16 predicted by IAST for a mixture of 15/85 CO_2/CH_4 at 293 K.

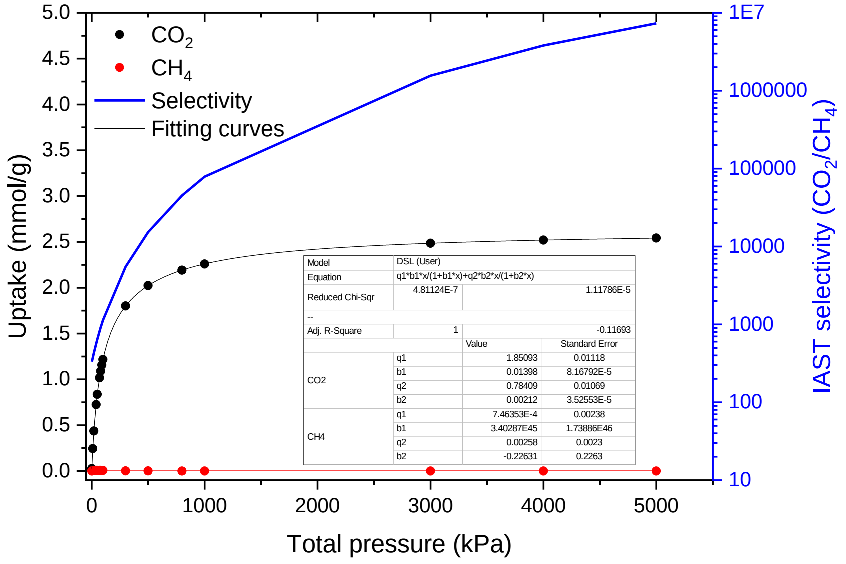

Supplementary Figure 36. Mixed-gas isotherms and selectivity of MUF-16 predicted by IAST for a mixture of 15/85 CO_2/CH_4 at 293 K up to 50 bar.

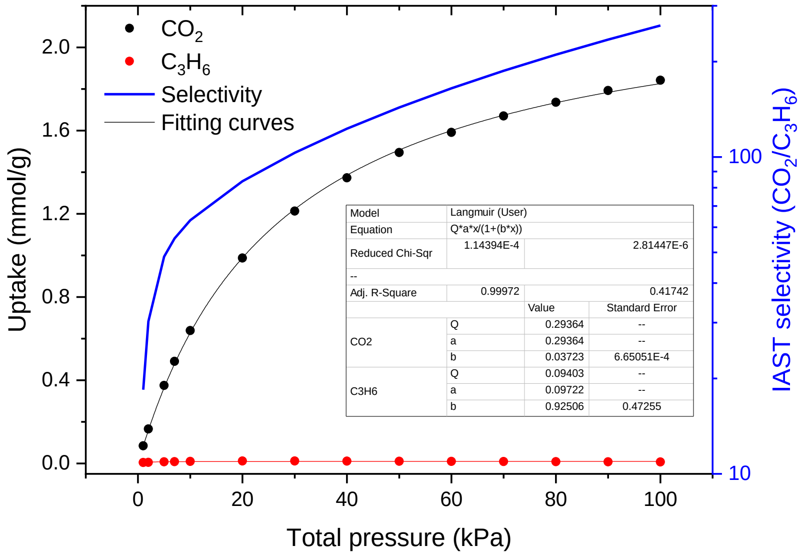

Supplementary Figure 37. Mixed-gas isotherms and selectivity of MUF-16 predicted by IAST for a mixture of 50/50 CO_2/C_3H_6 at 293 K.

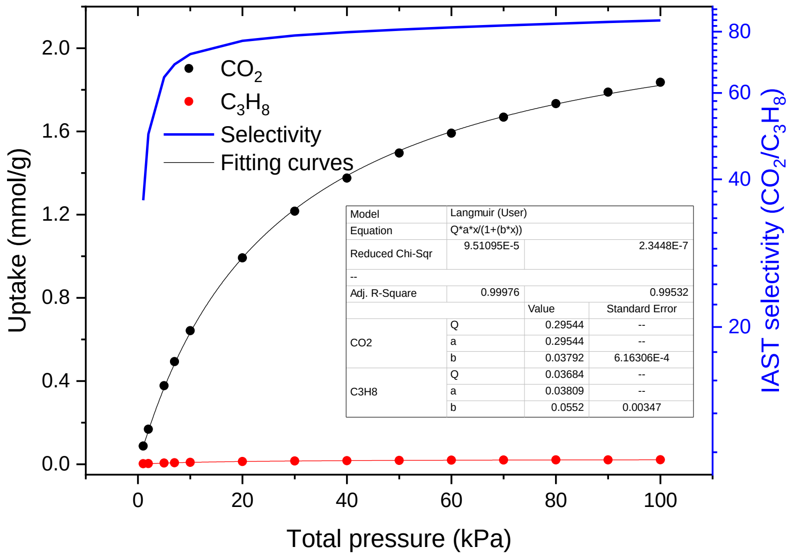

Supplementary Figure 38. Mixed-gas isotherms and selectivity of MUF-16 predicted by IAST for a mixture of 50/50 CO_2/C_3H_8 at 293 K.

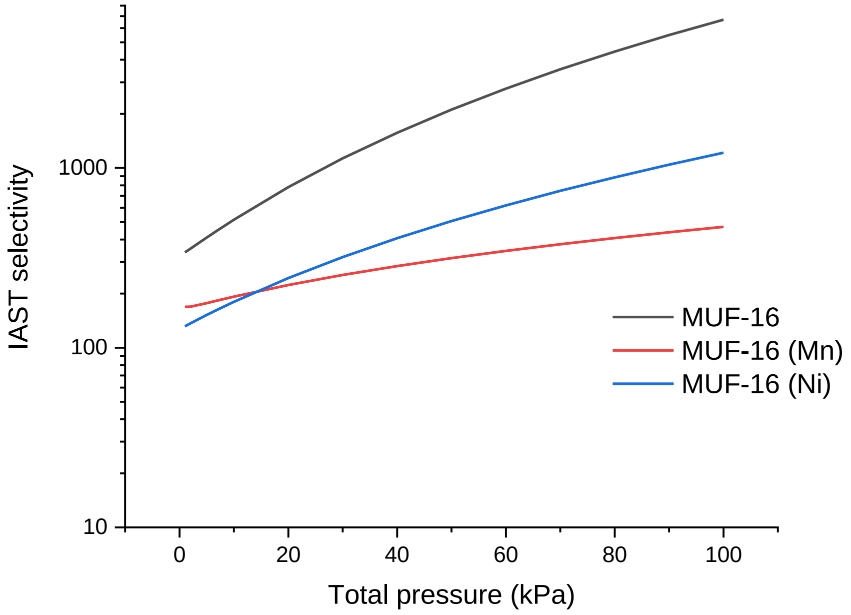

Supplementary Figure 39. IAST selectivity for a 50/50 mixture of CO_2/CH_4 at 293 K for the MUF-16 family.

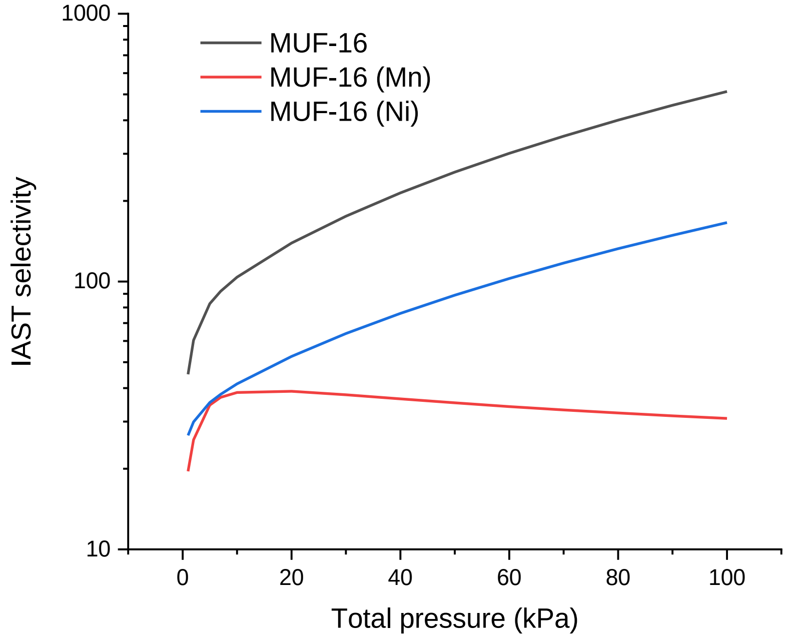

Supplementary Figure 40. IAST selectivity for a 50/50 mixture of CO_2/C_2H_2 at 293 K for the MUF-16 family.

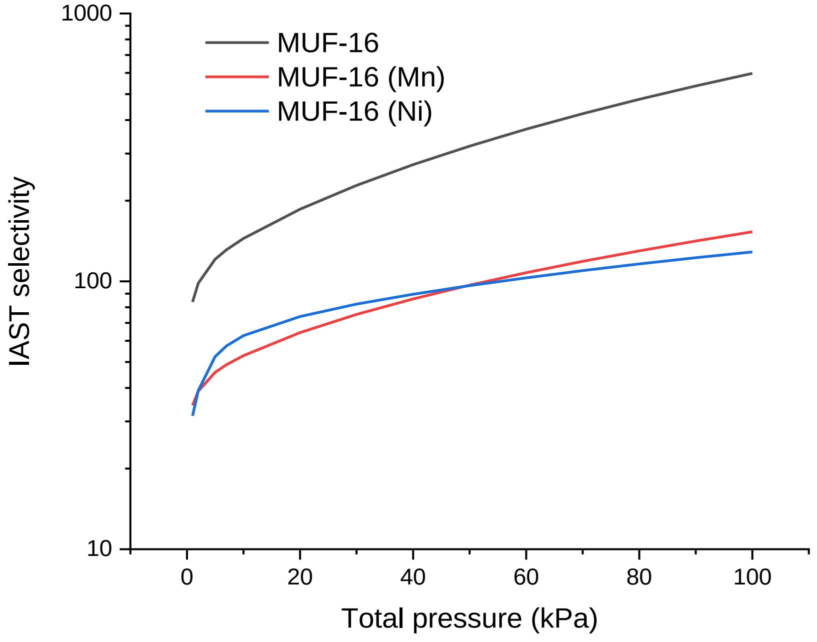

Supplementary Figure 41. IAST selectivity for a 50/50 mixture of CO_2/C_2H_4 at 293 K for the MUF-16 family.

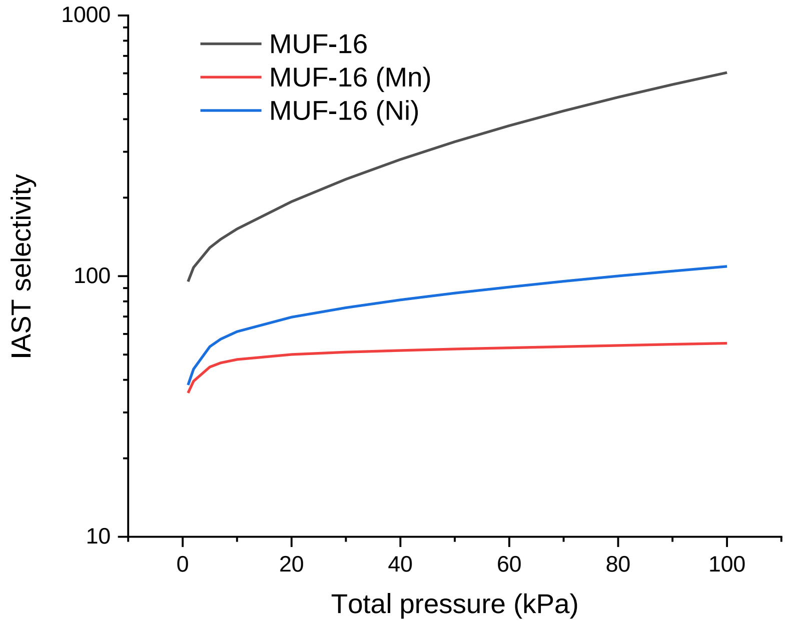

Supplementary Figure 42. IAST selectivity for a 50/50 mixture of CO_2/C_2H_6 at 293 K for the MUF-16 family.

10. Supplementary Methods: Breakthrough separation experiments and simulations

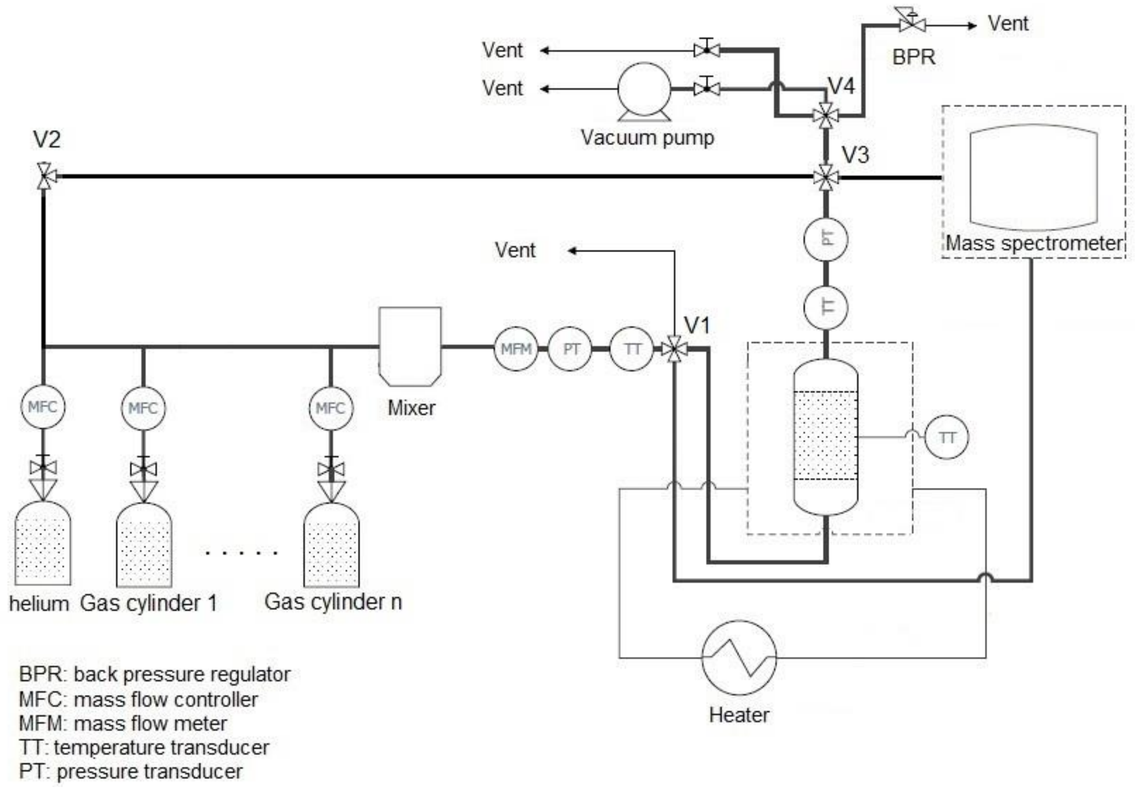

Supplementary Figure 43. A schematic of the experimental column breakthrough setup.

Supplementary Table 7. Summary of inlet gas feed streams, outlet compositions and associated data for experimental breakthrough tests using a MUF-16 adsorbent bed.

Gas mixture	Total pressure (bar)	Inlet CO_2 partial pressure (bar)	Flowrate (mL_N/min)	Upper limit for CO_2 concentration in effluent (ppmv)	Breakthrough point of CO_2 (min)	CO_2 concentration in effluent at breakthrough point (ppmv)	Dynamic adsorption capacity (mmol/g)	Equilibrium adsorption capacity (mmol/g)
CO_2/CH_4 (50/50)	1	0.5	6	500	10.6	600	1.53	1.85
CO_2/CH_4 (15/85)	1	0.15	6	520	25.6	600	1.13	1.23
CO_2/CH_4 (15/85)	9	0.15	6	360	44.8	600	2.01	-
$CO_2/CH_4+C_2H_6+C_3H_8$ (15/80/4/1)	1	0.15	6	520	24.6	600	1.09	1.23
$CO_2/CH_4+C_2H_6+C_3H_8$ (15/80/4/1)	9	0.15	6	390	42.5	600	1.93	-
CO_2/C_2H_2 (50/50)*	1	0.33	6	500	12.3	600	1.23	1.64
CO_2/C_2H_2 (5/95)	1	0.035	6.85	540	15.1	600	0.18	0.46
CO_2/C_2H_4 (50/50)*	1	0.33	6	500	11.9	600	1.19	1.64
CO_2/C_2H_6 (50/50)*	1	0.33	6	500	12.2	600	1.22	1.64

* 2 mL_N/min of helium was used as carrier gas in this experiment.

10.1. CO_2/CH_4 and $CO_2/CH_4+C_2H_6+C_3H_8$ breakthrough separations

Activated MUF-16 (0.9 g) was placed in an adsorption column (6.4 mm in diameter × 11 cm in length) to form a fixed bed. The adsorbent was activated at 130 °C under high vacuum for 7 hours and then the column was left under vacuum for another 3 hours while being cooled to 20 °C. The column was then purged under a 20 mL_N/min flow of He gas for 1 hr at 1.1 bar prior to the breakthrough experiment. A gas mixture containing CO_2/CH_4 or CO_2/ CH_4+ $C_2H_6+C_3H_8$ was introduced to the column at 1.1 bar and 9 bar for CO_2/CH_4 and $CO_2/CH_4+C_2H_6+C_3H_8$) and 20 °C.

A feed flowrate of 6 mL_N/min was set. The operating pressure was controlled at 1.1 or 9 bar with a back-pressure regulator. The outlet composition was continuously monitored by a SRS UGA200 mass spectrometer. The CO_2 was deemed to have broken through from the column when its concentration reached 600 ppmv.

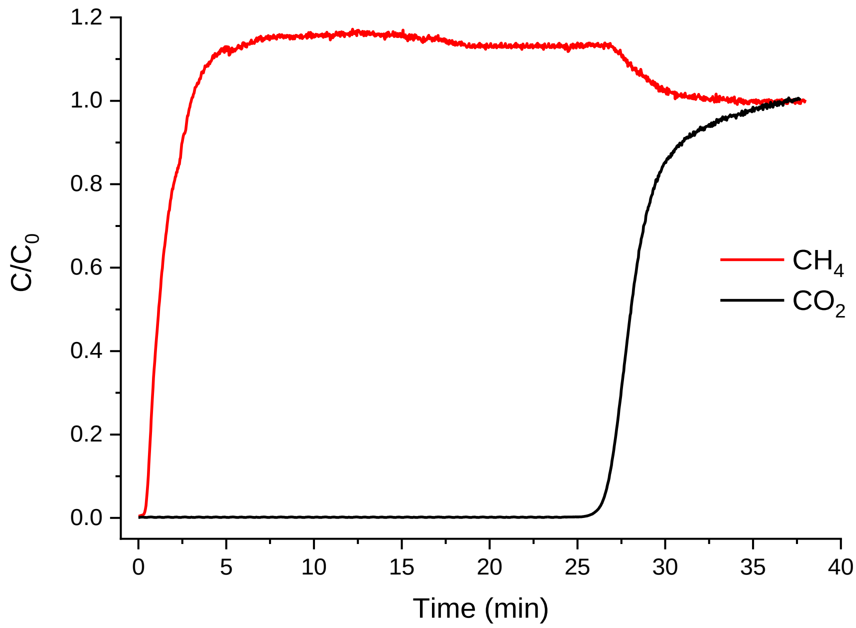

Supplementary Figure 44. Experimental breakthrough curves for a mixture of 15/85 CO_2/CH_4 at 1.1 bar and 293 K in an adsorption column packed with MUF-16.

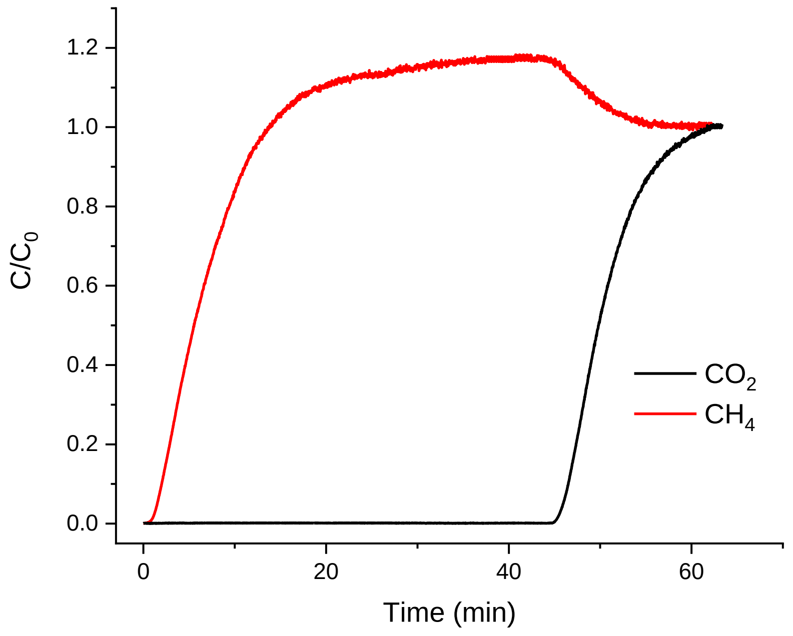

Supplementary Figure 45. Experimental breakthrough curves for a mixture of 15/85 CO_2/CH_4 at 9 bar and 293 K in an adsorption column packed with MUF-16.

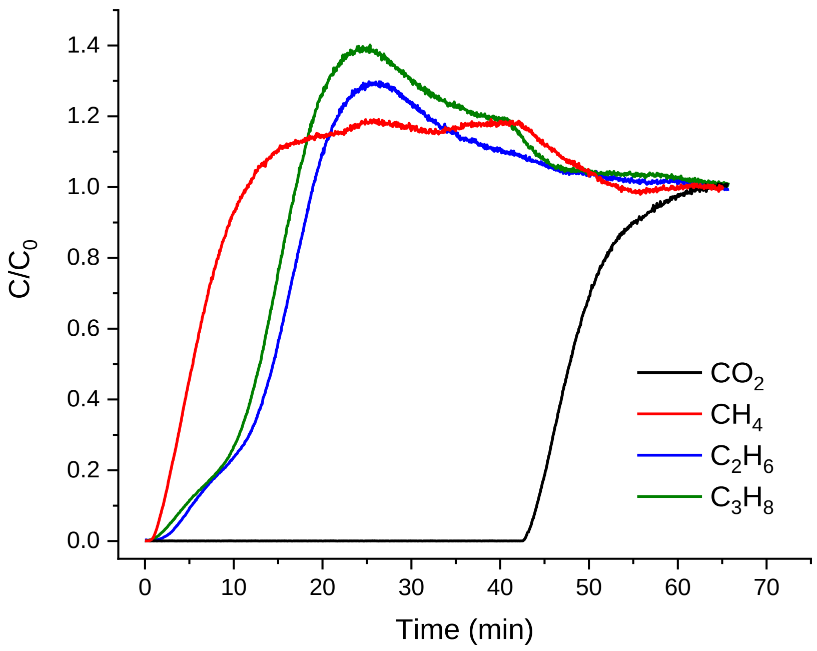

Supplementary Figure 46. Experimental breakthrough curves for a mixture of 15/80/4/1 CO_2/CH_4/C_2H_6/C_3H_8 at 9 bar and 293 K in an adsorption column packed with MUF-16.

10.1.1. Simulations of CO_2/CH_4 breakthrough curves

The simulation of breakthrough curves was carried out using a previously reported method.[12, 13] A value for the mass transfer coefficient (k) was obtained by empirical tuning the steepness of the predicted breakthrough curves to match the experimental curve. The mass transfer coefficient tuned in this way was later used to predict breakthrough curves for other feed mixtures and operating pressures. A summary of adsorption column parameters and feed characterizations are presented in Supplementary Table 8.

Supplementary Table 8. Adsorption column parameters and feed characterizations used for the simulations for MUF-16.

**Adsorption bed**	**Feed**
Length: 110 mm<br>Diameter: 6.4 mm<br>Amount of adsorbent in the bed: 0.9 g<br>Adsorbent density: 1.674 g/cm^3<br>Adsorbent average radius: 0.2 mm<br>k_{CO2}: 0.029 s^{-1}<br>k_{CH4}: 0.00021 s^{-1}	Flow rate: 6 mL$_N$/min<br>Temperature: 293 K<br>Pressure: 1.1 bar<br>Carrier gas flow rate: No carrier gas was used

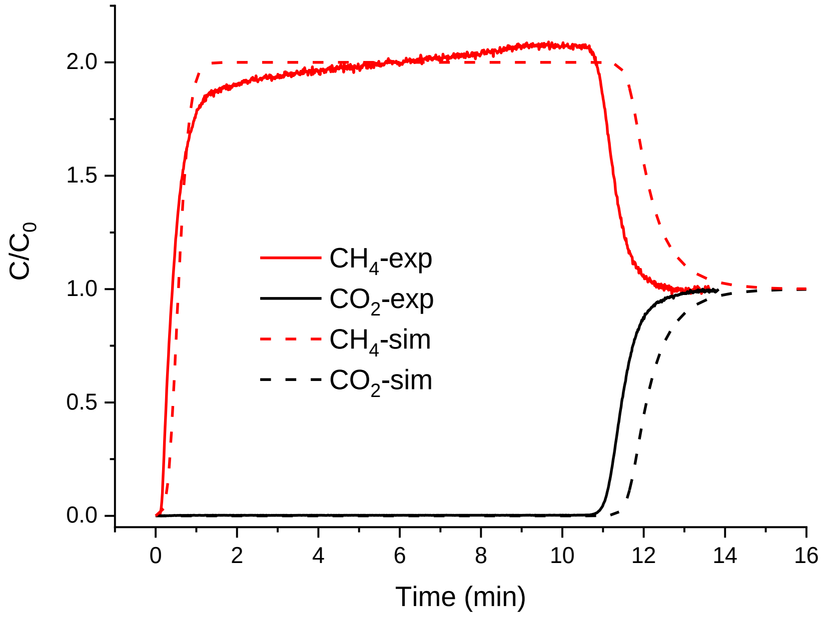

Supplementary Figure 47. Experimental breakthrough curves in comparison to simulated one for a mixture of 50/50 CO_2/CH_4 at 1.1 bar and 293 K in an adsorption column packed with MUF-16.

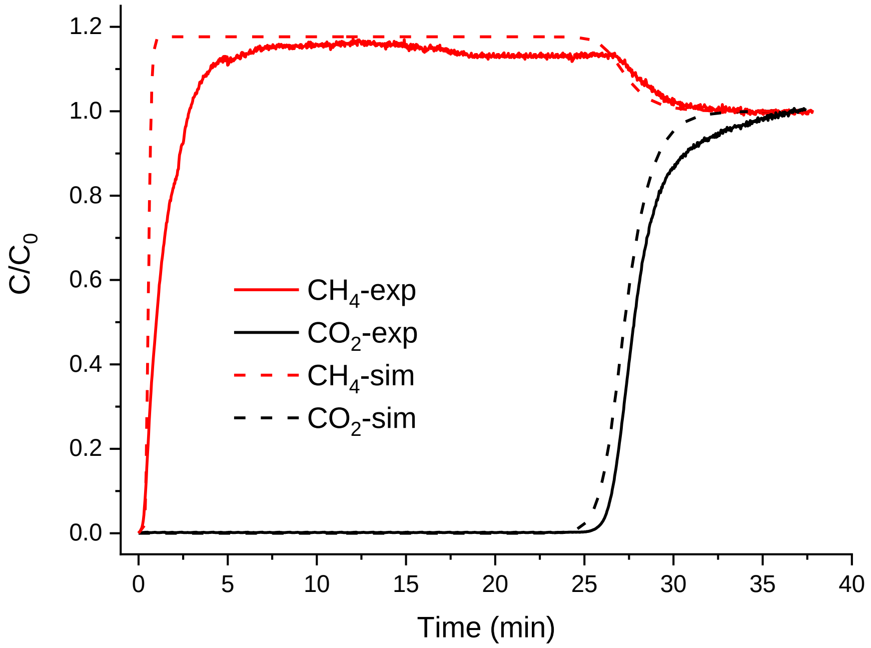

Supplementary Figure 48. Experimental breakthrough curves in comparison to simulated one for a mixture of 15/85 CO_2/CH_4 at 1.1 bar and 293 K in an adsorption column packed with MUF-16.

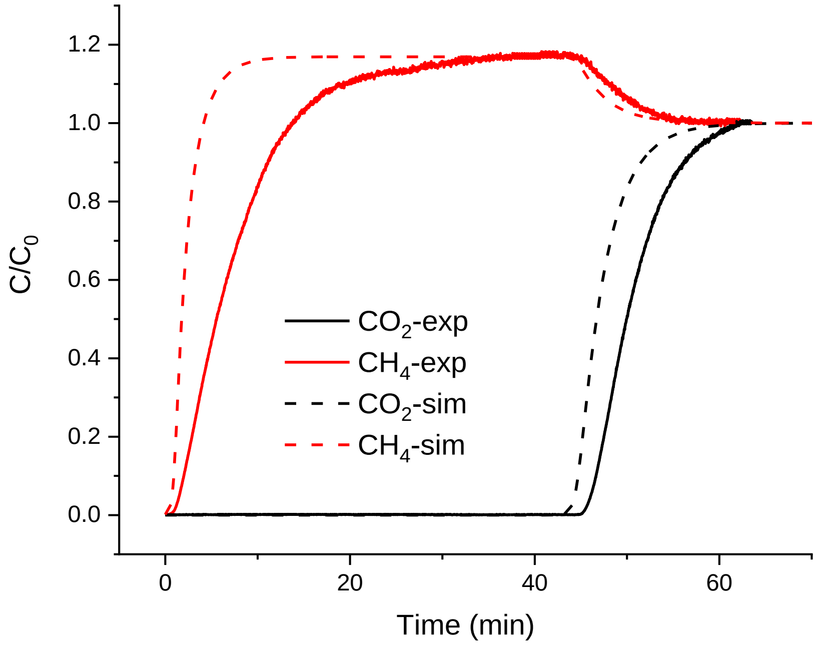

Supplementary Figure 49. Experimental breakthrough curves in comparison to simulated one for a mixture of 15/85 CO_2/CH_4 at 9 bar and 293 K in an adsorption column packed with MUF-16.

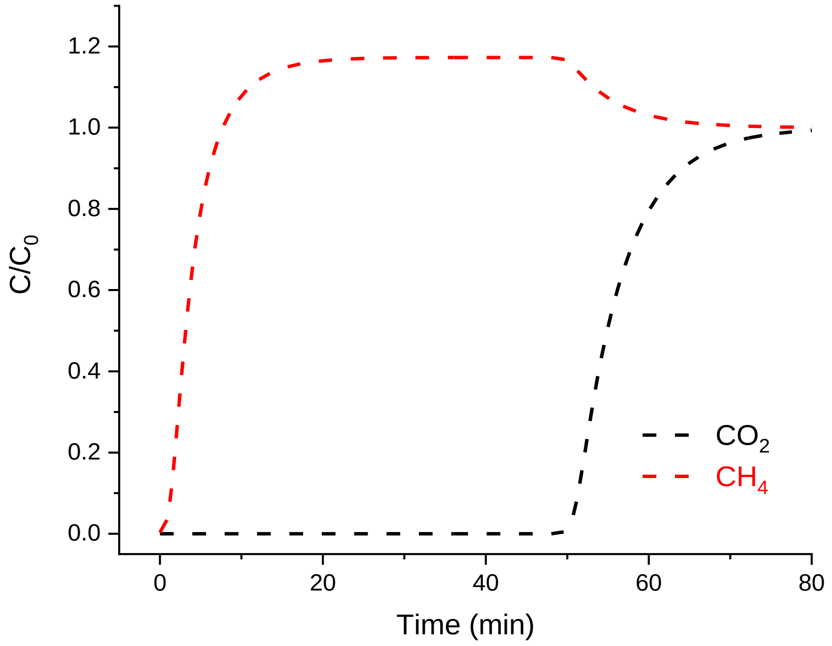

Supplementary Figure 50. Simulated breakthrough curves for a mixture of 15/85 CO_2/CH_4 at 50 bar and 293 K in an adsorption column packed with MUF-16.

10.2 CO_2/C2 hydrocarbon separations

In a typical breakthrough experiment, 0.9 g of activated MUF-16 was placed in an adsorption column (6.4 mm in diameter × 11 cm in length) to form a fixed bed. The adsorbent was activated at 130 °C under high vacuum for 7 hours and then the column was left under vacuum for another 3 hours while being cooled to 20 °C. The column was then purged under a 20 mL_N/min flow of He gas for 1 hr at 1.1 bar prior to the breakthrough experiment. A gas mixture containing different gas pairs of CO_2 and C_2H_2, C_2H_6 or C_2H_4 along with He as a carrier gas was introduced to the column at 1.1 bar and 20 °C. A feed flowrate of 6.0 or 6.85 mL_N/min (including helium) was set for the experiments with 50/50 and 5/95 mixture of gases, respectively, and the flowrate of He in the feed was kept constant at 2 mL_N/min for all the experiments. The operating pressure was controlled at 1.1 bar with a back-pressure regulator. The outlet composition was continuously monitored by a SRS UGA200 mass spectrometer. The CO_2 was deemed to have broken through from the column when its concentration reached 600 ppmv.

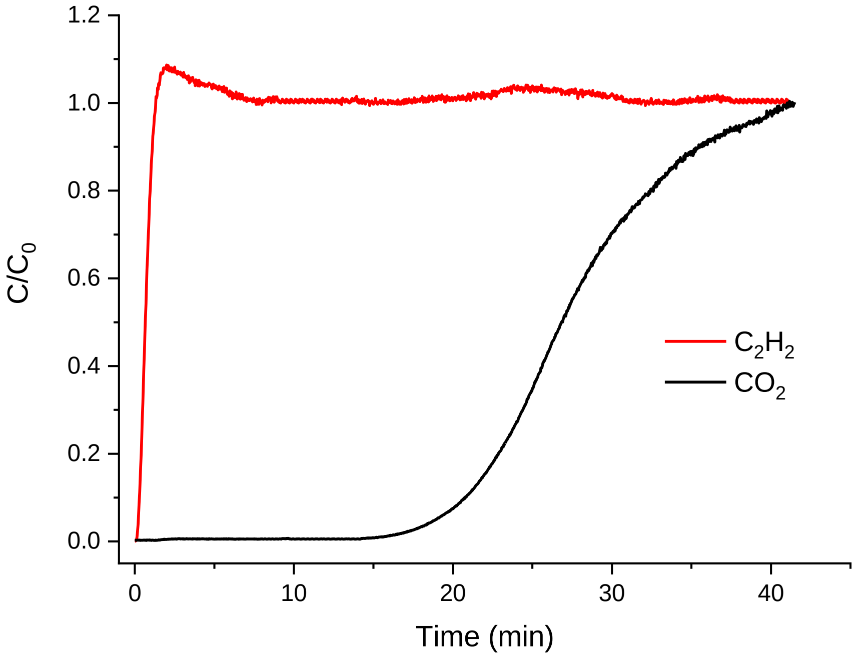

Supplementary Figure 51. Experimental breakthrough curves for a mixture of 5/95 CO_2/C_2H_2 at 1.1 bar and 293 K in an adsorption column packed with MUF-16.

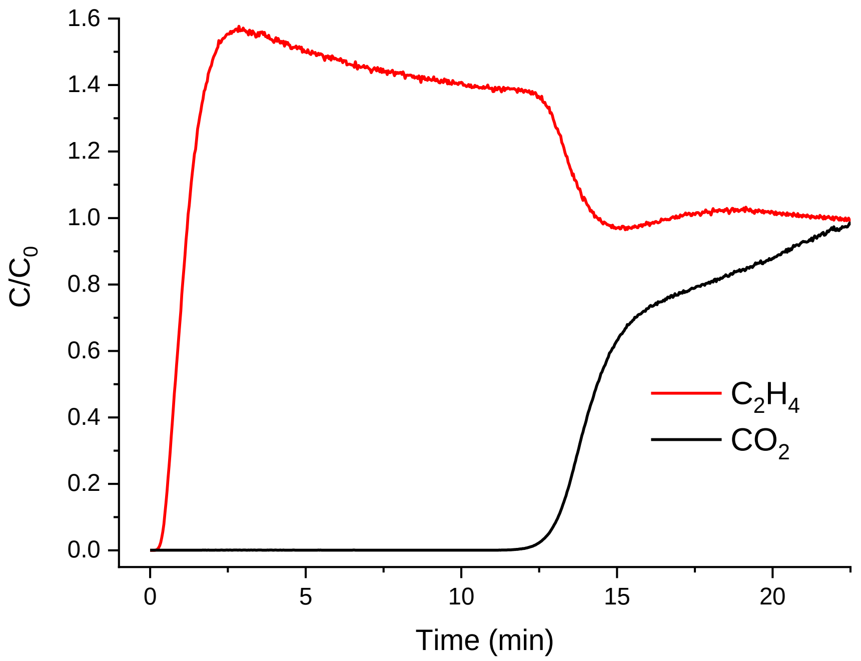

Supplementary Figure 52. Experimental breakthrough curves for a mixture of 50/50 CO_2/C_2H_4 at 1.1 bar and 293 K in an adsorption column packed with MUF-16.

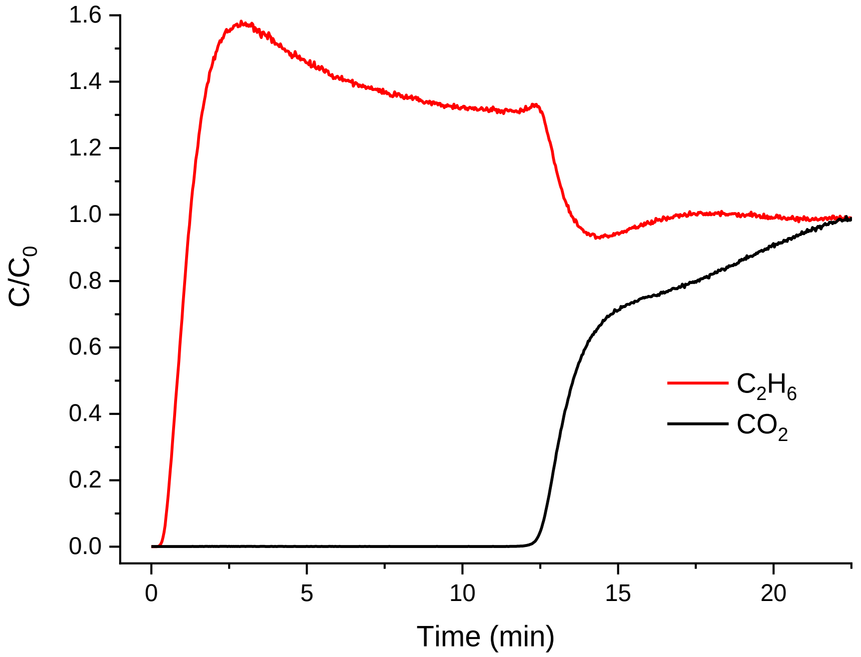

Supplementary Figure 53. Experimental breakthrough curves for a mixture of 50/50 CO_2/C_2H_6 at 1.1 bar and 293 K in an adsorption column packed with MUF-16.

Adsorbent regeneration

The desorption behaviour of CO_2 and C_2H_2 from the adsorption column was also investigated. Once the adsorbent was saturated with an equimolar mixture of CO_2 and C_2H_2, the column was purged with a helium flow of 5 mL_N/min for 18 mins at 20 °C at 1 bar while monitoring the effluent gas. Then the column was then heated to 80 °C with a ramp of 10 °C/min for 20 mins. Finally, the column was heated to 130 °C with the same ramping for 15 min before cooling to 20 °C. A breakthrough measurement was then performed, which showed that the absorbent had been fully regenerated.

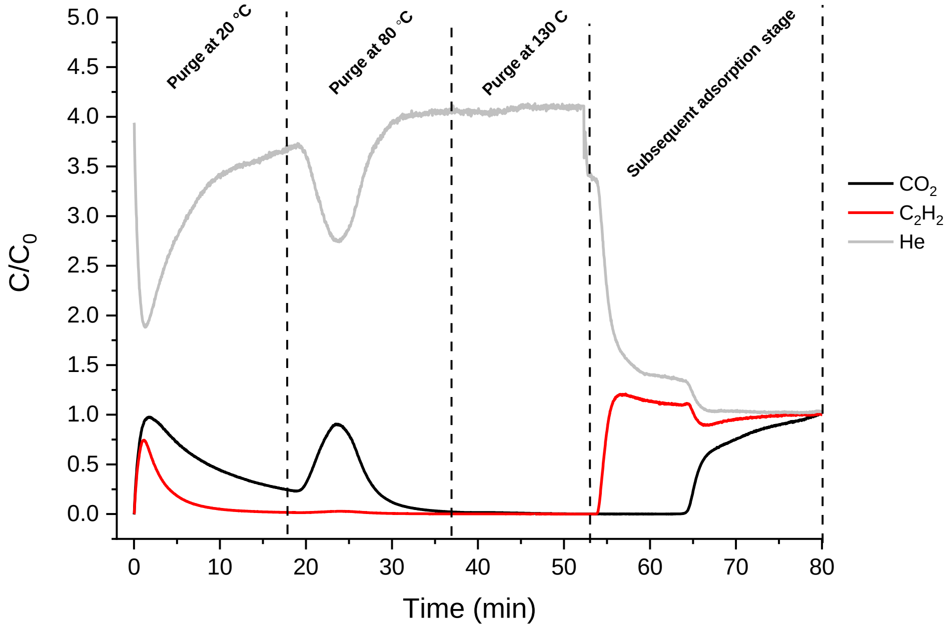

Supplementary Figure S54. Desorption behaviour of the adsorbates through heating the column at 1 bar under a helium flow of 5 mL_N/min. C_2H_2 is fully removed from the bed by purging with helium at room temperature. CO_2 is completely desorbed from the column upon heating to 80 °C with a flow of helium. No adsorbates remained to be removed upon further heating to 130 °C.

10.2.1. Simulations of CO_2/C_2H_2 breakthrough curves

The simulation of breakthrough curves for CO_2/C2 hydrocarbons was carried out using the method reported above. A summary of adsorption column parameters and feed characterizations are presented in Supplementary Table 9.

Supplementary Table 9. Adsorption column parameters and feed characterizations used for the simulations for MUF-16.

Adsorption bed	***Feed***
Length: 110 mm<br>Diameter: 6.4 mm<br>Amount of adsorbent in the bed: 0.9 g<br>Bed voidage: 0.84<br>Adsorbent average radius: 0.2 mm<br>k_{CO2}: 0.021 s^{-1}<br>k_{C2H2}: 0.024 s^{-1}	Flow rates:<br>6 mL_N/min for equimolar and 0.1/99.9 mixtures, and 6.85 mL_N/min for the 5/95 mixture.<br>Temperature: 293 K<br>Pressure: 1.1 bar<br>Carrier gas (He) flow rate: 2 mL_N/min.

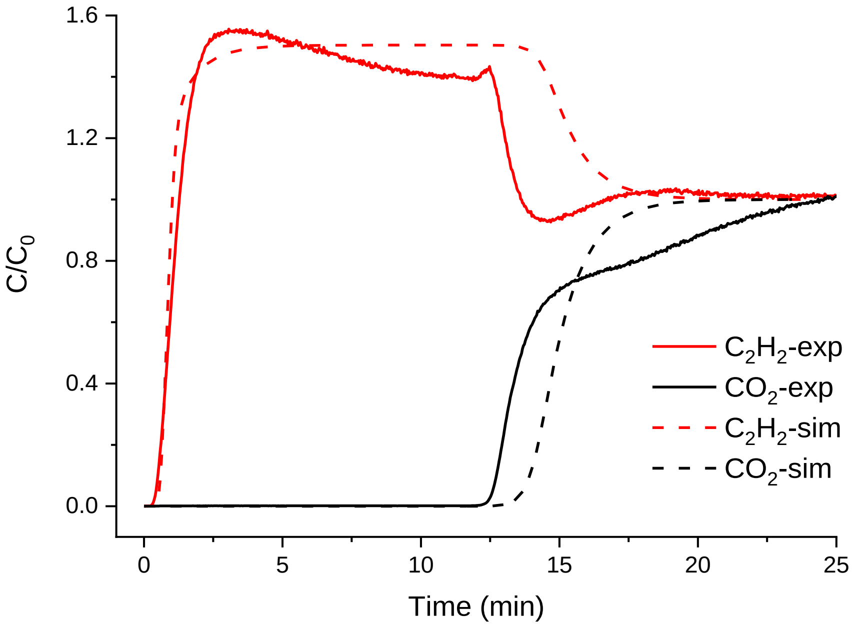

Supplementary Figure 55. Experimental breakthrough curves in comparison to simulated one for a mixture of 50/50 CO_2/C_2H_2 at 1.1 bar and 293 K in an adsorption column packed with MUF-16.

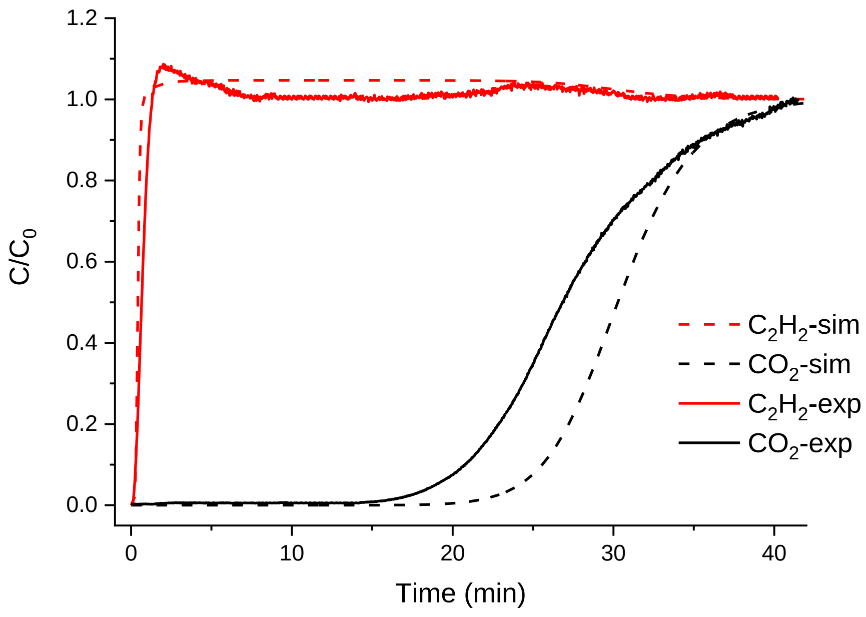

Supplementary Figure 56. Experimental breakthrough curves in comparison to simulated one for a mixture of 5/95 CO_2/C_2H_2 at 1.1 bar and 293 K in an adsorption column packed with MUF-16.

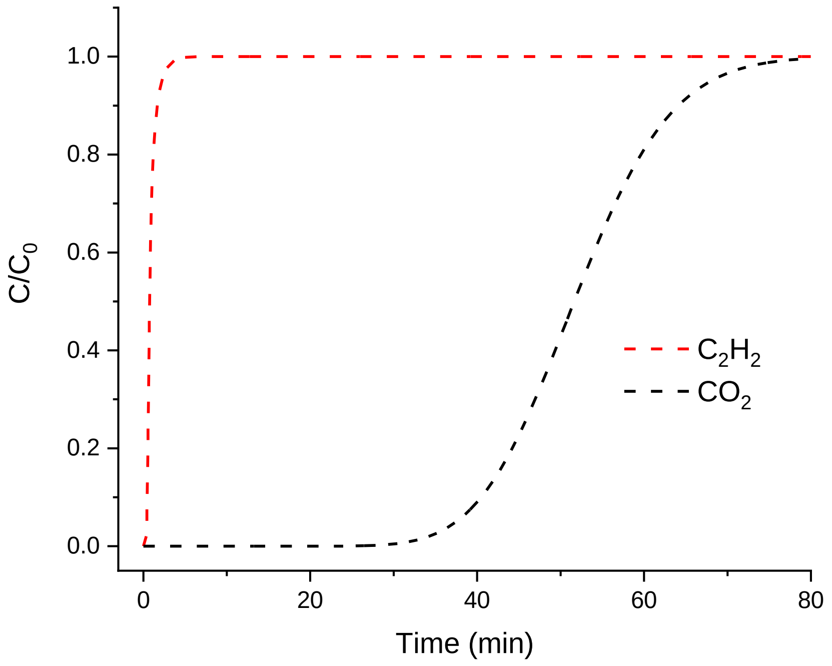

Supplementary Figure 57. Simulated breakthrough curves for a mixture of 0.1/99.9 CO_2/C_2H_2 at 1.1 bar and 293 K in an adsorption column packed with MUF-16.

11. Supplementary Tables: Reported separation metrics

The CO_2/CH_4 and CO_2/C_2H_2 separation parameters of MUF-16 in comparison to other MOFs and related materials are presented in Supplementary Tables 10 and 11. Materials with molecular sieving mechanisms are excluded from this analysis. IAST selectivities are presented for a 50/50 CO_2/CH_4 and CO_2/C_2H_2 at 1 bar, unless otherwise stated. Q_{st} values are reported at low loading, unless otherwise stated. Uptake ratios are calculated by dividing the uptake of CO_2 by that of CH_4 or C_2H_2 (all at 1 bar and the specified temperature in the tables). These were taken from either a direct statement of relevant details in the manuscript or were extracted from figures by a digitizer software.

Supplementary Table 10. Metrics relevant to CO_2/CH_4 separations for MUF-16 in comparison to a selection of materials reported in the literature.

	Material	T (°C)	P (bar)	CO_2 | CH_4 uptakes (cc/g)	$Q_{st}(CO_2)$ (kJ/mol)	Uptake ratio	IAST selectivity (50/50)
This work	**MUF-16**	**20**	**1**	**47.8 | 1.2**	**32**	**39.8**	**6686**
	**MUF-16(Mn)**	**20**	**1**	**50.5 | 3.1**	**37**	**16.3**	**470**
	**MUF-16(Ni)**	**20**	**1**	**48.0 | 2.8**	**37**	**17.3**	**1215**
Selected MOFs, carbons and zeolites	Zeolite 13X[14, 15]	25	1	112 | 13	44-54	8.6	103
	Zeolite 5A[16, 17]	30	1	75.5 | 11.8	23[c]	6.4	n/a
	Zeolite 4A[18, 19]	30-32	1	105.3 | 15	39	7	n/a
	BPL Activated carbon[20, 21]	25	1	46.2 | 20.2	21[c]	2.3	4
	SIFSIX-3-Zn[15]	25	1	57 | 17.6	45	3.2	230
	$[Cd_2L(H_2O)]_2.5H_2O$[22]	20	1	47.2 | 1.1	37	42.9	n/a
	UTSA-120[23]	23	1	112 | 20.8	27	5.4	96
	UTSA-16[24, 25]	23	1	96 | 13.2	33	7.3	38
	HKUST-1[25-27]	25	1	103 | 18.7	35[c]	7.4	5.5
	Mg-dobdc[15, 25, 28, 29]	23	1	190 | 25	47-52	7.6	130
	IITKGP-5a[30]	22	1	49 | 13.6	23	3.6	24
	WOFOUR-1-Ni[31]	25	1	52 | 11.5	66	4.5	26[a]
	SIFSIX-2-Cu-i[15]	25	1	121.2 | 10.5	32	11.5	33
	CAU-1[32]	0	1	165 | 27	48	6.1	28[b]
	NbOFFIVE-Ni[33, 34]	25	1	51.7 | 2.2	54	23.1	366
	TIFSIX-3-Ni[33, 34]	25	1	48.6 | 4.8	50	10.2	158
	SIFSIX-14-Cu-i[35]	20	1	110.5 | 1.3 |	38	85	n/a
	SIFSIX-3-Ni[33, 34]	25	1	64.5 | 6.6	51	8.9	130

Values were generally taken from either a direct statement in the manuscript or were extracted from relevant Figs. by a digitizer software. Materials with molecular sieving mechanisms are excluded from this analysis. [a] IAST is calculated for a 10/90 mixture. [b] Selectivity was calculated from the slope of isotherms at low pressures (Henry constants).[c] Heat of adsorption averaged over CO_2 uptakes.

Supplementary Table 11. Separation metrics relevant to C_2H_2/CO_2 separations for MUF-16 in comparison to selected materials reported in the literature.

MOF	T (°C)	P (bar)	CO_2 uptake (mmol/g)	C_2H_2 uptake (mmol/g)	Q_{st} of CO_2[a] (kJ/mol)	Q_{st} of C_2H_2[a] (kJ/mol)	Uptake ratio*	IAST selectivity (50/50)*
**CO_2-selective MOFs**								
MUF-16	20	1	2.14	0.18	32	25.8	12.0	510
[Mn(bdc)(dpe)][36]	0	1	2.08	0.32	29.5	27.8	6.4	9.0
SIFSIX-3-Ni[37]	25	1	2.80	3.30	51	36.5	0.8	7.5[&]
$K_2[Cr_3O(OOCH)_6$[6]	5	1	0.50	0.10	38	30	4.5	5.6[##]
CD-MOF-1[38]	25	1	2.87	2.23	41	17	1.3	3.4[&]
CD-MOF-2[38]	25	1	2.67	2.03	67.5	25	1.3	6.1[&]
$[Tm_2(OH\text{-}bdc)_2(\mu_3\text{- }OH)_2(H_2O)_2]$[39]	25	1	5.83	2.1	45.2	17.8	2.8	18.2
$[Tm_2(OH\text{-}bdc)_2(\mu_3\text{- }OH)_2]$[39]	25	1	6.21	5.25	32.7	26.0	1.2	1.6
**C_2H_2-selective MOFs**								
UTSA-300a[40]	25	1	0.15	3.10	-	57	20.6	700
NKMOF-1-Ni[41]	25	1	2.27	2.67	41	60	1.2	22
HOF-3a[42]	23	1	0.93	2.14	42	19.5	2.3	21
$[Ni_3(HCOO)_6]$[43]	25	1	3.00	4.20	24.5	41	1.4	21
SNNU-45[44]	25	1	4.34	5.98	27.1	40	1.37	4.5
ZJU-196a[45]	25	1	0.35	3.70	-	39	10.6	18
JCM-1[46]	25	1	1.69	3.34	33	36.5	2.0	14
DICRO-4-Ni-i[47]	25	1	1.02	1.91	34	38	1.9	13.5
UTSA-74a[48]	25	1	3.00	4.80	25.5	31.5	1.6	8
TIFSIX-2-Cu-i[37]	25	1	4.20	4.10	36	46	0.97	6
Cu-BTC[25, 42, 49]	25	1	5.10	8.90	26.9	30	1.7	5.5
MAF-2[50]	25	1	0.82	3.90	27	33	4.7	5
UTSA-50a[42]	23	1	3.10	4.10	27.8	32	1.3	5
FJU-90a[51]	25	1	4.92	8.03	21	25	1.6	4.3
ZJU-60a[52]	23	1	3.12	6.69	15.5	17.5	2.1	4
ZJU-10a[53]	25	1	3.66	7.58	26	39	2.1	4
MFM-188[54]	25	1	5.35	10.20	20.8	32.5	1.9	3.7
FeNi-M'MOF[55]	25	1	2.72	4.29	24.5	32.8	1.6	22.5

Values were taken from either a direct statement in the manuscript or were extracted from relevant Figs. by a digitizer software. [a] Q_{st} at low coverage. * Uptake ratios and IAST selectivities are given with respect to the ratio of the highly adsorbed component to the weakly adsorbed component from an equimolar mixture. [&] isotherms or fitting parameters were taken from literatures for 3-Ni[37], CD-MOFs[38], and $[Tm_2(OH\text{-}bdc)_2(\mu_3\text{- }OH)_2(H_2O)_2]$[39] to calculate IAST selectivity for an equimolar mixture. [##] Isotherm data were extracted from [6] using a digitizer software so we could calculate IAST selectivity for an equimolar mixture.

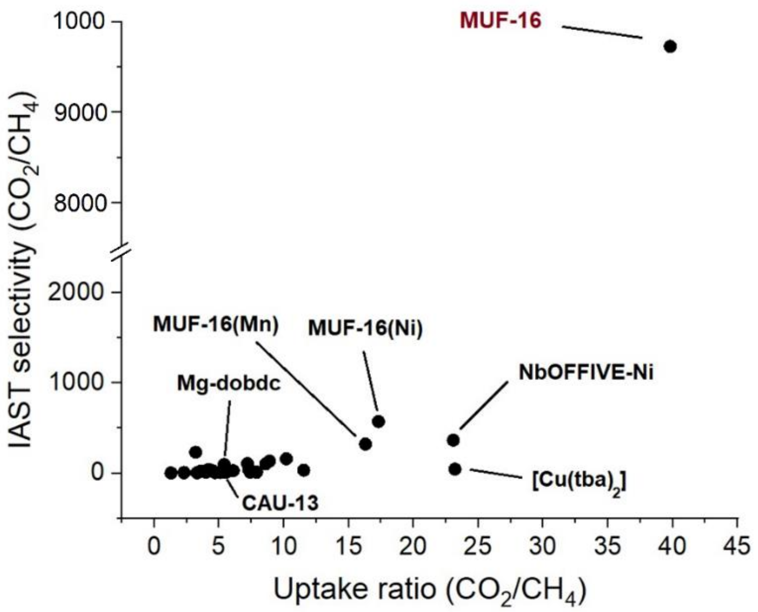

Supplementary Figure 58. IAST selectivity of MUF-16 family for an equimolar mixture of CO_2/CH_4 in comparison to top top-performing MOFs at 1 bar and ambient temperature versus their uptake ratio at 1 bar.

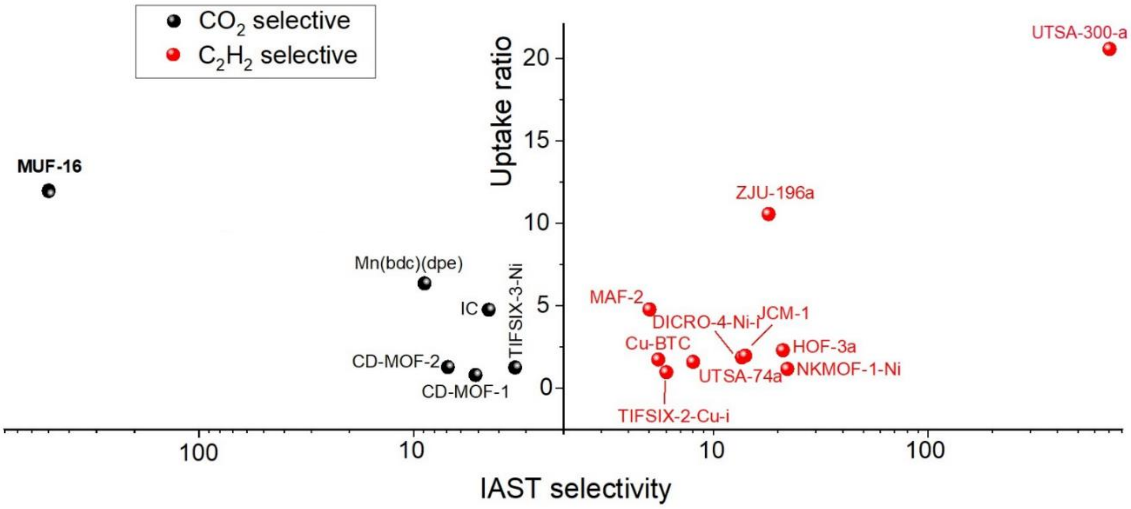

Supplementary Figure 59. Predicted IAST selectivity (log scale) from an equimolar mixture of CO_2/C_2H_2 plotted against uptake ratio at 1 bar and 293-298 K (except for IC (278 K) and [Mn(bdc)(dpe)] (273 K)) for MUF-16 in comparison to the best materials reported to date. Selectivity and uptake ratios are defined as CO_2/C_2H_2 and C_2H_2/CO_2 for CO_2-selective and C_2H_2-selective materials, respectively.

12. Supplementary Methods: DFT calculations

The lowest energy configuration of the CO_2 binding sites in the *P*1 form of MUF-16(Mn) were calculated using density functional theory (DFT) with the software package VASP 5.4.4.[56] One guest molecule was admitted per cell. We implemented dispersion corrections using the DFT-D3 method,[57] as standard DFT methods based on generalized gradient approximation do not fully account for the long-range dispersion interactions between the framework and the bound adsorbate. Electron exchange and correlation were described using the generalized gradient approximation Perdew, Burke, and Ernzerhof (PBE)[58] form, and the projector-augmented wave potentials were used to treat core and valence electrons. In all cases, we used a plane-wave kinetic energy cutoff of 650 eV and a Gamma-point mesh for sampling the Brillouin zone. The ionic coordinates were relaxed until the Hellman-Feynman ionic forces were less than 0.02 eV/Å. The lattice parameters (unit cell) were fixed to the experimental values.

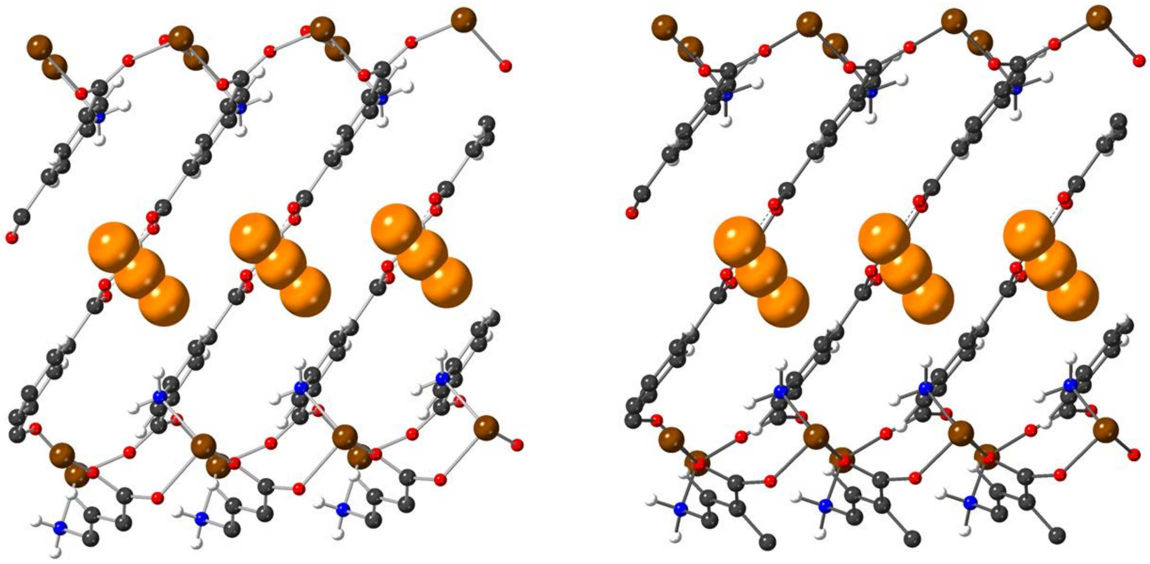

Supplementary Figure 60. A comparison of the structures of MUF-16(Mn)·CO2 determined by SCXRD (left) and DFT (right). Only one orientation of the CO_2 guest, which is disordered by symmetry, is shown in the experimental structure. The CO_2 is highlighted as orange balls. Colour code: Mn = brown; O = red; C = dark grey, N = blue, H = white.

13. Supplementary References

1. Tang, E.; Dai, Y.-M.; Zhang, J.; Li, Z.-J.; Yao, Y.-G.; Zhang, J.; Huang, X.-D., Two Cobalt(II) 5-Aminoisophthalate Complexes and Their Stable Supramolecular Microporous Frameworks. *Inorg. Chem.* **2006,** *45* (16), 6276-6281.
2. Tian, C.-B.; He, C.; Han, Y.-H.; Wei, Q.; Li, Q.-P.; Lin, P.; Du, S.-W., Four New MnII Inorganic–Organic Hybrid Frameworks with Diverse Inorganic Magnetic Chain's Sequences: Syntheses, Structures, Magnetic, NLO, and Dielectric Properties. *Inorg. Chem.* **2015,** *54* (6), 2560-2571.
3. Willems, T. F.; Rycroft, C. H.; Kazi, M.; Meza, J. C.; Haranczyk, M., Algorithms and tools for high-throughput geometry-based analysis of crystalline porous materials. *Microporous Mesoporous Mater.* **2012,** *149* (1), 134-141.
4. Dubbeldam, D.; Calero, S.; Ellis, D. E.; Snurr, R. Q., RASPA: molecular simulation software for adsorption and diffusion in flexible nanoporous materials. *Mol. Simul.* **2016,** *42* (2), 81-101.
5. Li, J.-R.; Kuppler, R. J.; Zhou, H.-C., Selective gas adsorption and separation in metal–organic frameworks. *Chem. Soc. Rev.* **2009,** *38* (5), 1477-1504.
6. Eguchi, R.; Uchida, S.; Mizuno, N., Inverse and High CO_2/C_2H_2 Sorption Selectivity in Flexible Organic–Inorganic Ionic Crystals. *Angew. Chem., Int. Ed.* **2012,** *51* (7), 1635-1639.
7. Reid, C. R.; Thomas, K. M., Adsorption Kinetics and Size Exclusion Properties of Probe Molecules for the Selective Porosity in a Carbon Molecular Sieve Used for Air Separation. *J. Phys. Chem. B* **2001,** *105* (43), 10619-10629.
8. Li, L.; Lin, R.-B.; Wang, X.; Zhou, W.; Jia, L.; Li, J.; Chen, B., Kinetic separation of propylene over propane in a microporous metal-organic framework. *Chem. Eng. J.* **2018,** *354*, 977-982.
9. Walton, K. S.; Snurr, R. Q., Applicability of the BET Method for Determining Surface Areas of Microporous Metal-Organic Frameworks. *J. Am. Chem. Soc.* **2007,** *129*, 8552-8558.
10. Dincă, M.; Dailly, A.; Liu, Y.; Brown, C. M.; Neumann, D. A.; Long, J. R., Hydrogen Storage in a Microporous Metal−Organic Framework with Exposed Mn2+ Coordination Sites. *J. Am. Chem. Soc.* **2006,** *128* (51), 16876-16883.
11. Myers, A.; Prausnitz, J. M., Thermodynamics of mixed ‐ gas adsorption. *AIChE J.* **1965,** *11* (1), 121-127.
12. Qazvini, O. T.; Babarao, R.; Shi, Z.-L.; Zhang, Y.-B.; Telfer, S. G., A Robust Ethane-Trapping Metal–Organic Framework with a High Capacity for Ethylene Purification. *J. Am. Chem. Soc.* **2019,** *141* (12), 5014–5020.
13. Qazvini, O. T.; Babarao, R.; Telfer, S. G., Multipurpose Metal–Organic Framework for the Adsorption of Acetylene: Ethylene Purification and Carbon Dioxide Removal. *Chem. Mater.* **2019,** *31* (13), 4919-4926.
14. Cavenati, S.; Grande, C. A.; Rodrigues, A. E., Adsorption Equilibrium of Methane, Carbon Dioxide, and Nitrogen on Zeolite 13X at High Pressures. *J. Chem. Eng. Data* **2004,** *49* (4), 1095-1101.
15. Nugent, P.; Belmabkhout, Y.; Burd, S. D.; Cairns, A. J.; Luebke, R.; Forrest, K.; Pham, T.; Ma, S.; Space, B.; Wojtas, L.; Eddaoudi, M.; Zaworotko, M. J., Porous materials with optimal adsorption thermodynamics and kinetics for CO2 separation. *Nature* **2013,** *495*, 80.
16. Saha, D.; Bao, Z.; Jia, F.; Deng, S., Adsorption of CO2, CH4, N2O, and N2 on MOF-5, MOF-177, and Zeolite 5A. *Environ. Sci. Technol.* **2010,** *44* (5), 1820-1826.
17. Nam, G.-M.; Jeong, B.-M.; Kang, S.-H.; Lee, B.-K.; Choi, D.-K., Equilibrium Isotherms of CH4, C2H6, C2H4, N2, and H2 on Zeolite 5A Using a Static Volumetric Method. *J. Chem. Eng. Data* **2005,** *50* (1), 72-76.

18. Shao, W.; Zhang, L.; Li, L.; Lee, R. L., Adsorption of CO_2 and N_2 on synthesized NaY zeolite at high temperatures. *Adsorption* **2009,** *15* (5), 497.
19. Ahmed, M. J.; Theydan, S. K., Equilibrium isotherms studies for light hydrocarbons adsorption on 4A molecular sieve zeolite. *Journal of Petroleum Science and Engineering* **2013,** *108,* 316-320.
20. McEwen, J.; Hayman, J.-D.; Ozgur Yazaydin, A., A comparative study of CO2, CH4 and N2 adsorption in ZIF-8, Zeolite-13X and BPL activated carbon. *Chem. Phys.* **2013,** *412,* 72-76.
21. Delgado, J. A.; Águeda, V. I.; Uguina, M. A.; Sotelo, J. L.; Brea, P.; Grande, C. A., Adsorption and Diffusion of H2, CO, CH4, and CO2 in BPL Activated Carbon and 13X Zeolite: Evaluation of Performance in Pressure Swing Adsorption Hydrogen Purification by Simulation. *Ind. Eng. Chem. Res.* **2014,** *53* (40), 15414-15426.
22. Hou, L.; Shi, W.-J.; Wang, Y.-Y.; Guo, Y.; Jin, C.; Shi, Q.-Z., A rod packing microporous metal–organic framework: unprecedented ukv topology, high sorption selectivity and affinity for CO_2. *Chem. Commun.* **2011,** *47* (19), 5464-5466.
23. Wen, H.-M.; Liao, C.; Li, L.; Alsalme, A.; Alothman, Z.; Krishna, R.; Wu, H.; Zhou, W.; Hu, J.; Chen, B., A metal–organic framework with suitable pore size and dual functionalities for highly efficient post-combustion CO_2 capture. *J. Mat. Chem. A* **2019,** *7,* 3128-3134.
24. Masala, A.; Vitillo, J. G.; Mondino, G.; Grande, C. A.; Blom, R.; Manzoli, M.; Marshall, M.; Bordiga, S., CO2 Capture in Dry and Wet Conditions in UTSA-16 Metal–Organic Framework. *ACS Appl. Mater. Interfaces* **2017,** *9* (1), 455-463.
25. Xiang, S.; He, Y.; Zhang, Z.; Wu, H.; Zhou, W.; Krishna, R.; Chen, B., Microporous metal-organic framework with potential for carbon dioxide capture at ambient conditions. *Nat. Commun.* **2012,** *3,* 954.
26. Liang, Z.; Marshall, M.; Chaffee, A. L., CO2 Adsorption-Based Separation by Metal Organic Framework (Cu-BTC) versus Zeolite (13X). *Energy Fuels* **2009,** *23* (5), 2785-2789.
27. Liu, B.; Smit, B., Comparative Molecular Simulation Study of CO2/N2 and CH4/N2 Separation in Zeolites and Metal−Organic Frameworks. *Langmuir* **2009,** *25* (10), 5918-5926.
28. Mason, J. A.; Sumida, K.; Herm, Z. R.; Krishna, R.; Long, J. R., Evaluating metal–organic frameworks for post-combustion carbon dioxide capture via temperature swing adsorption. *Energy Environ. Sci.* **2011,** *4* (8), 3030-3040.
29. Herm, Z. R.; Swisher, J. A.; Smit, B.; Krishna, R.; Long, J. R., Metal− organic frameworks as adsorbents for hydrogen purification and precombustion carbon dioxide capture. *J. Am. Chem. Soc.* **2011,** *133* (15), 5664-5667.
30. Pal, A.; Chand, S.; Elahi, S. M.; Das, M. C., A microporous MOF with a polar pore surface exhibiting excellent selective adsorption of CO2 from CO2–N2 and CO2–CH4 gas mixtures with high CO2 loading. *Dalton Trans.* **2017,** *46* (44), 15280-15286.
31. Mohamed, M. H.; Elsaidi, S. K.; Pham, T.; Forrest, K. A.; Tudor, B.; Wojtas, L.; Space, B.; Zaworotko, M. J., Pillar substitution modulates CO2 affinity in “mmo” topology networks. *Chem. Commun.* **2013,** *49* (84), 9809-9811.
32. Si, X.; Jiao, C.; Li, F.; Zhang, J.; Wang, S.; Liu, S.; Li, Z.; Sun, L.; Xu, F.; Gabelica, Z.; Schick, C., High and selective CO2 uptake, H2storage and methanol sensing on the amine-decorated 12-connected MOF CAU-1. *Energy Environ. Sci.* **2011,** *4* (11), 4522-4527.
33. Madden, D. G.; O’Nolan, D.; Chen, K.-J.; Hua, C.; Kumar, A.; Pham, T.; Forrest, K. A.; Space, B.; Perry, J. J.; Khraisheh, M., Highly selective CO_2 removal for one-step liquefied natural gas processing by physisorbents. *Chem. Commun.* **2019,** *55* (22), 3219-3222.
34. Mukherjee, S.; Sikdar, N.; O’Nolan, D.; Franz, D. M.; Gascón, V.; Kumar, A.; Kumar, N.; Scott, H. S.; Madden, D. G.; Kruger, P. E., Trace CO2 capture by an

ultramicroporous physisorbent with low water affinity. *Science Advances* **2019,** *5* (11), eaax9171.
35. Jiang, M.; Li, B.; Cui, X.; Yang, Q.; Bao, Z.; Yang, Y.; Wu, H.; Zhou, W.; Chen, B.; Xing, H., Controlling Pore Shape and Size of Interpenetrated Anion-Pillared Ultramicroporous Materials Enables Molecular Sieving of CO_2 Combined with Ultrahigh Uptake Capacity. *ACS Appl. Mater. Interfaces* **2018,** *10* (19), 16628-16635.
36. Foo, M. L.; Matsuda, R.; Hijikata, Y.; Krishna, R.; Sato, H.; Horike, S.; Hori, A.; Duan, J.; Sato, Y.; Kubota, Y.; Takata, M.; Kitagawa, S., An Adsorbate Discriminatory Gate Effect in a Flexible Porous Coordination Polymer for Selective Adsorption of CO_2 over C_2H_2. *J. Am. Chem. Soc.* **2016,** *138* (9), 3022-3030.
37. Chen, K.-J.; Scott, H. S.; Madden, D. G.; Pham, T.; Kumar, A.; Bajpai, A.; Lusi, M.; Forrest, K. A.; Space, B.; Perry, J. J.; Zaworotko, M. J., Benchmark C_2H_2/CO_2 and CO_2/C_2H_2 Separation by Two Closely Related Hybrid Ultramicroporous Materials. *Chem* **2016,** *1* (5), 753-765.
38. Li, L.; Wang, J.; Zhang, Z.; Yang, Q.; Yang, Y.; Su, B.; Bao, Z.; Ren, Q., Inverse Adsorption Separation of CO2/C2H2 Mixture in Cyclodextrin-Based Metal–Organic Frameworks. *ACS Appl. Mater. Interfaces* **2019,** *11* (2), 2543-2550.
39. Ma, D.; Li, Z.; Zhu, J.; Zhou, Y.; Chen, L.; Mai, X.; Liufu, M.; Wu, Y.; Li, Y., Inverse and highly selective separation of CO2/C2H2 on a thulium–organic framework. *J. Mater. Chem. A* **2020,** *8* (24), 11933-11937.
40. Lin, R.-B.; Li, L.; Wu, H.; Arman, H.; Li, B.; Lin, R.-G.; Zhou, W.; Chen, B., Optimized separation of acetylene from carbon dioxide and ethylene in a microporous material. *J. Am. Chem. Soc.* **2017,** *139* (23), 8022-8028.
41. Peng, Y.-L.; Pham, T.; Li, P.; Wang, T.; Chen, Y.; Chen, K.-J.; Forrest, K. A.; Space, B.; Cheng, P.; Zaworotko, M. J.; Zhang, Z., Robust Ultramicroporous Metal–Organic Frameworks with Benchmark Affinity for Acetylene. *Angew. Chem., Int. Ed.* **2018,** *57* (34), 10971-10975.
42. Li, P.; He, Y.; Zhao, Y.; Weng, L.; Wang, H.; Krishna, R.; Wu, H.; Zhou, W.; O'Keeffe, M.; Han, Y.; Chen, B., A Rod-Packing Microporous Hydrogen-Bonded Organic Framework for Highly Selective Separation of C_2H_2/CO_2 at Room Temperature. *Angew. Chem., Int. Ed.* **2015,** *54* (2), 574-577.
43. Zhang, L.; Jiang, K.; Zhang, J.; Pei, J.; Shao, K.; Cui, Y.; Yang, Y.; Li, B.; Chen, B.; Qian, G., Low-cost and high-performance microporous metal–organic framework for separation of acetylene from Carbon dioxide. *ACS Sustain Chem Eng* **2018,** *7* (1), 1667-1672.
44. Tu, B.; Pang, Q.; Wu, D.; Song, Y.; Weng, L.; Li, Q., Ordered vacancies and their chemistry in metal-organic frameworks. *J. Am. Chem. Soc.* **2014,** *136* (41), 14465-71.
45. Zhang, L.; Jiang, K.; Li, L.; Xia, Y.-P.; Hu, T.-L.; Yang, Y.; Cui, Y.; Li, B.; Chen, B.; Qian, G., Efficient separation of C_2H_2 from C_2H_2/CO_2 mixtures in an acid–base resistant metal–organic framework. *Chem. Commun.* **2018,** *54* (38), 4846-4849.
46. Lee, J.; Chuah, C. Y.; Kim, J.; Kim, Y.; Ko, N.; Seo, Y.; Kim, K.; Bae, T. H.; Lee, E., Separation of Acetylene from Carbon Dioxide and Ethylene by a Water-Stable Microporous Metal–Organic Framework with Aligned Imidazolium Groups inside the Channels. *Angewandte Chemie International Editions* **2018,** *57* (26), 7869-7873.
47. Scott, H. S.; Shivanna, M.; Bajpai, A.; Madden, D. G.; Chen, K.-J.; Pham, T.; Forrest, K. A.; Hogan, A.; Space, B.; Perry Iv, J. J.; Zaworotko, M. J., Highly Selective Separation of C_2H_2 from CO_2 by a New Dichromate-Based Hybrid Ultramicroporous Material. *ACS Appl. Mater. Interfaces* **2017,** *9* (39), 33395-33400.
48. Luo, F.; Yan, C.; Dang, L.; Krishna, R.; Zhou, W.; Wu, H.; Dong, X.; Han, Y.; Hu, T.-L.; O'Keeffe, M.; Wang, L.; Luo, M.; Lin, R.-B.; Chen, B., UTSA-74: A MOF-74

Isomer with Two Accessible Binding Sites per Metal Center for Highly Selective Gas Separation. *J. Am. Chem. Soc.* **2016,** *138* (17), 5678-5684.
49. Xiang, S.; Zhou, W.; Gallegos, J. M.; Liu, Y.; Chen, B., Exceptionally High Acetylene Uptake in a Microporous Metal−Organic Framework with Open Metal Sites. *J. Am. Chem. Soc.* **2009,** *131* (34), 12415-12419.
50. Zhang, J.-P.; Chen, X.-M., Optimized Acetylene/Carbon Dioxide Sorption in a Dynamic Porous Crystal. *J. Am. Chem. Soc.* **2009,** *131* (15), 5516-5521.
51. Ye, Y.; Ma, Z.; Lin, R.-B.; Krishna, R.; Zhou, W.; Lin, Q.; Zhang, Z.; Xiang, S.; Chen, B., Pore Space Partition within a Metal–Organic Framework for Highly Efficient C_2H_2/CO_2 Separation. *J. Am. Chem. Soc.* **2019,** *141* (9), 4130-4136.
52. Duan, X.; Zhang, Q.; Cai, J.; Yang, Y.; Cui, Y.; He, Y.; Wu, C.; Krishna, R.; Chen, B.; Qian, G., A new metal–organic framework with potential for adsorptive separation of methane from carbon dioxide, acetylene, ethylene, and ethane established by simulated breakthrough experiments. *J. Mat. Chem. A* **2014,** *2* (8), 2628-2633.
53. Duan, X.; Wang, H.; Ji, Z.; Cui, Y.; Yang, Y.; Qian, G., A novel metal-organic framework for high storage and separation of acetylene at room temperature. *J. Solid State Chem.* **2016,** *241*, 152-156.
54. Moreau, F.; da Silva, I.; Al Smail, N. H.; Easun, T. L.; Savage, M.; Godfrey, H. G. W.; Parker, S. F.; Manuel, P.; Yang, S.; Schröder, M., Unravelling exceptional acetylene and carbon dioxide adsorption within a tetra-amide functionalized metal-organic framework. *Nat. Commun.* **2017,** *8*, 14085.
55. Gao, J.; Qian, X.; Lin, R.-B.; Krishna, R.; Wu, H.; Zhou, W.; Chen, B., Mixed Metal–Organic Framework with Multiple Binding Sites for Efficient C2H2/CO2 Separation. *Angew. Chem., Int. Ed.* **2020,** *59* (11), 4396-4400.
56. Kresse, G.; Hafner, J., Ab initio molecular dynamics for open-shell transition metals. *Phys. Rev. B* **1993,** *48* (17), 13115.
57. Grimme, S.; Ehrlich, S.; Goerigk, L., Effect of the damping function in dispersion corrected density functional theory. *J. Comput. Chem.* **2011,** *32* (7), 1456-1465.
58. Perdew, J. P.; Burke, K.; Ernzerhof, M., Generalized gradient approximation made simple. *Phys. Rev. Lett.* **1996,** *77* (18), 3865.
